# Asymmetric polyamide nanofilms with highly ordered nanovoids for water purification

**DOI:** 10.1038/s41467-020-19809-3

**Published:** 2020-11-30

**Authors:** Bingbing Yuan, Shengchao Zhao, Ping Hu, Jiabao Cui, Q. Jason Niu

**Affiliations:** 1 School of Chemistry and Chemical Engineering, Key Laboratory of Green Chemical Media and Reactions, Ministry of Education, Henan Normal University, 453007 Xinxiang, Henan China; 2 Institute for Advanced Study, Shenzhen University, 518060 Shenzhen, Guangdong China; 3 State Key Laboratory of Heavy Oil Processing, College of Chemical Engineering, China University of Petroleum (East China), 266555 Qingdao, Shandong China

**Keywords:** Dendrimers, Nanocomposites, Polymers

## Abstract

Tailor-made structure and morphology are critical to the highly permeable and selective polyamide membranes used for water purification. Here we report an asymmetric polyamide nanofilm having a two-layer structure, in which the lower is a spherical polyamide dendrimer porous layer, and the upper is a polyamide dense layer with highly ordered nanovoids structure. The dendrimer porous layer was covalently assembled in situ on the surface of the polysulfone (PSF) support by a diazotization-coupling reaction, and then the asymmetric polyamide nanofilm with highly ordered hollow nanostrips structure was formed by interfacial polymerization (IP) thereon. Tuning the number of the spherical dendrimer porous layers and IP time enabled control of the nanostrips morphology in the polyamide nanofilm. The asymmetric polyamide membrane exhibits a water flux of 3.7−4.3 times that of the traditional monolayer polyamide membrane, showing an improved divalent salt rejection rate (more than 99%), which thus surpasses the upper bound line of the permeability−selectivity performance of the existing various structural polyamide membranes. We estimate that this work might inspire the preparation of highly permeable and selective reverse osmosis (RO), organic solvent nanofiltration (OSNF) and pervaporation (PV) membranes.

## Abstract (web summary)

Structure and morphology are critical to the performance of permeable and selective polyamide membranes in water purification. Here, the authors report a two layer asymmetric polyamide nanofilm in which a spherical polyamide dendrimer porous lower and a polyamide dense upper layer form hierarchical nanovoids.

## Introduction

Shortage and contamination issues of water resources induced by population explosion and industrialization, make the global economic and social sustainable development face huge challenges^1–3^. Membrane technology, characterized by energy efficiency and technologically mature, has evolved to be the mainstream desalination process^4^. Specifically, polyamide composite membranes such as reverse osmosis (RO) and nanofiltration (NF) membranes, fabricated by IP process, have been widely used in seawater desalination, water softening, and industrial wastewater treatment processes due to the good concentrating and separation performance^5,6^. In general, the existing polyamide composite membrane is a three-layer structure composed of non-woven, ultrafiltration porous support, and polyamide dense layer. Non-woven and the ultrafiltration layer with sponge-like structure serves as support, while the dense polyamide nanofilm imparts the separation and concentration features^6^. However, the permeation-selectivity upper-bound line of current polyamide composite membranes is limited by the intrinsic morphology and structure of the dense separation layer, resulting in a great energy consumption during real application^7–9^. It has been confirmed that when the permeability of the current polyamide membrane is increased by three times, the number of pressure vessels of seawater reverse osmosis membrane (SWRO) can be reduced by 44%, and the number of pressure vessels of brackish water reverse osmosis membrane (BWRO) can be reduced by 63%. The corresponding consumption is also reduced by 15 and 46%^10^. Therefore, innovating the structure and morphology of polyamide nanofilm to improve permeability and selectivity, and thus reduce the investment cost and operating energy consumption of RO and NF membranes, is a critical issue^11^.

It is well known that the permeability and selectivity of polyamide composite membrane are primarily related to its structural morphology and ultrafiltration support performance. Extensive efforts have been dedicated to tailoring the structural morphology of the polyamide dense layer. Tuning the monomer formula^12–15^, fabrication process^16–19^ and porous support structural property^20,21^ are the usual methods, which mainly generates two effects, one is to form a thinner polyamide dense layer, the other is to increase the specific surface area via nanoscale voids formation. The thinner polyamide dense layer aims to lower the resistance of water transport across the active layer, and thereby improve permeance. For example, employing the molecular layer-by-layer^22,23^, 3D printing process^24,25^, introducing an interlayer on ultrafiltration (UF) or microfiltration (MF) substrates^26,27^, or incorporating hydrophilic nanomaterials^28^ during IP have all been utilized to form an ultra-thin polyamide nanofilm. The interlayer on UF support, such as inorganic nanostrand film^17^, carbon nanotube network layer^20,21,27,29^, covalent organic framework nanosheets^26^, and cellulose nanocrystals film^30^, can precisely control interfacial reaction and obtain a thin dense layer.

Increasing the specific surface area and creating enough nanoscale voids is to achieve more salt/water cross-flow mass on the polyamide dense layer, and thereby enhance the water permeance per unit area. The study found that the porosity of the aromatic polyamide nanofilm is between 15 ± 2% and 32 ± 4%^31^. Incorporating hydrophilic additives during IP^28^, modifying the surface property of ultrafiltration support^32,33^ or templated method^16^ can form the polyamide nanofilm with high specific surface area. The latest research indicates that the wrinkle on the polyamide dense layers originates from the diffusion-driven instability of amine molecules during IP process, resulting in a high specific surface area^28^. Hydrophilic additives such as polyvinyl alcohol (PVA), are able to interact with amine molecule via hydrogen bonding and increase solution viscosity, then reduce the diffusion rate, and further form a crumpled polyamide structure. In addition, the inhomogeneous surface properties of UF support such as pore size and water wetting behavior, imparts an unusual distribution of amine solutions, also makes an inconsonant diffusion-reaction rate, and thus forms a crumpled polyamide layer with nanoscale voids. Ideally, the highly ordered nanoscale voids formed in the defected-free and crumpled polyamide dense layer is to rationally achieve an optimized water transport path, and thus provides a potential to surpass the permeability−selectivity upper-bound line of the existing polyamide membrane.

Herein we report an asymmetric polyamide nanofilm, as example by a polyamide NF membrane, in which the lower is a spherical dendrimer polyamide porous layer and the upper is a dense and uniform nano-stripe polyamide nanofilm with highly ordered nanovoids. The porous layer with spherical dendrimer was formed through the assembly of the terminated amines from the dendrimer molecules via a diazotization-coupling reaction, which was conducted in situ on the support surface. These dendrimer porous layers with nanometer-sized cavity enable to achieve the inconsonant diffusion-reaction of amine molecules, and thereby obtain a uniform stripe polyamide nanofilm. Moreover, the remained amine groups in the dendrimer porous layer can participate in the IP reaction, thereby forming a polyamide nanofilm with a two-layer nanostructure via stable covalent bonds. The enhanced polarity, hydrophilic and porous, rigid dendrimer layer gives the support a higher pure water flux, which is 2.1 times than that of the original PSF support. The PSF-G4D-1 support with increased pure water flux, and the asymmetric polyamide nanofilm with highly ordered nanovoids, significantly upgrades the water permeation path and efficiency per available separation layer area. This is different from the method that decreasing the thickness of the polyamide dense layer. Thus, the resulting defect-free asymmetric polyamide membrane exhibits a desirable water flux up to 270 kg m^−2^ h^−1^ while maintains a perfect rejection of 99.1% for MgSO_4_. This work provides an idea that asymmetric structure with ordered nanovoids is to achieve highly efficient molecule/ion transport.

## Results

### Fabrication of the asymmetric polyamide nanofilm

Dendrimers are a class of highly symmetrical and regular shape molecules, a nanometer-sized cavity inside and a functional peripheral surface, which can be utilized in the classical IP process or the support modification^34,35^. The 32-amine-terminated polyamide dendrimer with dumbbell shape was synthesized by divergent method (Supplementary Figs. 1–13)^36^.

**Fig. 1:**
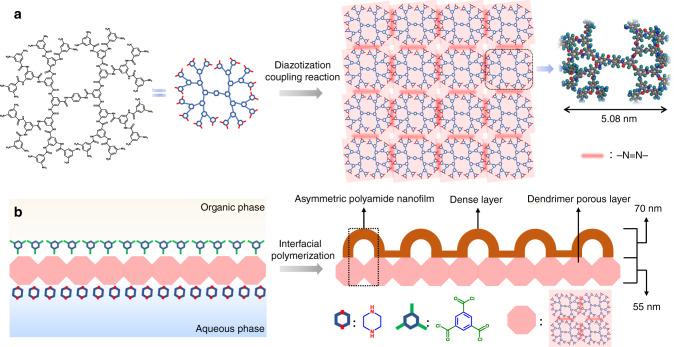
Formation of dendrimer porous layer and asymmetric polyamide nanofilm. **a** Structure of the 32-amines terminated polyamide dendrimer, and the as-prepared dendrimer porous layer assembled by diazotization-coupling reaction. **b** Schematic of asymmetric polyamide nanofilm prepared on the dendrimer porous layer.

The schematic diagram of the dendrimer porous layer assembled by diazotization-coupling reaction and the asymmetric polyamide nanofilm formed by IP process is shown in Fig. 1. A typical and optimized procedure for the fabrication of the asymmetric polyamide nanofilm was performed as follows: first, the polyamide dendrimer salt solution with pH = 1 was coated on the PSF support, and then the sodium nitrite solution was added to conduct the diazotization-coupling reaction to form dendrimer porous layer. Subsequently, a typical IP process was occurred on the amine-terminated dendrimer porous layer using the optimized piperazine (PIP) and trimesoyl chloride (TMC) formula to form the polyamide dense layer. The dendrimer porous layer is composed of nanometer-sized cavity with polarity, which is contrary to the non-polarity pores of the pristine PSF support, enable to achieve an inconsonant diffusion-reaction of PIP molecules, and thereby obtain a uniform stripe polyamide nanofilm containing highly ordered nanovoids. Moreover, the remained amine groups in the dendrimer porous layer can partly participate in the IP reaction, producing an asymmetric polyamide nanofilm with a two-layer nanostructure, namely dendrimer porous layer and polyamide dense layer, with cross-linking each other.

### Characterizations of the asymmetric polyamide nanofilm

#### Structure and morphology

**Fig. 2:**
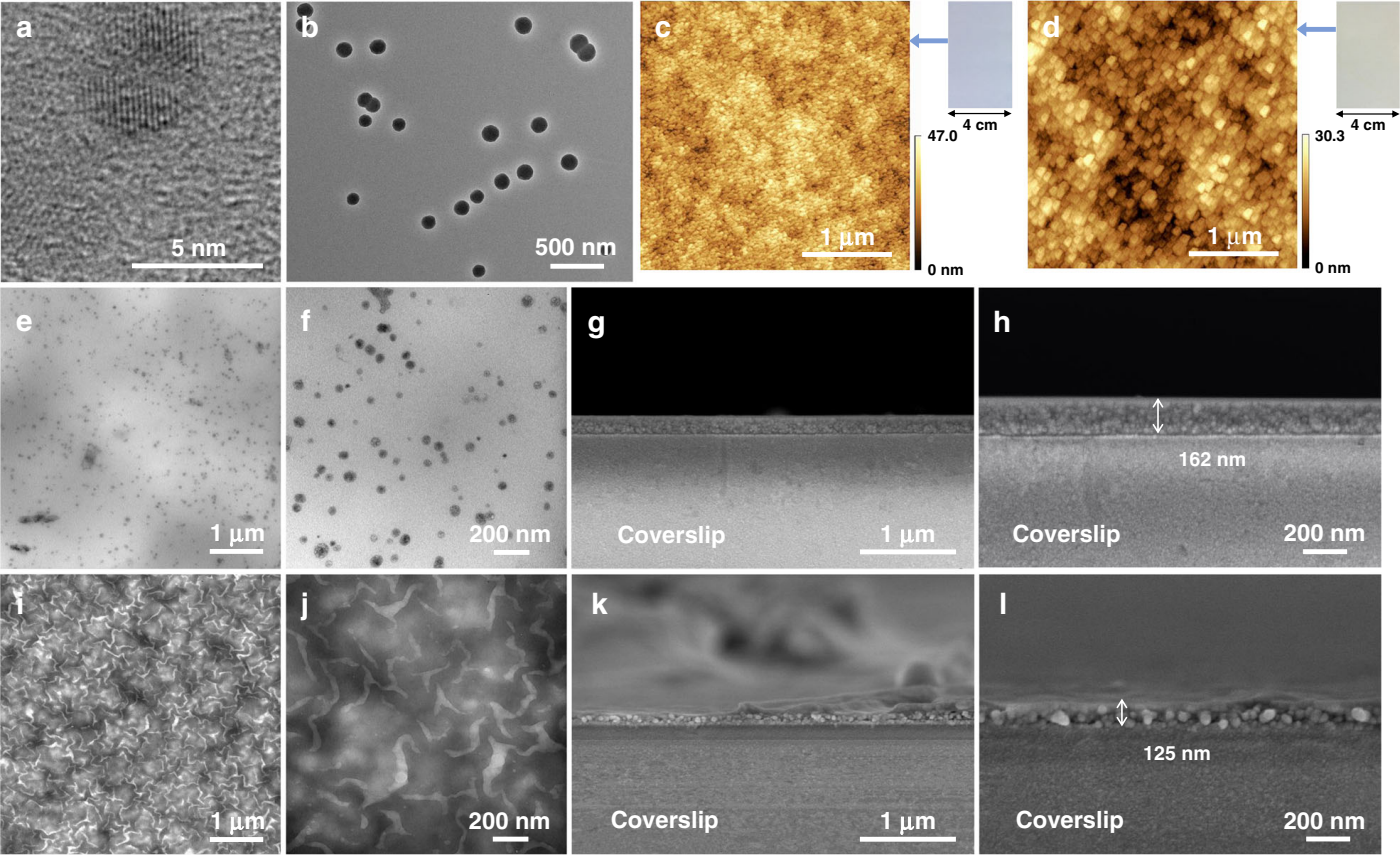
Morphology characterizations. **a** TEM image of the single polyamide G4 dendrimer (G4D). **b** TEM image of the G4D nanoparticles formed by diazotization-coupling reaction. **c** AFM image of the pristine PSF support. **d** AFM image of the polyamide dendrimer porous layer. **e**, **f** TEM images of the traditional polyamide nanofilm without dendrimer porous layer. **g**, **h** SEM cross-sectional images of the traditional polyamide nanofilm supported by coverslips. **i**, **j** TEM images of the asymmetric polyamide nanofilm. **k**, **l** SEM cross-sectional images of the asymmetric polyamide nanofilm supported by coverslips.

As shown in Fig. 2a, TEM imaging clearly shows that the single G4D exhibits a dumbbell shape, with a diameter of ~5.1 nm, which is confirmed by molecules model of energy minimization (Fig. 1a). The XRD spectra of G4D is shown in Supplementary Fig. 14, indicating a high crystallinity. Using the diazotization-coupling reaction, the single G4D enables to covalently assemble to form the spherical dendrimers nanoparticles (Supplementary Figs. 16 and 17). Figure 2b shows the TEM imaging of the covalent assembly dendrimer nanoparticle under the reaction time of 10 min, with size in the range of 98–125 nm (Supplementary Fig. 18). With an in situ covalent assembly method, we then, utilized the diazotization-coupling reaction on the PSF support with a mean effective pore diameter of 3.26 nm (see below and Supplementary Fig. 21), to form the spherical dendrimers porous layer. The SEM images shown in Supplementary Fig. 22 indicate that the PSF-G4D-1 support has a uniform and narrow pore size distribution compared to the pristine PSF support with irregular aperture size. The calculated pore size distribution of pristine PSF and PSF-G4D-1 supports also confirm this (see below). As shown in Fig. 2c, d, compared with the pristine PSF support, the dendrimer porous layer (PSF-G4D-1 support) has a uniform spherical dendrimers nanoparticle with a size of 50–60 nm.

To investigate the inner and cross-sectional morphology of the resulted IP polyamide nanofilm, we conducted the SEM and TEM measurements (Fig. 2e–l). TEM analysis clearly provides morphology information on the internal and external features of the prepared membranes. It is noted that throughout this work, we used the term traditional membrane to refer to the single-layer polyamide nanofilm prepared on the original PSF UF membrane. As shown in Fig. 2e, f, the nodule structure is evenly distributed on the surface of the traditional polyamide nanofilm, with a diameter ranged from 70 to 200 nm. The boundary of the PSF substrate and the polyamide nanofilm typically is unidentifiable, which is not conducive to study the cross-sectional morphology. Thus, for an intuitive and clear observation, we elaborately transferred the polyamide nanofilm onto the coverslips after dissolving the PSF substrate, quenched off, and fractured in liquid nitrogen for SEM analysis (Fig. 2g, h). The thickness of the traditional polyamide nanofilm in Fig. 2h is ~162 nm, showing a single-layer structure. However, the asymmetric polyamide nanofilm formed on the spherical dendrimer porous layer has a two-layer structure, as shown in Fig. 2k, l, and Supplementary Fig. 39. Specifically, the lower is a uniform spherical dendrimer porous layer with an average thickness of ~55 nm, and the upper is a nano-stripe polyamide dense layer with a thickness is ~70 nm.

Moreover, TEM images of the asymmetric polyamide nanofilm in Fig. 2i found that the nano-stripes exhibit a highly uniform and ordered nanovoids structure inside. Magnified morphology in Fig. 2j reveals that the hollow structures generally exhibit interconnected curved ribbon structure, a width of 70–120 nm, which is the same as the width of the nano-stripes in AFM imaging (see below). Besides, we investigated the volumetric mass densities of the dendrimer porous layer, traditional single polyamide nanofilm, and asymmetric polyamide nanofilm. The result found that the asymmetric polyamide nanofilm has a density value of 1.25 ± 0.08 g cm^−3^, which is in the middle (Supplementary Fig. 38). These characterizations clearly indicate that the asymmetric polyamide nanofilm having a two-layer structure exhibits a spherical dendrimer porous layer and an inner highly uniform, ordered nanovoids polyamide denser layer. Hence, compared with the traditional single-layered nodular polyamide nanofilm, the asymmetric polyamide nanofilm is effective to reduce the transmembrane resistance of water and increase the permeate flux.

### Structure and property of the modified PSF supports

**Fig. 3:**
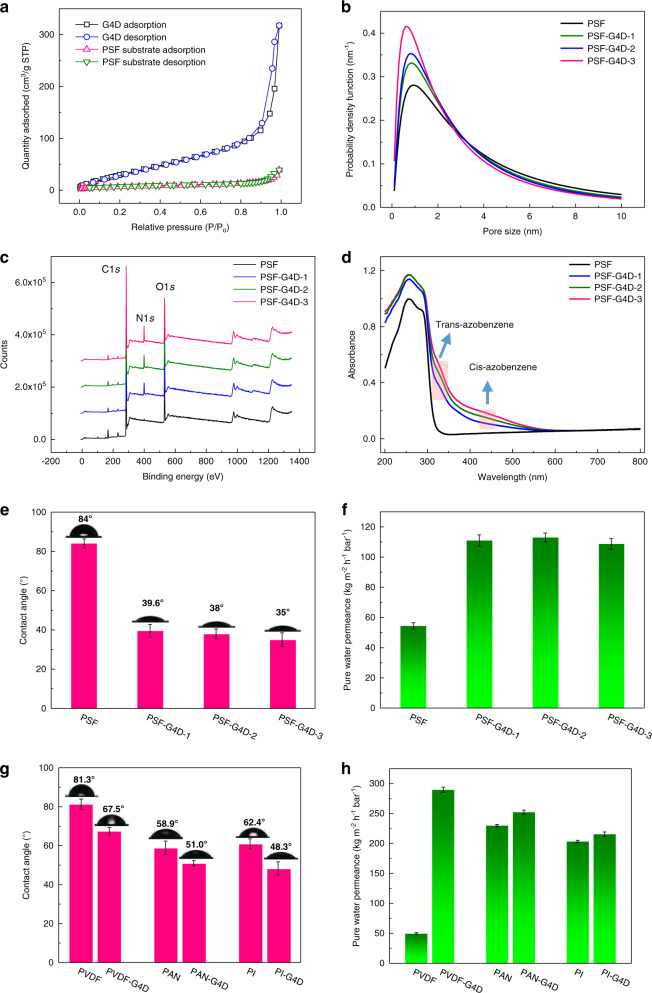
Structure characterizations and pure water permeance tests. **a** N_2_ sorption at 77 K for the G4D and the pristine PSF substrate. **b** Calculated pore size distributions averaged over the pristine PSF and the PSF-G4D membranes. **c** XPS survey spectra of the pristine PSF, PSF-G4D-1, PSF-G4D-2, and PSF-G4D-3 supports. **d** UV-visible diffuse reflectance spectrum (DRS) of the pristine PSF and the PSF-G4D-1, PSF-G4D-2, and PSF-G4D-3 supports. **e**, **f** Contact angles and pure water permeance of the pristine PSF and the PSF-G4D supports. **g**, **h** Contact angles and pure water permeance of the pristine and modified PVDF, PAN, and PI supports.

We further investigated the structure of the PSF support modified by G4D porous layer with different number of layers, and the corresponding variation in pure water permeance. As shown in Supplementary Fig. 14, such G4D dendrimers also exhibit crystallinity due to the ordered arrangement of aromatic polyamide. According to BET (Brunauer Emmet Teller) N_2_ adsorption–desorption experiments (Fig. 3a), the surface area of G4D dendrimer is as high as 140.9 m^2^ g^−1^, which is significantly higher than that of the pristine PSF support (17 m^2^ g^−1^). Moreover, after the formation of dendrimer porous layer on the PSF support, the BET surface area increases to 25.7 m^2^ g^−1^ (see below and Supplementary Fig. 20). Using the neutral-solute transport method, we calculated that the mean effective pore diameter of PSF-G4D-1 support decreases to 2.71 nm, which is lower than that of 3.26 nm for the pristine PSF support (see below and Fig. 3b)^37^. Moreover, this tendency is enhanced with the number of dendrimer porous layers. For example, in Fig. 3b, the mean effective pore diameter of PSF-G4D-3 is smaller than these of PSF-G4D-2 and PSF-G4D-1. These results are also confirmed by the surface morphology of the pristine PSF, PSF-G4D-1, PSF-G4D-2, and PSF-G4D-3 supports (Supplementary Figs. 21–24).

The XPS survey spectra in Fig. 3c reveals that the N content in PSF-G4D supports is increased with the formation of dendrimer porous layers. The UV-vis DRS of the pristine PSF and modified PSF supports in Fig. 3d further illustrate that the adsorption peak of azo double bond in the spherical dendrimers layer gradually becomes strong with the increase of dendrimer porous layer. This result confirms that the amine-dendrimers covalently attached on the PSF supports via diazotization-coupling reaction^38,39^. The mechanism that how the dendrimers were anchored covalently on pristine PSF support is presented in Supplementary Fig. 19. In addition, the ATR-FTIR spectrum of the PSF, PSF-G4D-1, PSF-G4D-2, and PSF-G4D-3 supports also confirm the formation of dendrimer porous layer (Supplementary Fig. 27). The N1*s* Scan results of the XPS spectra of the pristine PSF support and PSF-G4D-1 support in Supplementary Fig. 28 demonstrate that, due to steric hindrance, there were remained amine groups on the dendrimer porous layer that was not participated in the diazotization reaction. We rationally estimate that such remained amine groups can partially form the amide bond during IP process.

Figure 3e demonstrates that the average water contact angle (WCA) of the pristine PSF support is 84°, while that of the PSF-G4D membranes is decreased to 35°–39.6°. The wettability of the PSF-G4D supports is thus better than that of the pristine PSF support due to the introduction of hydrophilic amide groups and dendrimer porous layer. Besides, because the surface area of the G4D dendrimer is higher than that of the pristine PSF support, with intrinsic porous feature, we estimate that the rigid, porous, and hydrophilic spherical dendrimer layer can increase the water permeance of the pristine PSF support. We further investigated the influence of dendrimer porous layer numbers on water permeance of the pristine PSF support, as shown in Fig. 3f. As expected, the pure water permeance of PSF membrane increases from 54.5 to 111 kg m^−2^ h^−1^ bar^−1^, providing a twofold enhancement, and with the increase of the dendrimer porous layer number, no significant decrease in water permeance is observed.

To further confirming the utility of this strategy, PAN, PI, and PVDF ultrafiltration or microfiltration membranes were used as substrates to form the spherical dendrimers layer. As shown in Fig. 3g, h, the WCAs of the PAN, PI, and PVDF supports with dendrimer porous layers are decreased from 58.9° to 51°, 62.4° to 48.3°, and 81.3° to 67.5°, respectively, demonstrating an enhanced wettability. Furthermore, for PVDF-G4D support, a significantly enhanced water permeance is achieved, up to 289 kg m^−2^ h^−1^ bar^−1^, which is 5.8-fold higher than the pristine PVDF support. However, the pure water permeance variation of the PAN-G4D and PI-G4D supports are lower than that of the PVDF-G4D support. The changes in surface and cross-sectional morphologies of the PVDF, PAN, and PI supports after the formation of the dendrimer porous layer are shown in Supplementary Figs. 29–34. From the variation on the water permeance of the PSF, PVDF, PI, and PAN supports, this strategy may be more efficient for the polymer support with hydrophobic and porous feature.

### Surface morphology of the asymmetric polyamide membranes

**Fig. 4:**
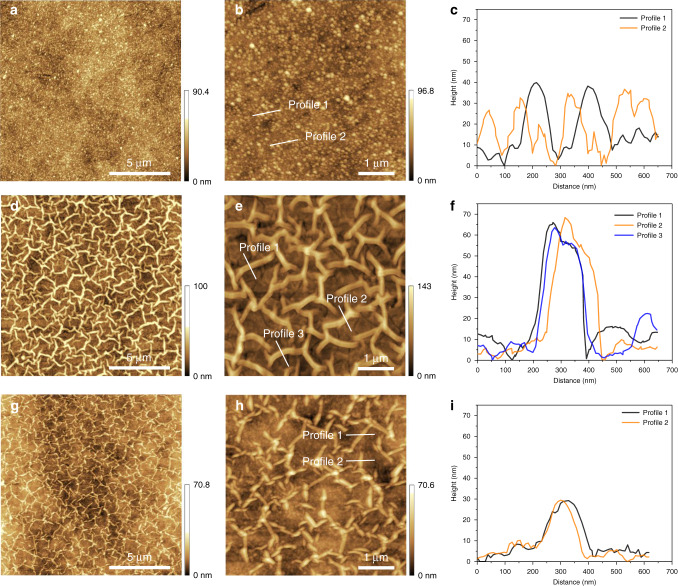
Surface morphology. **a**–**c** AFM images and corresponding height profiles of the traditional polyamide nanofilms. **d**–**f** AFM images and corresponding height profiles of the asymmetric polyamide nanofilms with 60 s IP time. **g**–**i** AFM images and corresponding height profiles of the asymmetric polyamide nanofilms with 30 s IP time.

AFM images in Fig. 4 show the surface morphology of the traditional polyamide nanofilms with nodule and the asymmetric polyamide nanofilms with nano-stripes. Here, we defined these asymmetric polyamide nanofilms with stripe surface morphology as nano-stripe or striped polyamide. Both different polyamide nanofilms exhibit a rough and heterogeneous surface structure (Supplementary Figs. 35–37). The root mean roughness (RMS) of the traditional polyamide nanofilm with nodule structure is lower than that of the asymmetric polyamide nanofilm with nano-stripe, of which the former is 8.4 nm while the latter is 18.9 nm. Moreover, the average height of the nodule structure is 36.1 nm, whereas that of the nano-stripe structure in Fig. 4fis ~65.6 nm, which is significantly higher than that of the asymmetric polyamide nanofilms with 30 s IP time. Figures 4d and g further illustrate that, the nano-stripe structure in the asymmetric polyamide nanofilms gradually becomes smaller with the decrease of the IP time, which is also confirmed by the SEM images of surface morphology in Supplementary Figs. 36 and 44. These results demonstrate that the heterogeneity of the asymmetric polyamide nanofilm with nano-stripe structure is greater than that of the nodular structure polyamide nanofilm, further confirming that the influence of the dendrimer porous layer on the morphology of the IP polyamide nanofilm is greater than that of the pristine PSF support.

### Influence of dendrimer porous layer on surface morphology

**Fig. 5:**
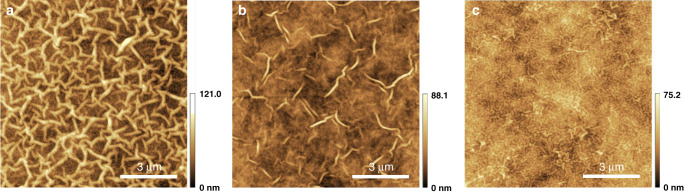
Effects of dendrimer porous layers on the morphology of asymmetric polyamide nanofilm. **a**–**c** Morphology of asymmetric polyamide nanofilm supported by PSF-G4D-1 (**a**), PSF-G4D-2 (**b**), and PSF-G4D-3 (**c**).

In order to further understand the effect of the dendrimer porous layer number on the surface morphology of asymmetric polyamide nanofilm, we conducted the IP on the PSF-G4D-1, PSF-G4D-2, and PSF-G4D-3 supports, respectively. It is noted that, as shown in Supplementary Fig. 25, with the increase of the layer number of the dendrimer porous layer on the PSF support, the size of the dendrimer nanoparticles formed by diazotization-coupling reaction gradually decrease. AFM images in Fig. 5 show that the nano-stripes of the asymmetric polyamide nanofilm gradually decrease until disappearing.

It is well known that the support characteristic such as pore size, uniformity, wettability, and morphology, can affect the amine molecule distribution, further vary the diffusion-reaction rate, and then result in the formation of a heterogeneous polyamide nanofilm^40–42^. In this work, we further discussed the influence of solid surface energy and pore size on the polyamide nanofilm morphology. Typically, solid surface energy can be divided into polar part ($\gamma _{\mathrm{s}}^{\mathrm{p}}$) and non-polar part ($\gamma _{\mathrm{s}}^{\mathrm{d}}$)^43^. As shown in Table 1, for the pristine PSF support, the solid surface energy mainly presents the non-polar part ($\gamma _{\mathrm{s}}^{\mathrm{d}}$), and a value of 46.2 mJ m^−2^. This is because that the pore of the pristine PSF support is mainly derived from the phase inversion of casting solution, and thus shows a non-polar property. With the increase of the number of dendrimer porous layers, the polar part ($\gamma _{\mathrm{s}}^{\mathrm{p}}$) increases, while the non-polar part ($\gamma _{\mathrm{s}}^{\mathrm{d}}$) decreases. For example, the $\gamma _{\mathrm{s}}^{\mathrm{p}}$ of the PSF-G4D-1 increases to 24.8 mJ m^−2^, which is higher than that of the pristine PSF support (1.42 mJ m^−2^). In addition, the pore sizes of the PSF-G4D supports were also reduced accordingly with the increase of the number of dendrimer porous layers. These pristine supports with larger mean effective pore size (*r*_p_ = 3.26 nm) and non-polar surface properties, mainly forms the traditional polyamide nanofilm with nodule structure (Fig. 4a, b, Supplementary Fig. 35) during IP process. For the PSF-G4D-1 support, compared with the pristine PSF support, the pore size decreases from 3.26 to 2.71 nm, polar part of solid surface energy increases from 1.42 to 24.8 mJ m^−2^, while that of the non-polar part decreases from 46.2 to 38.4 mJ m^−2^.

**Table 1:** Solid surface energy and mean effective pore size.

Membrane type	Contact angle (°)		Solid surface energy (mJ m^−2^)			Mean effective pore size (nm)
	Water	Diiodomethane	$\gamma _{\mathrm{s}}^{\mathrm{d}}$	$\gamma _{\mathrm{s}}^{\mathrm{p}}$	*γ*_s_	*r*_p_
Pristine PSF support	84.0	25.0	46.2	1.42	47.6	3.26
PSF-G4D-1	39.6	42.4	38.4	24.8	63.2	2.71
PSF-G4D-2	38.0	51.4	33.5	28.4	61.9	2.54
PSF-G4D-3	35.0	55.3	31.3	31.5	62.8	2.23

Contact angle and the calculated solid surface energy $\gamma _{\mathrm{s}}$, non-polar ($\gamma _{\mathrm{s}}^{\mathrm{d}}$) and polar part ($\gamma _{\mathrm{s}}^{\mathrm{p}}$) surface energy, and the mean effective pore size ($r_{\mathrm{p}}$) of the pristine PSF support and PSF-G4D-1, PSF-G4D-2, and PSF-G4D-3 supports.

Based on the above discussion, the PSF-G4D-1 support enable to provide an unusual amine solutions distribution, make an inconsonant diffusion-reaction rate, thus form a nano-striped polyamide layer with highly ordered nanoscale voids. However, with the continuous increase in the number of dendrimer porous layers, the ratio of the non-polar part and the polar part of the solid surface energy gradually equals, and the pore size continues to decrease. The amine molecules distribution on the PSF-G4D-2 and PSF-G4D-3 supports gradually tends to be uniform, and thus the asymmetric polyamide nanofilm with nano-stripe structure thus became rare or even disappears.

### Separation performance of the asymmetric polyamide membranes

We evaluated the separation properties of the resulted polyamide nanofilms, and further explored the relationship between structure and separation performance, as shown in Table 2. Separation performance measurements were conducted 1 h after starting the filtration to stabilize the membrane performance. Rejection rate was generally calculated on the basis of the electrical conductivities of feed and permeation solutions. Separation data show that the asymmetric polyamide membrane was improved in both flux and rejection compared to the traditional polyamide membrane. The asymmetric polyamide membrane provides a 3.7−4.3-folds higher water flux for various salt solutions with 2000 ppm compared with the traditional polyamide membrane. Moreover, the salt rejection rate has also been improved. For example, MgSO_4_ rejection rate of the asymmetric polyamide membrane was increased from 93.4 to 99.1%, and water flux was increased from 68.5 to 270 kg m^−2^ h^−1^.

**Table 2:** Separation performance.

Salt	Traditional polyamide membrane		Asymmetric polyamide membrane	
	Water flux (kg m^−2^ h^−1^)	Rejection (%)	Water flux (kg m^−2^ h^−1^)	Rejection (%)
Na_2_SO_4_	71.4 ± 2.4	96.0 ± 1.8	264 ± 5.1	99.2 ± 0.3
MgSO_4_	68.5 ± 2.8	93.4 ± 1.6	270 ± 8.3	99.1 ± 0.3
CaCl_2_	59.3 ± 3.1	77.1 ± 1.4	236 ± 7.4	77.0 ± 1.8
MgCl_2_	61.9 ± 2.9	80.2 ± 2.1	259 ± 6.3	80.0 ± 1.5
NaCl	71.4 ± 3.3	33.7 ± 2.3	306 ± 9.7	41.1 ± 1.4

Separation performance of the traditional polyamide nanofilm prepared on the pristine PSF substrate, and the asymmetric polyamide nanofilm prepared on the PSF-G4D-1 substrate, reaction time: 60 s. The operating pressure and temperature were controlled at 1 MPa, 25 °C. The feed flow rate was 7.5 L min^–1^, and the salt concentrations in the feed solutions were 2000 ppm.

Based on the SEM and TEM analysis (see below, Supplementary Figs. 39 and 40), the enhancement in water flux of the asymmetric polyamide membrane is mainly attributed to the increased pure water flux permeance of the PSF-G4D-1 support, the decrease in the thickness of the polyamide dense layer, and the formation of the uniform nano-stripes containing highly ordered nanovoids structure. Generally, the highly uniform, ordered hollow nano-stripes structure inside the polyamide structure can increase the water-permeable area of unit nanofilm area, thereby improving the water transmission efficiency. The thinner polyamide dense layer aims to lower the resistance of water transport across the active layer, and thereby improve permeance. In addition, the enhancement in water permeability of the support facilitates the transmission of permeation side water of the dense polyamide layer.

For salt rejection, since the pristine PSF support exhibits a larger pore size and uneven pore distribution, it is difficult to rapidly form a defect-free polyamide nanofilm when the amine molecule diffuses from the aqueous phase to the organic phase (Supplementary Fig. 21). The atomic composition assessed by XPS measurements in Supplementary Table 4 shows that the cross-linking degree of the asymmetric polyamide nanofilm (63.2%) is higher than that of the traditional polyamide nanofilm (32.6%), which is conducive to enhance the salt rejection. The pore size distribution curves in Supplementary Fig. 48 show that the asymmetric polyamide nanofilm exhibits a decreased mean effective pore radius (0.19 nm) than that of the traditional nanofilm (0.22 nm). These results indicate that the asymmetric polyamide nanofilm exhibits a more efficient size exclusion effect on the ions to be separated, and high salt rejection rate thus can be achieved. In addition, the surface zeta potential values of the asymmetric and traditional polyamide nanofilms are both negative (Supplementary Fig. 42), which is contributed to enhance the charge exclusion effect for SO_4_^2−^ with a valence of −2, and thus increase salt rejection rate.

### Permeability−selectivity of asymmetric polyamide membrane

**Fig. 6:**
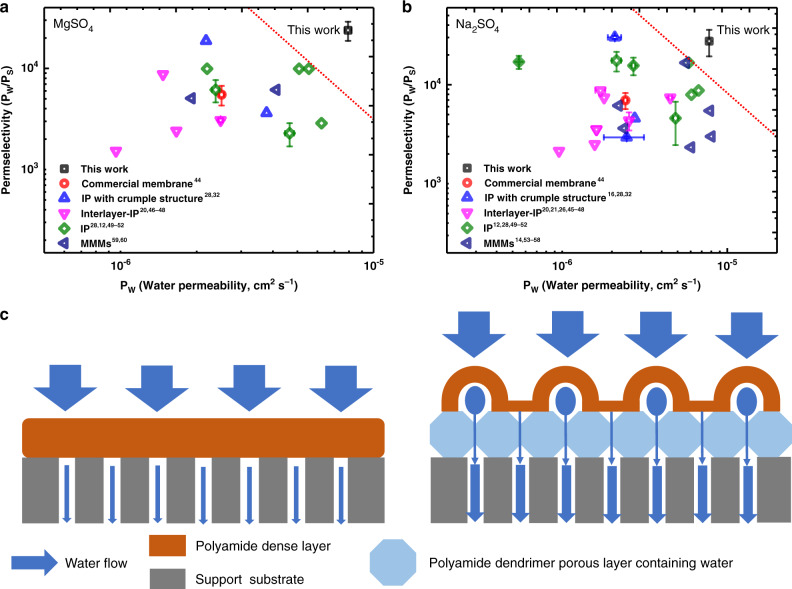
Water permeability and water−salt selectivity. **a**, **b** Correlation between water permeability and water−salt selectivity for the asymmetric polyamide membrane and the commercial as well as reported other types of polyamide membranes in literatures. The asymmetric polyamide membrane was prepared on the PSF-G4D-1 substrate and the reaction time was 60 s. These data were all obtained from corresponding MgSO_4_ or Na_2_SO_4_ experiments. The dashed red line is the permeability−selectivity trade-off (upper-bound line) for current different types of polyamide membranes, such as commercial polyamide membrane (Commercial membrane), polyamide membrane prepared by interlayer (Interlayer-IP), crumpled polyamide membrane (IP with crumpled structure), mixed matrix polyamide membranes (MMMs), and traditional IP membrane (IP). **c** Schematic diagrams of the transport path of water across the traditional and asymmetric polyamide membranes.

Trade-off between permeability and selectivity is found for all types of membranes. Upper-bound line represents the level of permeability and selectivity achieved by the currently prepared membranes. In order to illustrate the superiority of the asymmetric polyamide nanofilm between permeability and selectivity, we calculated related permeability/selectivity data of different types of polyamide membranes as currently reported in the literature, such as commercial polyamide membrane (Commercial membrane)^44^, crumpled polyamide membrane (IP with crumpled structure)^16,28,32^, polyamide membrane prepared by interlayer (Interlayer-IP)^20,21,26,45–48^, traditional IP membrane (IP)^12,28,49–52^, and mixed matrix polyamide membranes (MMMs)^14,53–60^, as shown in Fig. 6a, b. It should be noted that we used the thickness of the monolayer polyamide dense layer (70 nm) in asymmetric nanofilm to calculate the permeability.

From Fig. 6a, b, it was observed that the water permeability and permselectivity for Na_2_SO_4_ range from 5.47 × 10^−7^ ± 3.85 × 10^−9^ cm^2^ s^−1^ (from IP) to 7.85 × 10^−6^ cm^2^ s^−1^ (from MMMs), 2139.1 (from Interlayer-IP) to ${\mathrm{30080}}{\mathrm{.6 \pm 1077}}{\mathrm{.3}}\,{\mathrm{(from}}\, {\mathrm{IP}}\,{\mathrm{with}}\, {\mathrm{crumpled}}\,{\mathrm{structure)}}$, respectively. The water permeability and permselectivity for MgSO_4_ ranges from 9.6 × 10^−7^ cm^2^ s^−1^ (from Interlayer-IP) to 6.23 × 10^−6^ cm^2^ s^−1^ (from IP), 1528.4 (from Interlayer-IP) to 18743.7 (from IP), respectively. These results demonstrate that the current nanofilm structures are difficult to achieve a balance between permeability and selectivity, and break the upper-bound line. However, for the asymmetric polyamide nanofilm with highly uniform, ordered hollow nano-stripe structure supported by dendrimer porous layer, the water permeability and permselectivity values for Na_2_SO_4_, MgSO_4_, are ${\mathrm{7}}{\mathrm{.8 \times 10}}^{{\mathrm{ - 6}}}{\mathrm{ \pm 1}}{\mathrm{.4 \times 10}}^{{\mathrm{ - 7}}}\,{\mathrm{cm}}^2\,{\mathrm{s}}^{{\mathrm{ - 1}}}$ and ${\mathrm{27561}}{\mathrm{.3 \pm 8245}}$, ${\mathrm{7}}{\mathrm{.9 \times 10}}^{{\mathrm{ - 6}}}{\mathrm{ \pm 2}}{\mathrm{.4 \times 10}}^{{\mathrm{ - 7}}}\,{\mathrm{cm}}^2\,{\mathrm{s}}^{{\mathrm{ - 1}}}$ and $23877.2 \pm 5133$, respectively, which has overcome the upper-bound line of the other polyamide membranes, demonstrating a better trade-off. As illustrated in Fig. 6c, benefiting from the hydrophilic dendrimer porous layer, and thinner polyamide dense layer with highly uniform, ordered hollow nano-stripes structure, the asymmetric polyamide nanofilm exhibit the transport path of water across that different from the traditional polyamide nanofilm. And thereby a significant enhancement in water permeation efficiency. Specifically, the thinner polyamide dense layer can reduce the transmembrane resistance of water transport across the active layer. The ordered nanovoids in the hollow nanostrips can enhance the water permeance per unit area. And the dendrimer porous layer containing water serves as transmission channel to increase transmission efficiency of water.

### Stability

**Fig. 7:**
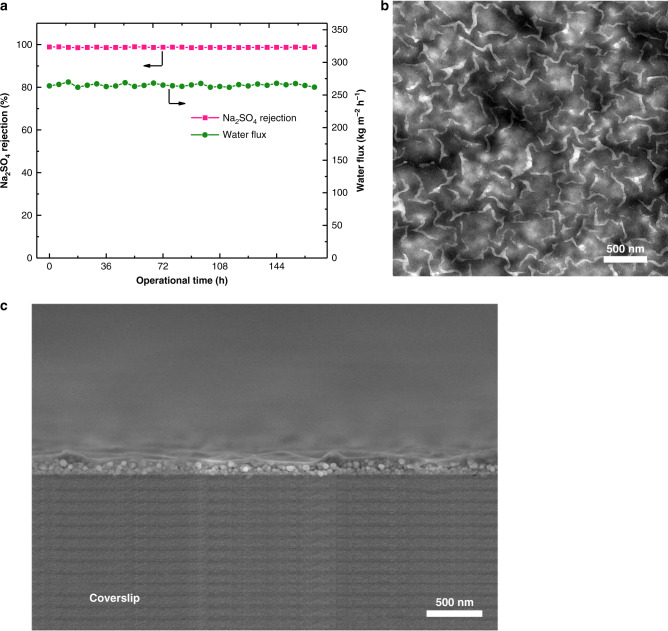
Operational stability. **a** Operational stability of the asymmetric polyamide membrane. **b** TEM image of the asymmetric polyamide nanofilm. **c** SEM cross-section image of the asymmetric polyamide nanofilm. The asymmetric polyamide nanofilm was prepared on the PSF-G4D-1 substrate, reaction time: 60 s.

Operational stability is one of the key properties for the asymmetric polyamide membrane, especially for that with uniform nano-stripes structure containing highly ordered nanovoids. We thus conducted the long-term stability test for this structure polyamide membrane. The experiment reveals that the asymmetric polyamide membrane exhibits a good operational stability at 168 h cross-flow test under 1 MPa for 2000 ppm Na_2_SO_4_, of which salt rejection remains at more than 99% and water flux ranges from 262 to 270 kg m^−2^ h^−1^. As shown in Fig. 7b, c, we attest that the rigid, uniform spherical dendrimer porous layer provides a long-term sustainability for the highly ordered hollow nano-stripes structure polyamide nanofilm, demonstrating that is an efficient strategy to design high permeability−selectivity polyamide membrane.

## Discussion

In summary, a uniform spherical polyamide dendrimer porous layer was covalently assembled on the PSF support by diazotization-coupling reaction, and then a dense polyamide nanofilm was formed on the above dendrimer porous layer via the IP process. We have demonstrated that the asymmetric polyamide nanofilm was successfully fabricated, having a two-layer structure, the lower was a spherical polyamide dendrimer porous layer, and the upper was the dense polyamide layer with a highly uniform nano-striped structure containing ordered nanovoids. The asymmetric polyamide membranes exhibit good performance in water purification, where they are both more permeable and more selective than the commercially available and previously reported polyamide membranes, showing a high and stable permeability−selectivity upper-bound line. In addition, the UF support covalently assembled by spherical polyamide dendrimer porous layer exhibits a significant increase in pure water flux, which is also demonstrated on the PVDF, PAN, and PI support. The dendrimers with different structures or functional groups can be applied in the modification of UF or MF membrane via covalent assembly, and then the separation nanofilm having a difference in layer structure is prepared by IP or coating or grafting method. This work might inspire the structural design of two-layer or multi-layer nanofilm for separation, to break through the existing upper-bound line of the permeability−selectivity performance of various membranes with great potential for applications in molecular separations, including organic solvent nanofiltration, seawater desalination, water purification, and pervaporation, etc.

## Methods

### Preparation of dendrimer porous layer on PSF support

G4 dendrimer dissolved in NMP (5 mL) was added dropwise to a hydrochloric acid solution of pH = 1 under stirring at room temperature to form a 0.24 mM salt solution. The PSF UF membrane was soaked for 30 min in the above salt solution, and the excess solution was subsequently removed by an air knife. Afterwards, the sodium nitrite solution (72.5 mM, pH = 1, 0 °C) was poured on the above PSF substrate to conduct the covalent assembly, thereby forming a polyamide dendrimer porous layer. We labeled the above-modified PSF substrate as PSF-G4D-1. Similarly, we repeated the diazotization-coupling reaction process on the PSF-G4D-1 substrate to fabricate the PSF-G4D-2, and modified the PSF-G4D-2 substrate to fabricate the PSF-G4D-3. We further modified the pristine PVDF, PAN, and PI supports to fabricated the PVDF-G4D, PAN-G4D, and PI-G4D substrates in a similar way. The advantage of polyamide dendrimer porous layer is that this thin layer can overcome the aperture defect (inhomogeneity) of the underlying PSF support, and produces a more hydrophilic surface for IP required, to form the defect-free nanofilms at the interface.

### Preparation of asymmetric polyamide nanofilms

IP is a well-known technique used to form the polyamide dense layer on a support membrane. Specifically, the PSF, PSF-G4D-1, PSF-G4D-2, and PSF-G4D-3 substrates were first immersed into PIP solution (1 w/v%) for 10 min. Subsequently, the extra amine solution on the support surface was blown off with an air knife. Then, the above support was contacted with TMC/n-hexane solution (0.15 w/v%) for 30 or 60 s to form the traditional or asymmetric polyamide nanofilm. And the organic phase solution on the surface was instantly blown off with an air knife. The resulted polyamide membranes were finally dried at 60 °C for 2 min and stored in DI water until use.

### Membrane performance test

For the pristine and modified supports, the pure water flux measurements were made 1 h after starting the filtration to stabilize the membrane performance. The operating pressure and temperature were controlled at 0.2 MPa, 25 °C, and the feed cross-flow rate was 7.5 L min^−1^. For the IP polyamide membranes, separation performance was determined with different salt solutions in a cross-flow system, with an effective test area (*A*) of 19.3 cm^2^. The salt concentration, such as MgCl_2_, CaCl_2_, MgSO_4_, Na_2_SO_4_, and NaCl, in the feed solution was 2 g L^−1^. The separation performance tests were conducted at an operating pressure of 1 MPa and a temperature of 25 °C. The performance data were determined after the water flux and conductivity reached a steady state. For the stability test, the asymmetric polyamide membrane was continuously operated for 168 h at 1 MPa, 25 °C. The water flux (kg m^−2^ h^−1^) was calculated from the weight of the permeate (*M*) for a specified time, as given by the following equation:

1 $${\mathrm{Water}}\,{\mathrm{flux}}\,({\mathrm{kg}}\,{\mathrm{m}}^{{\mathrm{ - 2}}}\,{\mathrm{h}}^{{\mathrm{ - 1}}}) = M/At$$

The salt rejection was determined from the conductivity of the feed solution (*C*_f_) and the permeate (*C*_p_). Hence, the salt/ion rejection can be calculated from the following equation:

2 $${\mathrm{Rejection}}\,\left( {\mathrm{\% }} \right)\,{\mathrm{ = }}\,\left( {1 - C_{\mathrm{p}}/C_{\mathrm{f}}} \right){\mathrm{ \times 100\% }}$$

Based on the solution diffusion model, the water permeability of dense polyamide nanofilm is related to the water flux as defined by the following equation^22,23^:

3 $$P_{\mathrm{w}} = \frac{{J_{\mathrm{w}}}}{{\left( {{\Delta} p - {\Delta} {\uppi}} \right)}}\frac{{{\mathrm{RT}}h}}{{v_{\mathrm{w}}}}$$

4 $${\Delta} \uppi = c{\mathrm{RT}}$$

The permeability coefficient of a polyamide nanofilm is defined as the water flux (*J*_w_, L m^−2^ h^−1^). Here, we define the water flux unit, kg m^−2^ h^−1^, is equal to the L m^−2^ h^−1^. The selected dense polyamide layer thickness (*h*, nm), the relative molar volume of water (*v*_w_), the driving force (Δ*p*) required to overcome the osmotic pressure (Δπ) of feed solution, the normalized thermodynamic constant (RT), and *c* is the ion molar concentration (mol L^−1^).

Of which

$$R 	= \, 8.314 \times\! {\mathrm{10}}^6\,{\mathrm{cm}}^3 \cdot {\mathrm{Pa}}\,{\mathrm{K}}^{{\mathrm{ - 1}}}{\mathrm{mol}}^{{\mathrm{ - 1}}}\\ T 	= \, 298\,{\mathrm{K}}\\ v_{\mathrm{w}} 	= \, {\mathrm{18}}{\mathrm{.04}}\,{\mathrm{cm}}^3\,{\mathrm{mol}}^{ - 1}$$

The salt permeability of the polyamide nanofiltration membrane is related to the salt flux, $J_{\mathrm{s}} = P_{\mathrm{s}}{\Delta} C_{\mathrm{s}}/h$. On the other hand, the salt permeability also can be measured by the salt water flux $(J_{\mathrm{w}}{\mathrm{,}}\,{\mathrm{L}}\,{\mathrm{m}} ^{- 2}\,{\mathrm{h}} ^{- 1})$ and salt rejection rate (*R*_s_, %), $J_{\mathrm{s}} = J_{\mathrm{w}}{\Delta} C_{\mathrm{s}}\left( {\left( {{\mathrm{100\% }}/R_{\mathrm{s}}} \right) - 1} \right)$. Hence, based on the above equations, we can acquire the salt permeability equation:

5 $$P_{\mathrm{s}} = J_{\mathrm{w}}h\left( {\frac{{{\mathrm{100\% }}}}{{R_{\mathrm{s}}}} - 1} \right)$$

Finally, permselectivity (*α*) is a dimensionless quantity that is defined as the ratio of the water permeability to the salt permeability:

6 $$\alpha = \frac{{P_{\mathrm{w}}}}{{P_{\mathrm{s}}}} = \left( {\frac{{R_{\mathrm{s}}}}{{100\% - R_{\mathrm{s}}}}} \right)\frac{{{\mathrm{RT}}}}{{\left( {{\Delta} p - {\Delta} \uppi } \right)v_{\mathrm{w}}}}$$

The following is the calculation steps of solid surface energy:

It is known that solid surface energy can be divided into polar part $(\gamma _{\mathrm{S}}^{\mathrm{p}})$ and non-polar part $(\gamma _{\mathrm{S}}^{\mathrm{d}})$. That is,

7 $$\gamma _{\mathrm{S}} = \gamma _{\mathrm{S}}^{\mathrm{d}} + \gamma _{\mathrm{S}}^{\mathrm{p}}$$

Based on the modified Eq. (8) provided by Owen and Wendt^43^:

8 $$\gamma _{{\mathrm{SL}}} = {{r}}_{\mathrm{s}} + {{r}}_{\mathrm{L}} - {\mathrm{2}}\left( {\gamma _{\mathrm{S}}^{\mathrm{d}}\gamma _{\mathrm{L}}^{\mathrm{d}}} \right)^{{\mathrm{1/2}}} - {\mathrm{2}}\left( {\gamma _{\mathrm{S}}^{\mathrm{p}}\gamma _{\mathrm{L}}^{\mathrm{p}}} \right)^{{\mathrm{1/2}}}$$

Combined the well-known Young equation, we can get that following equation:

9 $$\left( {{\mathrm{1 + cos}}\theta } \right)\gamma _{\mathrm{L}} = {\mathrm{2}}\left( {\gamma _{\mathrm{S}}^{\mathrm{d}}\gamma _{\mathrm{L}}^{\mathrm{d}}} \right)^{{\mathrm{1/2}}} + {\mathrm{2}}\left( {\gamma _{\mathrm{S}}^{\mathrm{p}}\gamma _{\mathrm{L}}^{\mathrm{p}}} \right)^{{\mathrm{1/2}}}$$

Where *γ*_SL_ is the free energy of the interface between liquid and solid, $\gamma _{\mathrm{L}}^{\mathrm{d}}$ and $\gamma _{\mathrm{L}}^{\mathrm{p}}$ are the non-polar part and polar part of the liquid surface energy (*γ*_L_), respectively.

From Eq. (9), assuming that the contact angles of the two liquids with known surface energy on the solid surface were *θ*_1_ and *θ*_2_, respectively. Then, we can obtain the following equation:

10 $$\left( {{\mathrm{1 + cos}}\theta _{\mathrm{1}}} \right)\gamma _{{\mathrm{L1}}} = {\mathrm{2}}\sqrt {\gamma _{\mathrm{S}}^{\mathrm{d}}\gamma _{{\mathrm{L1}}}^{\mathrm{d}}} + {\mathrm{ 2}}\sqrt {\gamma _{\mathrm{S}}^{\mathrm{p}}\gamma _{{\mathrm{L1}}}^{\mathrm{p}}}$$

11 $$\left( {{\mathrm{1}} + {\mathrm{cos}}\theta _{\mathrm{2}}} \right)\gamma _{{\mathrm{L2}}} = {\mathrm{2}}\sqrt {\gamma _{\mathrm{S}}^{\mathrm{d}}\gamma _{{\mathrm{L2}}}^{\mathrm{d}}} + {\mathrm{2}}\sqrt {\gamma _{\mathrm{S}}^{\mathrm{p}}\gamma _{{\mathrm{L2}}}^{\mathrm{p}}}$$

Based on the Eqs. (10) and (11), the following equation can be obtained:

12 $$\sqrt {\gamma _{\mathrm{S}}^{\mathrm{d}}} = \frac{{\left( {{\mathrm{1 + cos}}\theta _{\mathrm{2}}} \right){\upgamma}_{{\mathrm{L2}}}\sqrt {\gamma _{{\mathrm{L1}}}^{\mathrm{p}}} -\left( {{\mathrm{1}} + {\mathrm{cos}}\theta _{\mathrm{1}}} \right)\gamma _{{\mathrm{L1}}}\sqrt {\gamma _{{\mathrm{L2}}}^{\mathrm{p}}} }}{{{\mathrm{2}}\sqrt {\gamma _{{\mathrm{L2}}}^{\mathrm{d}}\gamma _{{\mathrm{L1}}}^{\mathrm{p}}} - {\mathrm{2}}\sqrt {\gamma _{{\mathrm{L1}}}^{\mathrm{d}}\gamma _{{\mathrm{L2}}}^{\mathrm{p}}} }}$$

13 $$\sqrt {\gamma _{\mathrm{S}}^{\mathrm{p}}} = \frac{{\left( {{\mathrm{1}} + {\mathrm{cos}}\theta _{\mathrm{2}}} \right)\gamma _{{\mathrm{L2}}}\sqrt {\gamma _{{\mathrm{L1}}}^{\mathrm{d}}} - \left( {{\mathrm{1}} + {\mathrm{cos}}\theta _{\mathrm{1}}} \right)\gamma _{{\mathrm{L1}}}\sqrt {\gamma _{{\mathrm{L2}}}^{\mathrm{d}}} }}{{{\mathrm{2}}\sqrt {\gamma _{{\mathrm{L2}}}^{\mathrm{p}}\gamma _{{\mathrm{L1}}}^{\mathrm{d}}} - {\mathrm{2}}\sqrt {\gamma _{{\mathrm{L1}}}^{\mathrm{p}}\gamma _{{\mathrm{L2}}}^{\mathrm{d}}} }}$$

Combined the Eqs. (12) and (13), $\gamma _{\mathrm{S}}^{\mathrm{d}}$ and $\gamma _{\mathrm{S}}^{\mathrm{p}}$ can be obtained. Bringing these two parameters into the Eq. (7), the solid surface energy (*γ*_S_) was finally calculated.

The following is the methods of mean effective pore size and pore size distribution:

The variation of molecular weight cut-off (MWCO), mean effective pore size and pore size distribution of the pristine and modified PSF support, IP polyamide membrane, were characterized by means of the solute transport method^37^. 0.2 g L^−1^ polyethylene glycol (PEGs) with different *M*_w_ including 200, 400, 600, 800, 1000, 10,000, 20,000, 70,000, 100,000, and 300,000 Da were used to measure the molecular weight cut-off (MWCO) and solution rejection (*R*_T_) of the resulted membrane. The solution rejection (*R*_T_) can be obtained according to the following equation:

14 $$R_{\mathrm{T}}=\left( {{\mathrm{1 - }}\frac{{C_{\mathrm{p}}}}{{C_{\mathrm{f}}}}} \right){\mathrm{ \times 100\% }}$$

Where *C*_p_ is the total organic carbon (TOC) of permeation solution, *C*_f_ is the TOC of feed solution.

The relationship between Stokes radius *r*_s_ and molecular weight, *M*_w_, of these PEGs solutes can be expressed by the following equation:

15 $$r_{\mathrm{s}}\left( {{\mathrm{nm}}} \right) = 16{\mathrm{.74 \times 10}}^{{\mathrm{ - 3}}} \times M_{\mathrm{w}}^{{\mathrm{0}}{\mathrm{.557}}}$$

Where *r*_s_ is nm and *M*_w_ is g mol^−1^, respectively. From this equation, the radius of a hypothetical solute at a given *M*_w_ can be obtained. The MWCO, which is referred to as the molecular weight that above 90% of the solute in the feed solution is retained by the membrane.

By plotting the solute rejection against the PEG Stokes diameter in a log-normal probability plot, a straight line and a linear equation would be obtained through curve fitting. From this equation, the mean solute size (*µ*_s_) could be obtained when *R*_T_ was 50%, and *σ*_g_ (geometric standard deviation of *µ*_s_) was determined from the ratio of *d*_s_ at *R*_T_ = 84.13 and 50%. By ignoring the steric and hydrodynamic interaction between the solute and the pore size, the mean pore size (*µ*_p_) and the geometric standard deviation (*σ*_p_) were the same as *µ*_s_ and *σ*_g_, respectively. Hence, the pore size distribution could be expressed by the following probability density function:

16 $$\frac{{{\mathrm{d}}R_{\mathrm{T}}\left( {r_{\mathrm{p}}} \right)}}{{{\mathrm{d}}r_{\mathrm{p}}}} = \frac{{\mathrm{1}}}{{r_{\mathrm{p}}{\mathrm{ln}}\,\sigma _{\mathrm{p}}\sqrt {{\mathrm{2}}\pi } }}{\mathrm{exp}} - \frac{{ {\left({\mathrm{ln}}\,r_{\mathrm{p}} - {\mathrm{ln}}\, \mu _{\mathrm{p}} \right)^{\mathrm{2}}}}}{{{\mathrm{2}}\left( {{\mathrm{ln}}\,\sigma _{\mathrm{p}}} \right)^{\mathrm{2}}}}$$

## Supplementary information

Supplementary Information

## Data Availability

The authors declare that all of the data supporting the finding of this study are available within the paper and supplementary information and also are available from the corresponding author upon reasonable request. Source data are provided with this paper.

## Source data

Source Data

## Code availability

All codes written for and used in this study are available from the corresponding authors upon reasonable request.
