# Supplementary material for: Asymmetric polyamide nanofilms with highly ordered nanovoids for water purification
Source: Nat Commun. 2020 Nov 30;11:6102. doi: 10.1038/s41467-020-19809-3 (PMC7705655; doi:10.1038/s41467-020-19809-3)
Supplement: Supplementary file 1 — Supplementary Information [file 41467_2020_19809_MOESM1_ESM.pdf]

# Asymmetric polyamide nanofilms with highly ordered nanovoids for water purification

Bingbing Yuan[1], Shengchao Zhao[2,3], Ping Hu[1], Jiabao Cui[1] & Q. Jason Niu[2,3]

[1]School of Chemistry and Chemical Engineering, Key Laboratory of Green Chemical Media and Reactions Ministry of Education, Henan Normal University, 453007 Xinxiang, China. [2]Institute for Advanced Study, Shenzhen University, 518060 Shenzhen, China. [3]State Key Laboratory of Heavy Oil Processing, College of Chemical Engineering, China University of Petroleum (East China), 266555 Qingdao, China. Correspondence and requests for materials should be addressed to B. Y. (email: yuanbingbing@htu.edu.cn) or to Q. J. N. (email: qjasonniu@szu.edu.cn)

**List of Content:**

**Supplementary Methods**

**Supplementary Text**

**Supplementary Figures 1 to 51**

**Supplementary Tables 1 to 12**

**References**

## 1. Supplementary Methods

### 1.1. Chemicals and materials

Trimesoyl chloride (TMC), piperazine (PIP), m-Phenylenediamine, trifluoroacetic anhydride, 3,5-diaminobenzoic acid, sodium nitrite, P-phenylenediamine and hydrazine hydrate were purchased from Tokyo Chemical Industry (Japan). Sodium hydroxide (NaOH), sodium chloride (NaCl), magnesium chloride hexahydrate ($MgCl_2 \cdot 6H_2O$), anhydrous magnesium sulfate ($MgSO_4$), sodium sulfate ($Na_2SO_4$), calcium chloride ($CaCl_2$), sodium hydrogen carbonate, N-Methyl-2-pyrrolidinone (NMP), thionyl chloride, tetrachloroethane, dichloromethane, ethyl acetate, acetonitrile, cyclohexane, n-hexane, hydrochloric acid, triethylamine (TEA), camphor sulfonic acid (CSA), polyvinyl pyrrolidone (PVP) K60, polyethylene glycol (PEG), methanol and N,N-Dimethylformamide (DMF) were obtained from Sinopharm Chemical Reagent Co., Ltd. and used without further treatment. Deionized (DI) water (0.5−1.5 μs $cm^{-1}$) was prepared in a two-stage reverse osmosis purification system. P84 polyimide (PI) was purchased from HP Polymer Gmbh (Austria). Polyvinylidene fluoride (PVDF 6015/1001) was purchased from Solvay. Polysulfone (PSF) and polyacrylonitrile (PAN) supports were obtained from a commercial supplier (Origin Water, Megavision Membrane). PVDF and PI supports were made in laboratory. Coverslips with a thickness of 0.1−0.13 mm were purchased from Sail Brano Corp. and used as a support for scanning electron microscopy (SEM).

### 1.2. Characterization methods

#### 1.2.1. Nuclear magnetic resonance (NMR) spectroscopy

Nuclear magnetic resonance NMR (BRUKER, Germany) was conducted to verify the purity of the prepared dendrimers. Approximately 5−15 mg of the prepared product was loaded into the NMR tube and dissolved with 2−3 mL $CDCl_3$ or $(CD_3)_2SO$ for $H^1$ NMR characterization. Specifically, 3,5-bis (trifluoro acetamido) benzoyl chloride was dissolved in $CDCl_3$, while 3,5-bis (N-trifluoro acetamido) benzoic acid, G1 dendrimer (G1D), G2 dendrimer (G2D), G3 dendrimer (G3D) and G4 dendrimer (G4D) were dissolved in $(CD_3)_2SO$. The data were analyzed with MestReNova software.

### 1.2.2. Brunauer Emmett Teller (BET)

The mesoporosity and specific surface area of the G4D dendrimer and PSF support were identified by the pore volume as determined by the Brunauer Emmett Teller (BET) method (ASAP2020 specific surface area and pore analyzer), and the pore size distribution plot of the dendrimer powder was calculated by the quenched solid DFT using the adsorption branch.

### 1.2.3. Scanning electron microscopy observation (SEM)

Thin nanofilm composite membranes are consisted of three layers, an ultrathin polyamide top layer, a PSF support, and a nonwoven fabric. For accurately observing the cross-sectional morphology of the polyamide nanofilm, the nonwoven fabric was first peeled off by using adhesive tape. Then, the remaining PSF support with polyamide layer was soaked in DMF until the polyamide became fully transparent, indicating that the PSF support material was no longer present, and then washed with methanol. For the cross-sectional morphology, the polyamide layers without PSF support were deposited onto the coverslips by a floating method and fractured in liquid nitrogen for scanning electron microscopy observation (SEM, Hitachi SU8010). The samples were coated with gold before SEM analysis.

### 1.2.4. Transmission electron microscope (TEM)

The G4D dendrimer and G4D dendrimer nanoparticles formed by diazotization coupling reaction were deposited onto the copper meshes and investigated by transmission electron microscope (TEM, JEM-1200EX, JEOL). For the internal morphology of the traditional polyamide nanofilm and asymmetric polyamide nanofilm, the polyamide layers without PSF support were deposited onto the copper meshes and investigated by transmission electron microscope (TEM, JEM-1200EX, JEOL).

### 1.2.5. Atomic force microscope (AFM)

A scanning probe microscope (SPM-9700, SHIMADZU) was used to measure the surface morphology and the roughness of the polyamide membranes.

### 1.2.6. Attenuated total reflectance fourier transform infrared spectroscopy (ATR-FTIR) and X-ray photoelectron spectroscopy (XPS)

The chemical composition and structure of the PSF, PSF-G4D-1, PSF-G4D-2, PSF-G4D-3, and the fabricated polyamide membranes (including the traditional polyamide membrane and asymmetric polyamide membrane) were characterized by attenuated total reflectance fourier transform infrared spectroscopy (ATR-FTIR) and X-ray photoelectron spectroscopy (XPS), respectively. The chemical composition and elemental data obtained from XPS were analyzed and fitted using CasaXPS software.

### 1.2.7. UV-visible spectroscopy

UV-visible diffuse reflectance spectroscopy (UV-vis DRS) was conducted to character the chemical composition variation of the PSF, PSF-G4D-1, PSF-G4D-2 and PSF-G4D-3 support membranes. UV-vis spectra were conducted to monitor the diazotization coupling reaction in the solution.

### 1.2.8. Zeta potential

Surface charges of the traditional and asymmetric polyamide membrane were determined with an Anton Paar SurPass solid surface analyzer.

### 1.2.9. Contact angle

Contact angle was measured under room temperature (25°C) using a drop shape Analyzer-DSA30 (KRÜSS, Germany) in the sessile drop mode for characterizing the surface hydrophilicity and surface energy of the membrane surface.

## 2. Supplementary Experimental

### 2.1. Synthesis steps of G4 dendrimer (G4D)

#### 2.1.1. Synthesis steps of 3,5-bis (N-trifluoro acetamido) benzoic acid

Trifluoroacetic anhydride (78.6 mmol) was added to a 30 mL THF solution containing 3,5-diaminobenzoic acid (24 mmol) at 0°C under nitrogen, and stirred at that temperature for 15 min. Subsequently, the system was stirred for 3 h at 25°C oil bath. Then, water (30 mL) was added and continued to be stirred for 6h at that temperature. The resulted mixture was extracted with the ethyl acetate to obtain the organic layer and aqueous layer. The organic layer was washed with water 3–4 times, and dried with anhydrous magnesium sulfate overnight. Afterwards, the resulted filtrate was evaporated to give a purple powdery solid, and recrystallized from acetonitrile, filtrated and gave purple solid particles. The product was dried at 120°C for 12 h to give pale purple solid particles (6.1 g, a yield of 79%).

#### 2.1.2. Synthesis steps of 3,5-bis (trifluoro acetamido) benzoyl chloride

A 150 mL of thionyl chloride solution containing 3,5-bis (N-trifluoro acetamido) benzoic acid (46.6 mmol) was refluxed for 6 h at 120°C. The thionyl chloride is distilled off and the residue was dissolved in tetrachloroethane at 100°C, Subsequently, cooled to room temperature to precipitate a purple solid powder. The solid was washed with n-hexane three times to give a brown powder. Afterwards, the power was recrystallized with dichloromethane, dried at 60°C to give slightly yellow power (12.1 g, a yield of 72%).

#### 2.1.3. Synthesis steps of G1 dendrimer (G1D)

3,5-bis (N-trifluoro acetamido) benzoic chloride (22 mmol) was added to a 10 mL of NMP solution containing P-phenylenediamine (10 mmol), stirred for 15 min at 0°C, and subsequently stirred for 1 h at 25°C oil bath. The water (50 μL) was added to the system and reacted for 1.5 h at 50°C. Then, hydrazine hydrate (120 mmol) was added dropwise and continued to be stirred for 1.5 h at that temperature. The reaction solution was poured into a 100 mL of 2 wt% $NaHCO_3$ solution, stirred for 30 min, filtered under suction, and dried to give light gray solid of G1D (7.4 g, yield 98%).

### 2.1.4. Synthesis steps of G2 dendrimer (G2D)

3,5-bis (N-trifluoro acetamido) benzoic chloride (22 mmol) was added to a 20 mL of NMP solution containing G1 (5.0 mmol), stirred for 15 min at 0°C, and subsequently stirred for 1 h at 25°C oil bath. The water (50 μL) was added to the system and reacted for 1.5 h at 50°C. Then, hydrazine hydrate (120 mmol) was added dropwise and continued to be stirred for 1.5 h at that temperature. The reaction solution was poured into a 100 mL of 2 wt% $NaHCO_3$ solution, stirred for 30 min, filtered under suction, and dried to give light gray solid of G2D (4.4 g, yield 97%).

### 2.1.5. Synthesis steps of G3 dendrimer (G3D)

3,5-bis (N-trifluoro acetamido) benzoic chloride (22 mmol) was added to a 27 mL of NMP solution containing G2 (2.5 mmol), stirred for 15 min at 0°C, and subsequently stirred for 1 h at 25°C oil bath. The water (50 μL) was added to the system and reacted for 1.5 h at 50°C. Then, hydrazine hydrate (120 mmol) was added dropwise and continued to be stirred for 3.5 h at that temperature. The reaction solution was poured into a 133 mL of 2 wt% $NaHCO_3$ solution, stirred for 30 min, filtered and dried to give light gray solid of G3D (4.7 g, yield 94%).

### 2.1.6. Synthesis steps of G4 dendrimer (G4D)

3,5-bis (N-trifluoro acetamido) benzoic chloride (22 mmol) was added to a 27 mL of NMP solution containing G3 (1.3 mmol), stirred for 15 min at 0°C, and subsequently stirred for 1 h at 25°C oil bath. The water (50 μL) was added to the system and reacted for 1.5 h at 50°C. Then, hydrazine hydrate (120 mmol) was added dropwise and continued to be stirred for 3.5 h at that temperature. The reaction solution was poured into a 133 mL of 2 wt% $NaHCO_3$ solution, stirred for 30 min, filtered and dried to give light gray solid of G4D (4.8 g, yield 92%).

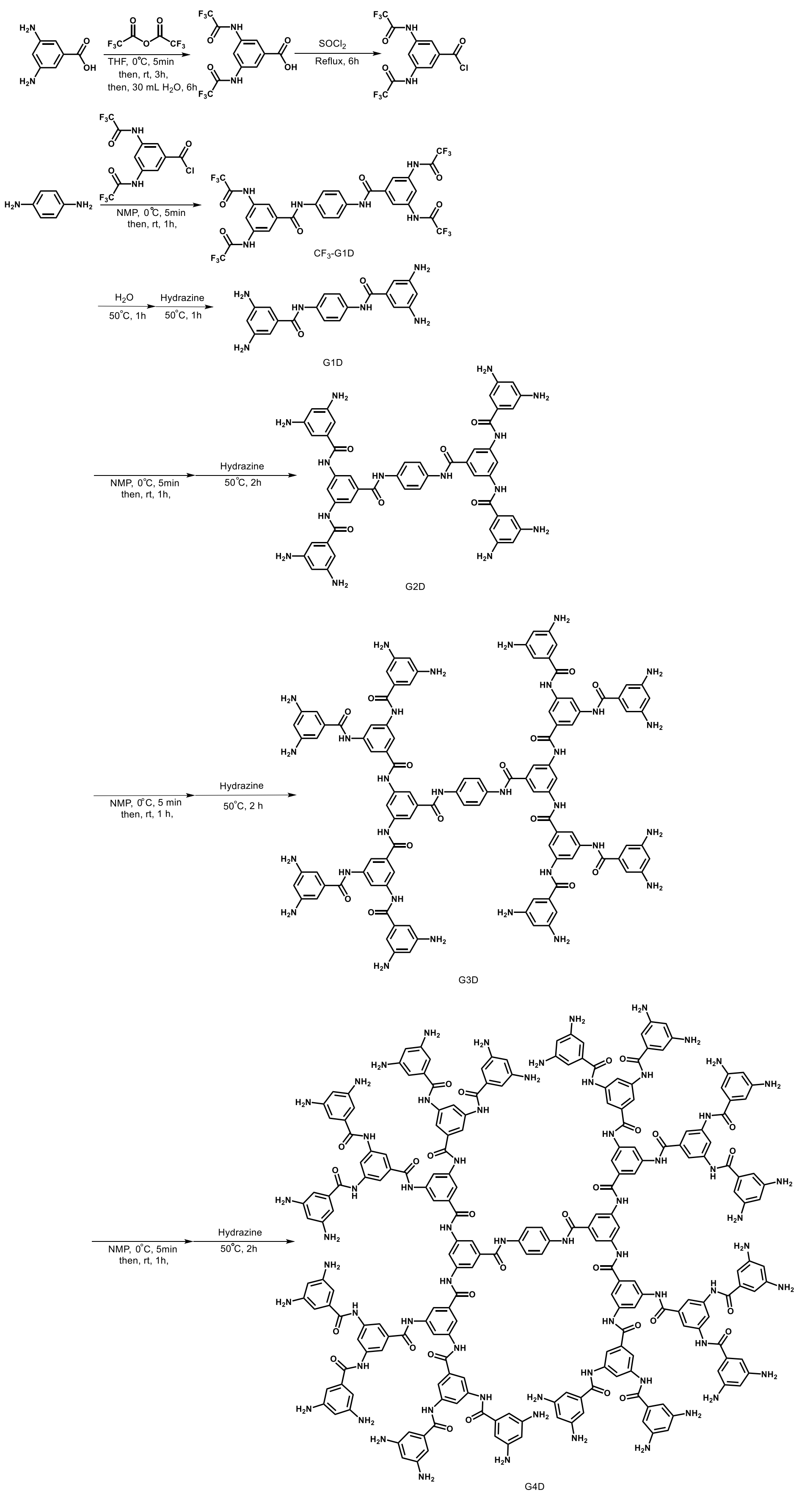


**Supplementary Figure 1.** Synthesis steps of dendrimer G4D.

## 2.2. Fabrication of PVDF and PI supports.

P84 casting solution was prepared by dissolving 18 wt% of P84, 4 wt% of PVP K60 and 6 wt% of PEG 600 in DMF under stirring at 70°C for 12 h, and then degassed for a further 12 h at 45°C to eliminate any air bubble trapped in the solutions. After cooled to room temperature, the dope solutions were cast on non-woven by using a casting knife with a fixed thickness of 110 μm. Then the fresh scraped film solution was allowed to parallel immersion into a precipitation water bath at room temperature. The PVDF casting solution with 20 wt% of PVDF, 4 wt% of PVP K60 and 6 wt% of PEG 600 was dissolved in DMF under stirring at 70°C for 12 h, and then conducted the similar process to fabricate the PVDF support. All fabricated supports were washed with distilled water for 3 h to remove any residual solvent for further use.

## 2.3. Preparation of asymmetric polyamide RO membrane.

We used m-Phenylenediamine (MPD) to synthesize the asymmetric polyamide RO membrane. Specifically, the PSF and PSF-G4D-1 substrates were firstly immersed into the amine solution (2 w/v% MPD, 1.1 w/v% TEA and 2.3 w/v% CSA) for 4 min, and subsequently the extra amine solution on the support surface was blown off with an air knife. Then, the above support substrate was contacted with the TMC/n-octane solution (0.12 w/v%) for 40s to form the traditional or asymmetric polyamide nanofilm, and the organic phase solution on the surface was instantly blown off with an air knife. The resulted polyamide membranes were finally dried at 60°C for 2–5 min and stored in DI water until use.

## 2.4. Membrane performance test

For the polyamide RO membranes, desalination performance of the prepared polyamide membrane was determined with different salt solutions in a cross-flow system with an effective test area (A) of 19.3 $cm^2$. The NaCl concentration in the feed solution was 2 g $L^{-1}$. The desalination performance tests were conducted at an osmotic pressure of 1.55 MPa and a temperature of 25°C. The performance data were determined after the water flux and the conductivity reached a steady state.

The water flux (kg $m^{-2}$ $h^{-1}$) was calculated from the weight of the permeate (M) for a

specified time, as given by the following equation:

Water Flux (kg $m^{-2}$ $h^{-1}$) = $M/At$.                (1)

The salt rejection was determined from the conductivity of the feed solution ($C_f$) and the permeate ($C_p$). Hence, the salt/ion rejection can be calculated from the following equation:

Rejection (%) = $\left(1\text{-}C_p/C_f\right)\times 100\%$.    (2)

## 2.5. Density measurement

Densities of polyamide dendrimer porous layer, traditional polyamide nanofilm and asymmetric polyamide nanofilm were measured and calculated by the ellipsometry (J. A. Woollam Co., Lincoln, NE) and QCM (Q-Sense, Explorer, Biolin Scientific). We first used the floating method to isolate and deposit the polyamide dendrimer porous layer, traditional polyamide nanofilm and asymmetric polyamide nanofilm onto the QCM sensors, and then analyzed the change in the frequency of vibration of QCM sensors to obtain the surface density of the samples. After that, we transferred the QCM sensors having deposited polyamide layer onto the ellipsometry platform to conduct the thickness measurement. Finally, by dividing the areal density by the thickness of the polyamide layer, we can calculate the layer density. In an effort to accurate, five locations on each sample were analyzed.

## 3. Supplementary figures and data

### 3.1. Characterization of G4 dendrimer (G4D)

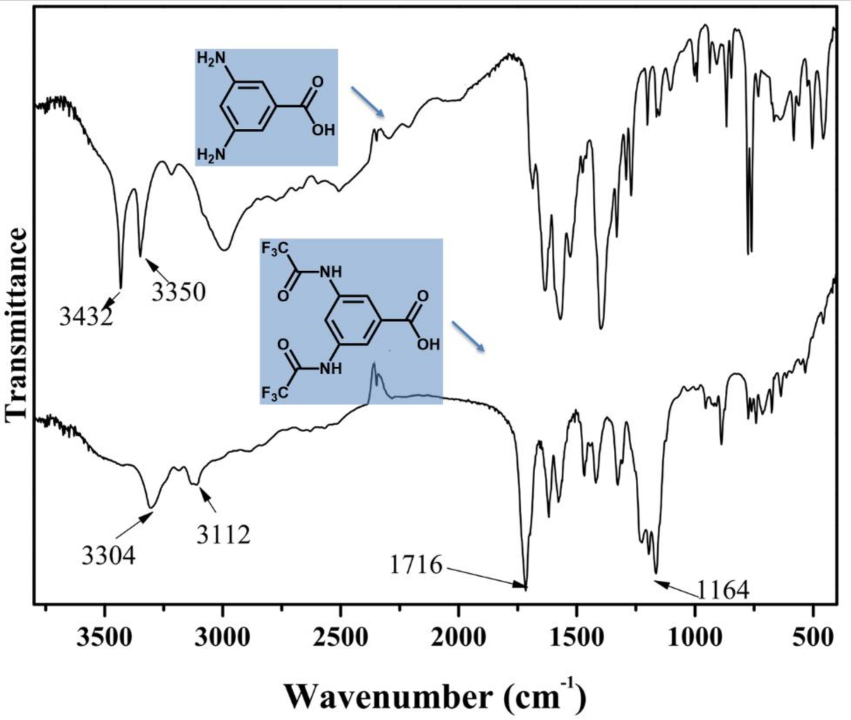


**Supplementary Figure 2.** FT-IR spectrum of 3,5-diaminobenzoic acid and 3,5-bis (trifluoro acetamido) benzoic acid.

It can be seen from Supplementary Figure 2 that the 3,5-bis (trifluoro acetamido) benzoic acid are obtained by modifying the amine group with trifluoroacetic anhydride. For 3,5-bis (trifluoro acetamido) benzoic acid, the C-F absorption peak appeared at 1164 $cm^{-1}$, the N-H absorption peak is at 3304 $cm^{-1}$. For 3,5-diaminobenzoic acid, the N-H absorption peaks are located at 3350 $cm^{-1}$ and 3432 $cm^{-1}$. Changes in these characteristic peaks indicate that the amino protection reaction has been completed, that is, 3,5-bis (trifluoro acetamido) benzoic acid with a protecting group $CF_3$ was formed.

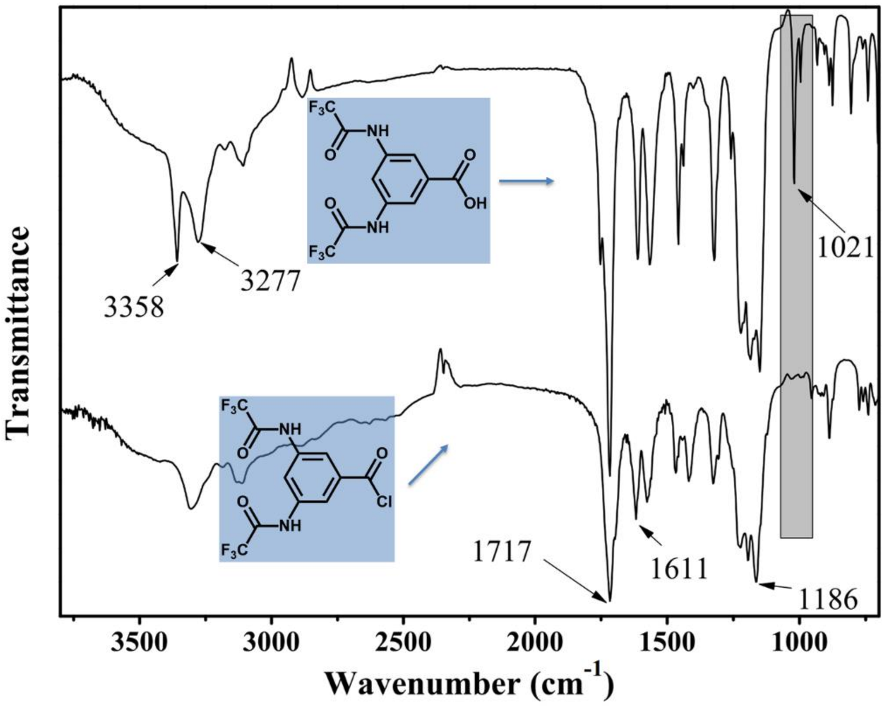


**Supplementary Figure 3.** FT-IR spectrum of 3,5-bis (trifluoro acetamido) benzoic acid and 3,5-bis (trifluoro acetamido) benzoic acid chloride.

The FT-IR spectrum of 3,5-bis (trifluoro acetamido) benzoic acid chloride is shown in Supplementary Figure 3. Compared to 3,5-bis (trifluoro acetamido) benzoic acid, the 3,5-bis (trifluoro acetamido) benzoic acid chloride has a characteristic absorption peak of the acid chloride group at 1021 $cm^{-1}$, and another characteristic peaks at 1186 $cm^{-1}$ (CF), 1611 $cm^{-1}$ (Ar-H), 1717 $cm^{-1}$ (C = O), and 3277 $cm^{-1}$ (-NH-) , further verifies that the acyl chloride reaction has been completed.

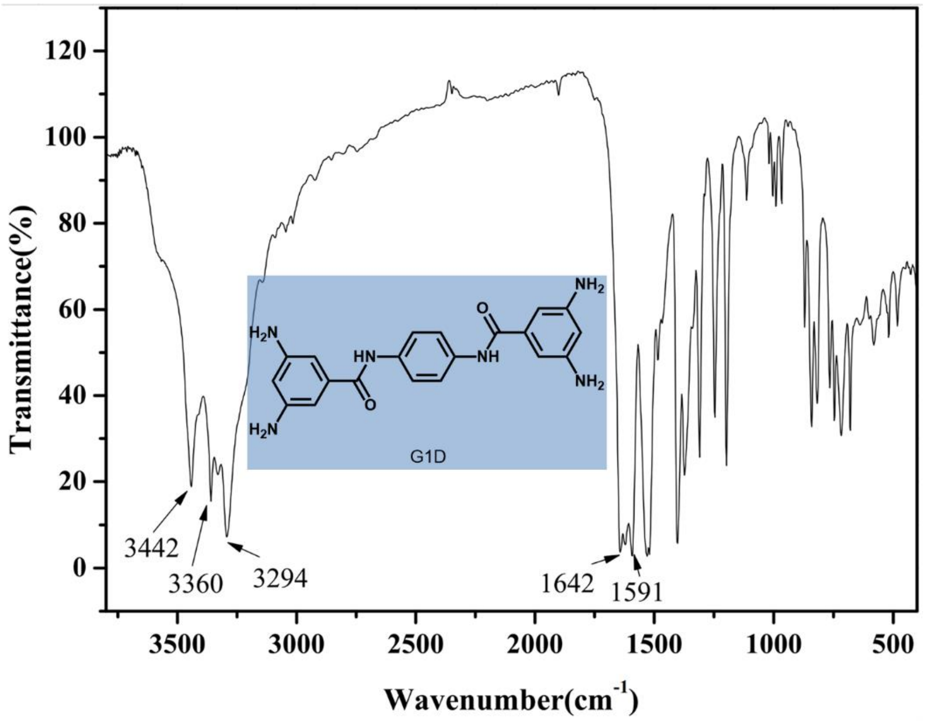


**Supplementary Figure 4.** FT-IR spectrum of G1D.

The FT-IR spectrum of G1D is shown in Supplementary Figure 4. Among them, the characteristic peaks of G1D products are respectively located at 1591 $cm^{-1}$ (Ar-H), 1642 $cm^{-1}$ (C = O), and 3442 $cm^{-1}$ (NH). The presence of these characteristic peaks indicates that after the reaction with the acid chloride monomer, the terminal amine group of p-phenylenediamine is indeed connected to the 3,5-bis (trifluoro acetamido) benzoic acid chloride. And after deprotection reaction, the number of terminal amine groups of the first generation has been doubled. It is also worth noting that there is no characteristic absorption peak of the C-F group in the infrared spectrum of the G1D products, which also indicates that the deprotection reaction is carried out thoroughly.

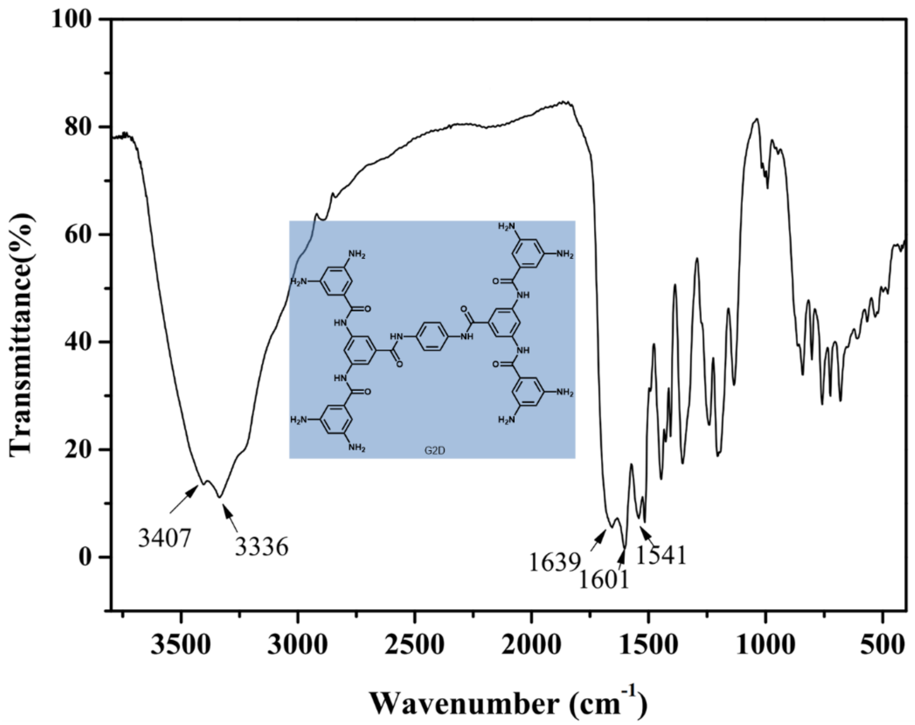


**Supplementary Figure 5.** FT-IR spectrum of G2D.

The FT-IR spectrum of G2D is shown in Supplementary Figure 5. Among them, the characteristic peaks of G2D products are respectively located at 1541 $cm^{-1}$ (Ar-H), 1639 $cm^{-1}$ (C = O), and 3336 $cm^{-1}$ (NH). The presence of these characteristic peaks indicates that after the reaction with the acid chloride monomer, the terminal amine group of G1D is indeed connected to the 3,5-bis (trifluoro acetamido) benzoic acid chloride. And after deprotection reaction, the number of terminal amine groups of the first generation has been doubled. It is also worth noting that there is no characteristic absorption peak of the C-F group in the infrared spectrum of the G2D products, which also indicates that the deprotection reaction is carried out thoroughly.

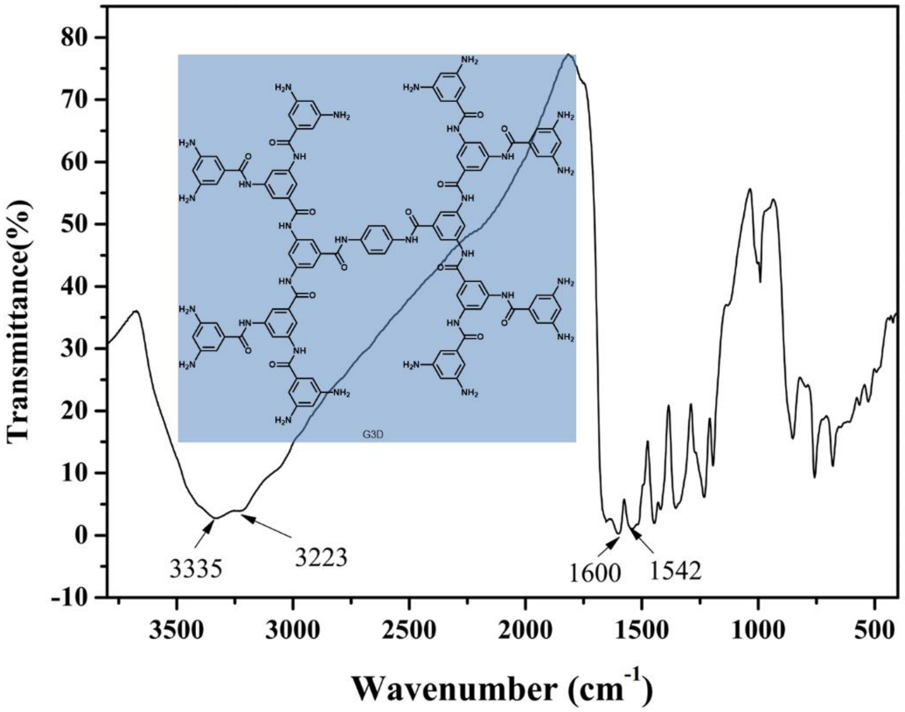


**Supplementary Figure 6.** FT-IR spectrum of G3D.

The FT-IR spectrum of G3D is shown in Supplementary Figure 6. Among them, the characteristic peaks of G3D products are respectively located at 1542 $cm^{-1}$ (Ar-H), 1600 $cm^{-1}$ (C = O), and 3335 $cm^{-1}$ (NH). The presence of these characteristic peaks indicates that after the reaction with the acid chloride monomer, the terminal amine group of G2D is indeed connected to the 3,5-bis (trifluoro acetamido) benzoic acid chloride. And after deprotection reaction, the number of terminal amine groups of the G2D has been doubled. It is also worth noting that there is no characteristic absorption peak of the C-F group in the infrared spectrum of the G3D products, which also indicates that the deprotection reaction is carried out thoroughly.

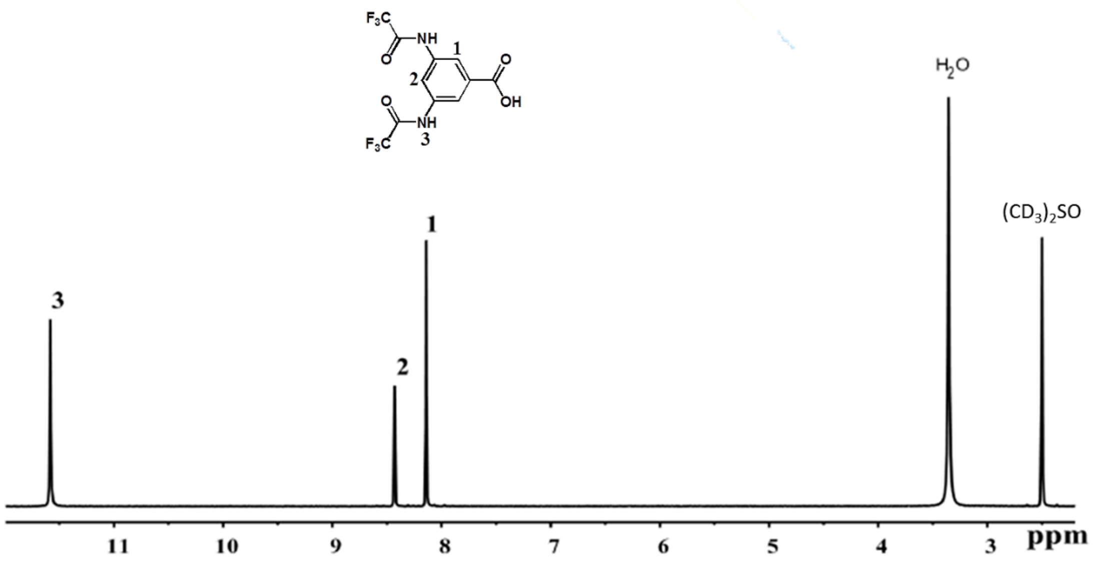


**Supplementary Figure 7.** $^{1}H$ NMR spectrum of 3,5-bis (trifluoro acetamido) benzoic acid.

Supplementary Figure 7 shows the $^{1}H$ NMR chart of 3,5-bis (N-trifluoro acetamido) benzoic acid. Among them, the peak at 8.14 ppm is the proton peak adjacent to the carboxyl group on the benzene ring, the peak at 8.42 ppm is the proton peak at the para position of the carboxyl group on the benzene ring, and the peak at 11.58 ppm is the proton peak of trifluoro acetamido.

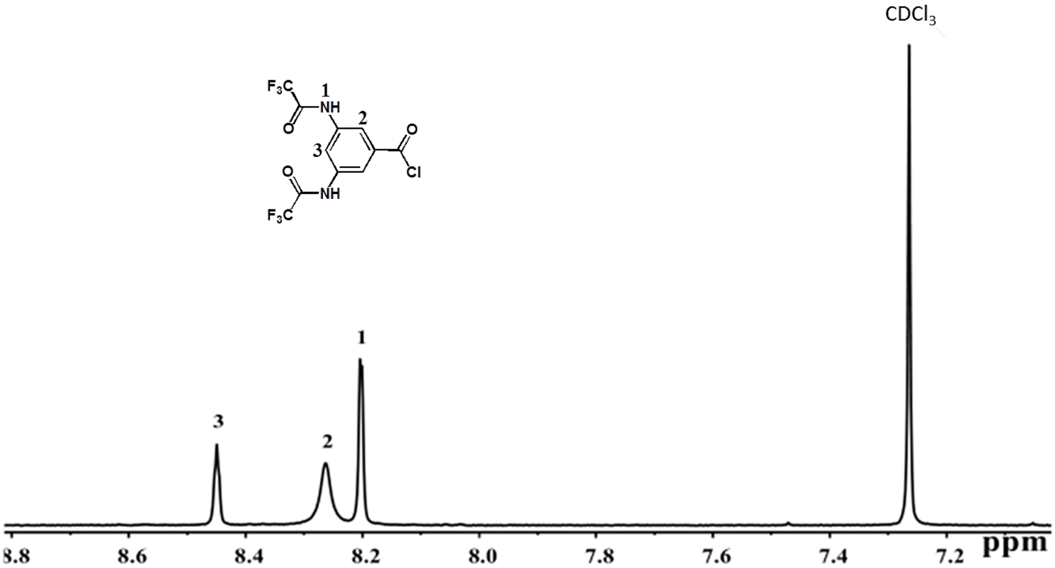


**Supplementary Figure 8.** $^1$H NMR spectrum of 3,5-bis (trifluoro acetamido) benzoyl chloride.

The $^1$H NMR spectrum of the 3,5-bis (trifluoro acetamido) benzoyl chloride is shown in Supplementary Figure 8. The peak at 8.20 ppm is the proton peak adjacent to the acid chloride on the benzene ring, the peak at 8.27 ppm is the proton peak for trifluoro acetamido, and the peak at 8.45 ppm is the proton at the para position on the benzene ring peak.

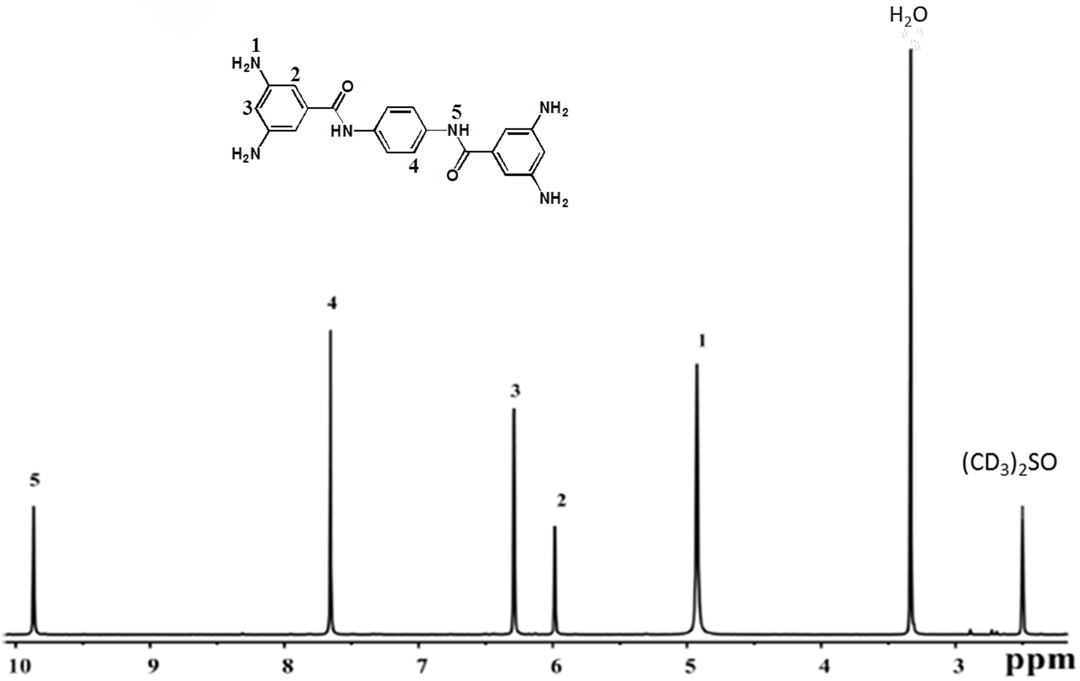


**Supplementary Figure 9.** $^{1}H$ NMR spectrum of G1 dendrimer (G1D).

The $^{1}H$ NMR spectrum of the G1D product is shown in Supplementary Figure 9. It is found that the characteristic absorption peak of the terminal amine group is at 4.93 ppm, 6.0 ppm and 6.3 ppm are characteristic absorption peaks of hydrogen on the outer benzene ring. In addition, 7.7 ppm is the characteristic absorption peak of hydrogen on p-phenylenediamine benzene ring, and the absorption peak of amide group on p-phenylenediamine is at 9.9 ppm.

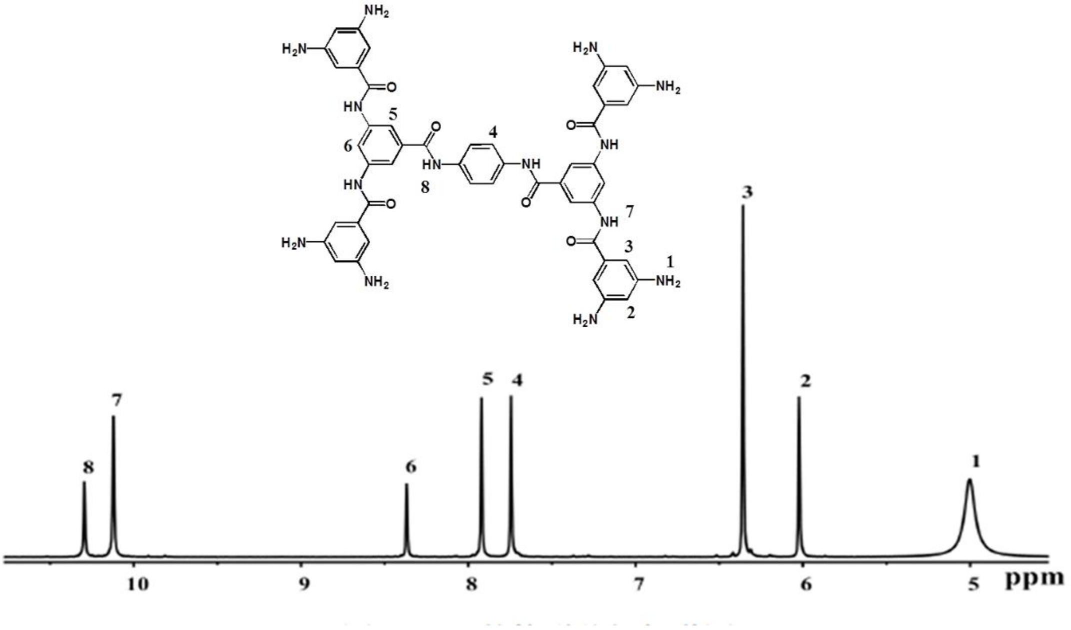


**Supplementary Figure 10.** $^1$H NMR spectrum of G2 dendrimer (G2D).

The $^1$H NMR spectrum of G2D is shown in Supplementary Figure 10, where 5.0 ppm is the proton peak of the terminal amine group, 6.0 ppm and 6.4 ppm are the proton peaks on the outermost benzene ring, and 7.7 ppm is the peak of hydrogen on p-phenylenediamine benzene ring, at 7.9 ppm and 8.4 ppm are the proton peaks on the middle layer benzene ring, at 10.1 ppm are the proton peaks of the middle layer amide group, and at 10.3 ppm are the proton peaks of the inner layer amide group.

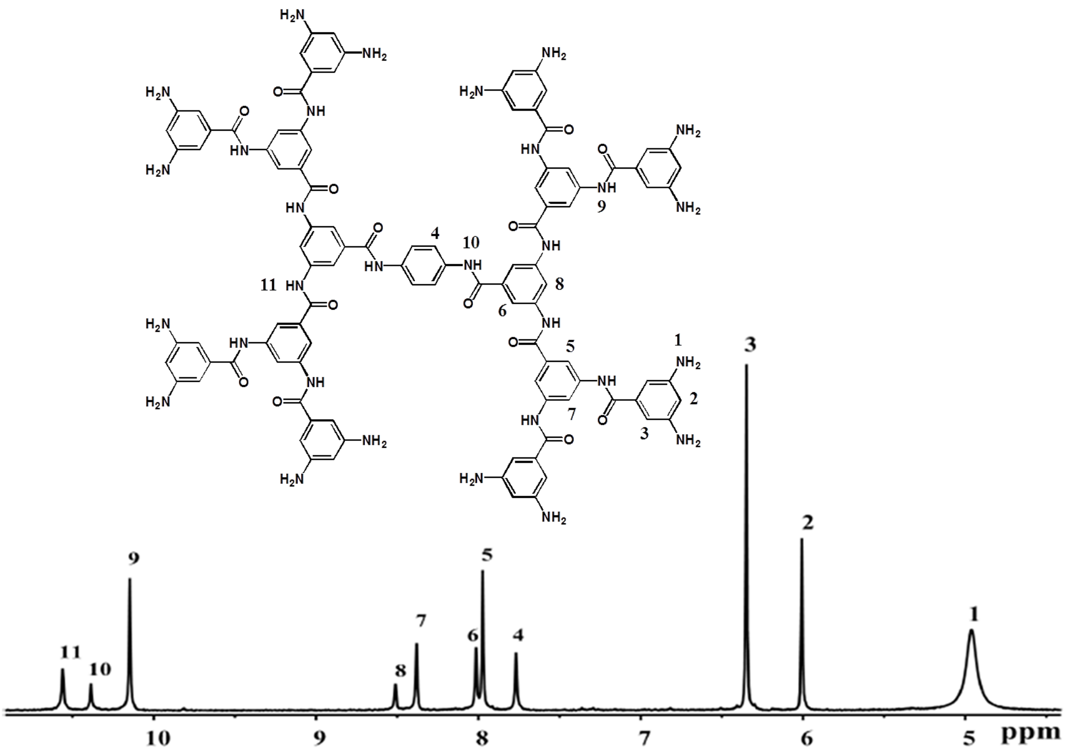

**Supplementary Figure 11.** $^1$H NMR spectrum of G3 dendrimer (G3D).

The $^1$H NMR spectrum of $NH_2$-G3D is shown in Supplementary Figure 11. Among them, 5.0 ppm is the proton peak of the terminal amine group, 6.0 ppm and 6.3 pm are the absorption peaks of hydrogen on the outermost benzene ring, 7.8 ppm is the proton peak on the phenylenediamine benzene ring, 7.9 ppm and 8.4 ppm is the proton peak on the second generation benzene ring, 8.0 ppm is 8.5 ppm is the proton peak on the first generation benzene ring, 10.1 ppm is the proton peak of the outer amide group, and 10.4 ppm is the proton peak of the inner amide group, 10.6 ppm is the proton peak of the amide group in the middle layer.

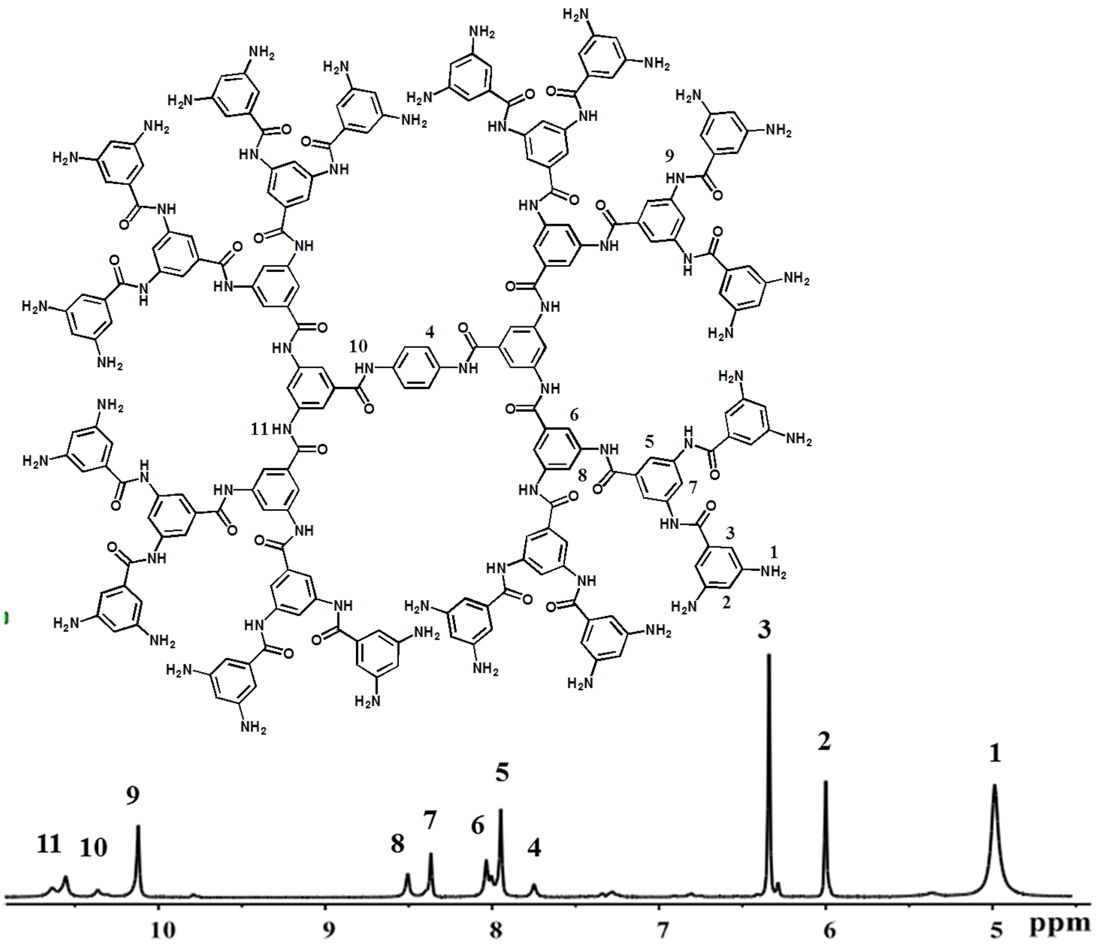

**Supplementary Figure 12.** $^1$H NMR spectrum of G4 dendrimer (G4D).

The $^1$H NMR spectrum of G4D is shown in Supplementary Figure 12. Among them, 5.0 ppm is the proton peak of the terminal amine group, 6.0 ppm and 6.3 pm are the absorption peaks of hydrogen on the outermost benzene ring, 7.8 ppm is the proton peak on the phenylenediamine benzene ring, 7.9 ppm and 8.4 ppm is the proton peak on the second generation benzene ring, 8.0 ppm is 8.5 ppm is the proton peak on the first generation benzene ring, 10.2 ppm is the proton peak of the outer amide group, and 10.4 ppm is the proton peak of the inner amide group, 10.6 ppm is the proton peak of the amide group in the middle layer.

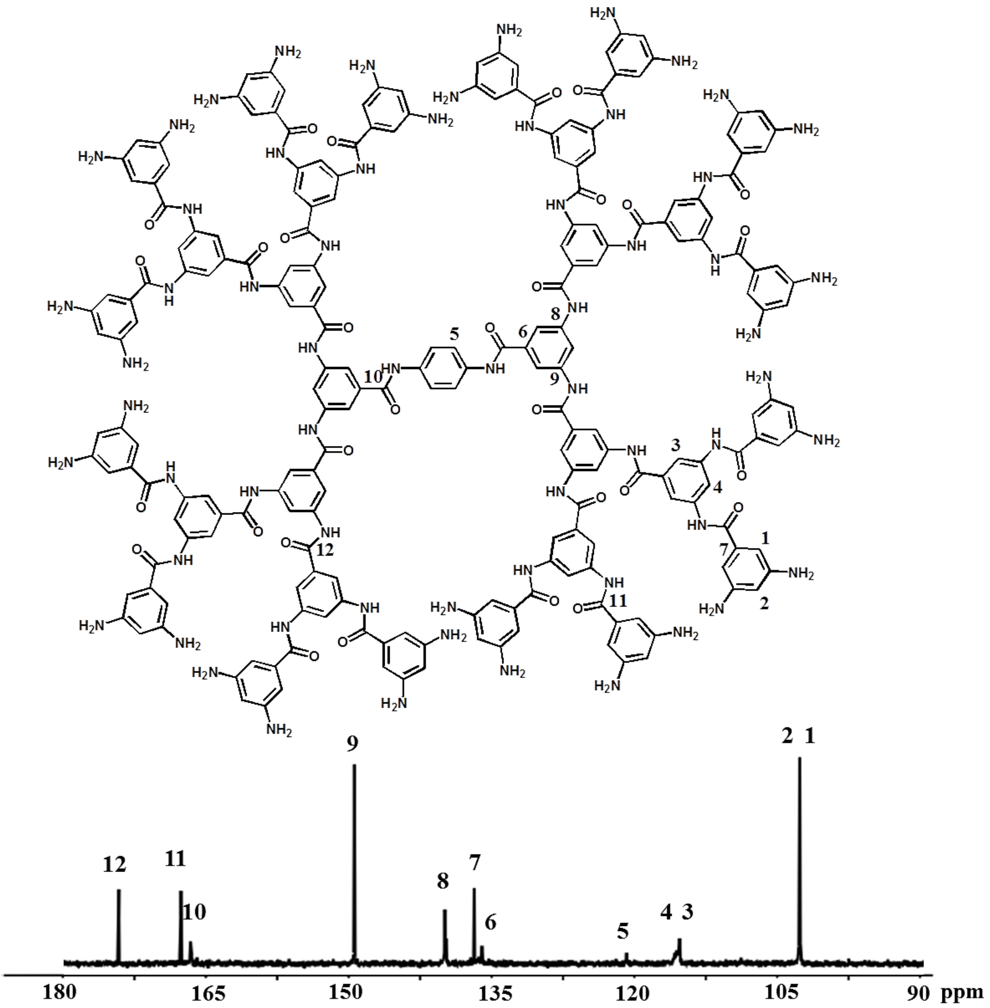


**Supplementary Figure 13.** $^{13}C$ NMR spectrum of G4 dendrimer (G4D).

The $^{13}C$ NMR spectrum of G4D is shown in Supplementary Figure 13. Among them, 101.1 ppm is the carbon absorption peak of the terminal benzene ring, 115.8 ppm is the absorption peaks of carbon on the benzene ring of G3D, 121.1 ppm is the carbon absorption peak on the phenylenediamine benzene ring, 136.1 ppm and is the carbon absorption peak on the first generation benzene ring, 137.7 ppm is the carbon absorption peak on the terminal benzene ring, 139.7 ppm and 149.0 ppm are the carbon absorption peak of the first generation benzene ring, and 166.5 ppm, 167.7 ppm and 174.2 ppm are the carbon absorption peak of the amide group of the G1D, G2D/G3D and G4D, respectively.

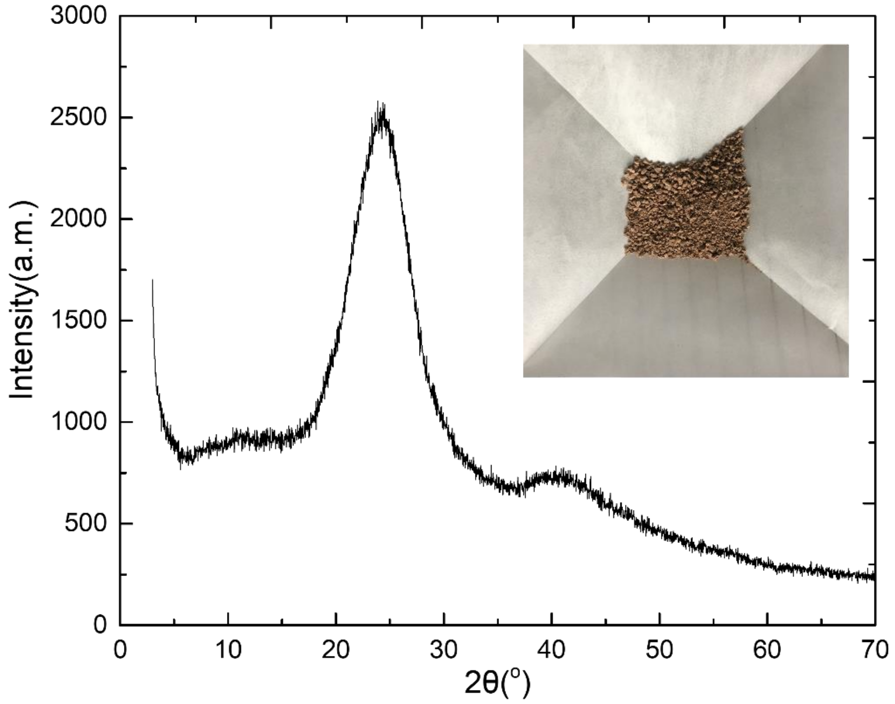


**Supplementary Figure 14.** XRD spectra of G4 dendrimer (G4D).

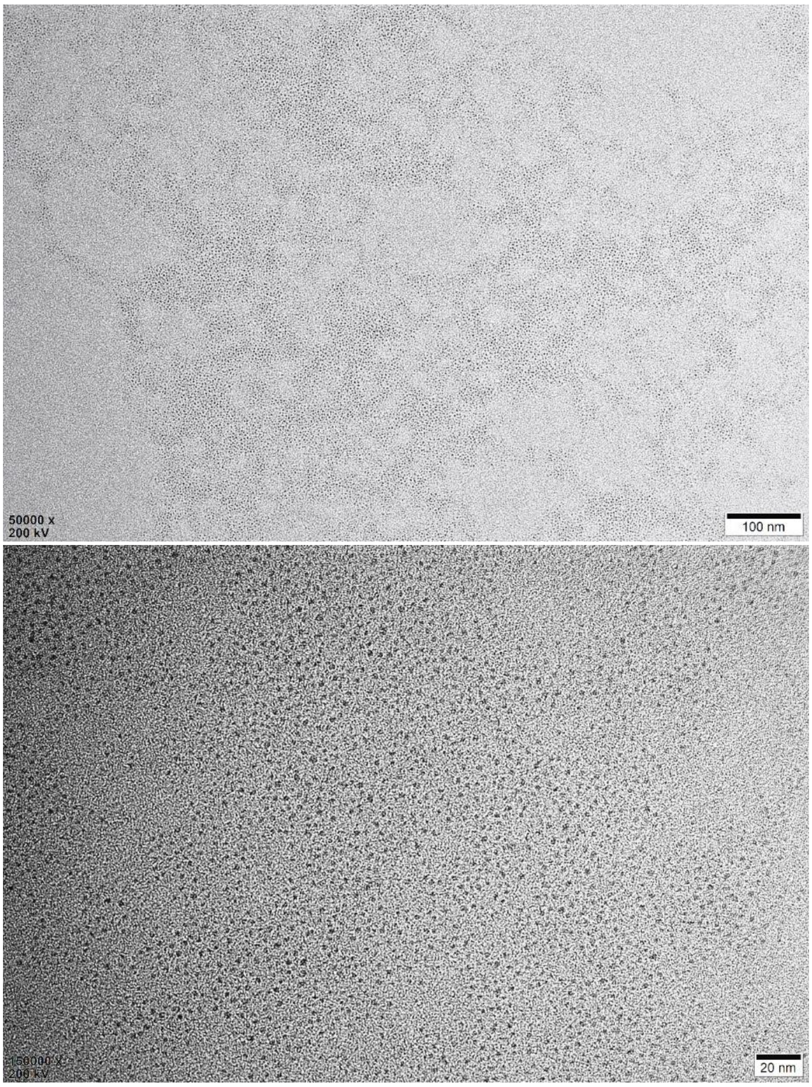


**Supplementary Figure 15.** TEM images of dendrimer G4D.

As shown in Supplementary Figure 15, the single G4D dendrimer nanoparticles are evenly dispersed on the copper mesh.

### 3.2. Diazotization coupling reaction of solution

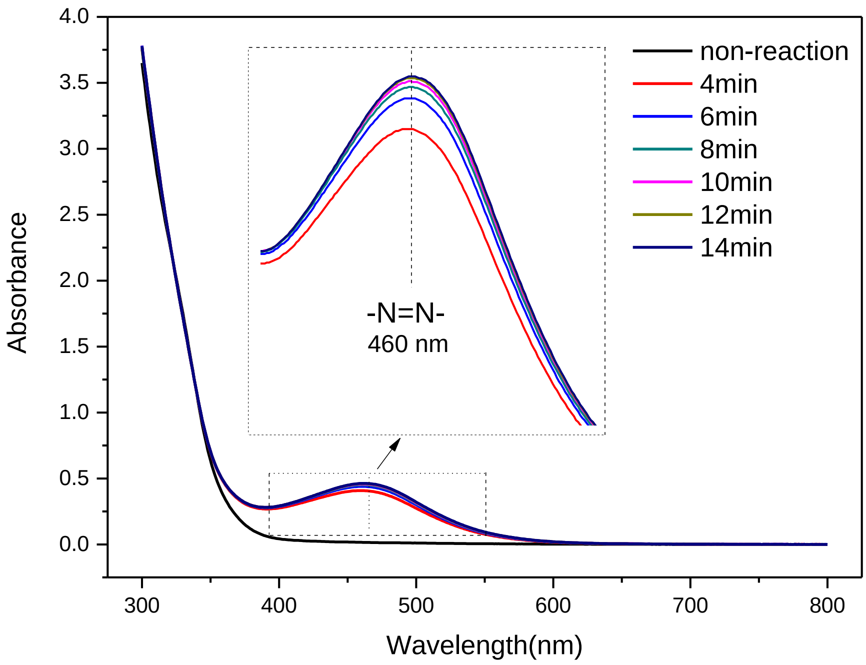


**Supplementary Figure 16.** UV visible spectra of the dendrimer salt solution before and after the addition of the sodium nitrite solution with pH=1, 0°C.

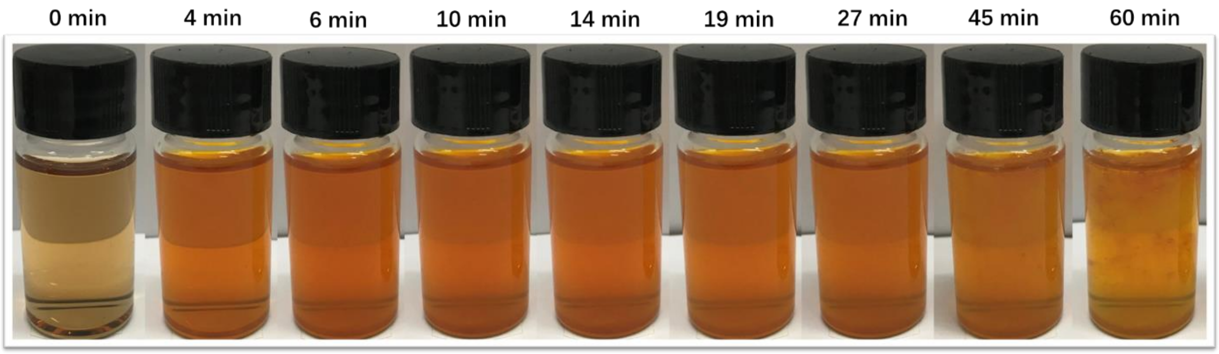


**Supplementary Figure 17.** Digital pictures of the dendrimer salt solution before and after the addition of the sodium nitrite solution with pH=1, 0°C.

As shown in Supplementary Figure 16, we conducted the diazotization coupling reaction in the solution, and monitored the change of diazo bond strength with time using UV visible spectrophotometer. From the diazo bond strength variation, we can indirectly infer the reactivity of dendrimers during diazotization-coupling reaction. In the initial stage of the diazotization reaction (4 min), the peak intensity of the diazonium bond reached a larger absorption value at the wavelength of 460 nm[1]. After 4 min, the

peak intensity of the diazonium bond increased slightly, 10 min after that, the intensity of the diazo bond peak no longer changes. Digital pictures in Supplementary Figure 17 clearly illustrates this change process. These results indicate that the dendrimer has high reactivity and reaction rate in the initial stage of the diazotization coupling reaction, and the activity gradually decreases as the reaction progresses. This is because dendrimers are macromolecules, and there is a large steric hindrance during the reaction process, which inhibits the progress of the reaction.

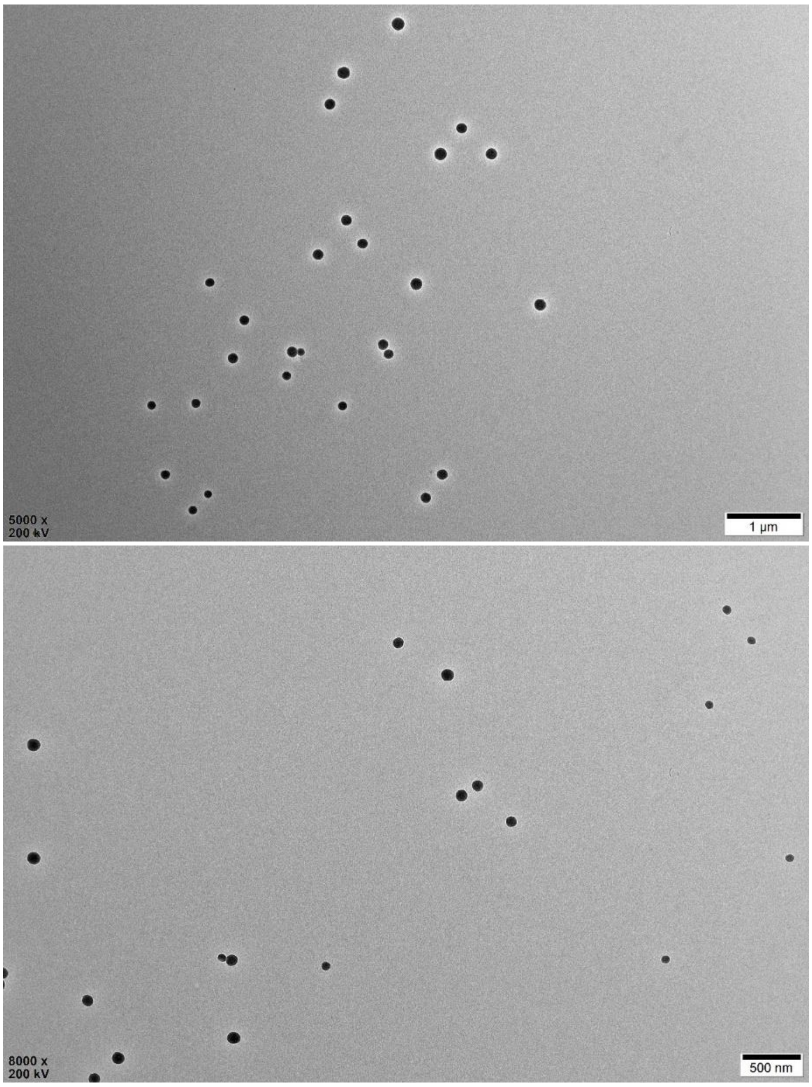


**Supplementary Figure 18.** TEM images of dendrimer G4D nanoparticles formed by diazotization coupling reaction.

As shown in Supplementary Figure 18, the morphology of the dendrimer G4D nanoparticles formed by diazotization coupling reaction was investigated. The sodium nitrite solution (72.5 mM, pH=1) at 0°C was added in the G4D solution (0.242 mM, pH=1) under the reaction time of 5 min to form the polyamide dendrimer nanoparticles. The size of the polyamide dendrimer nanoparticles was in the range of 98 to 125 nm. This result indicates that dendrimer G4D can form the polyamide dendrimer nanoparticles through the diazotization coupling reaction.

### 3.3. Mechanism of dendrimers anchored covalently on PSF support

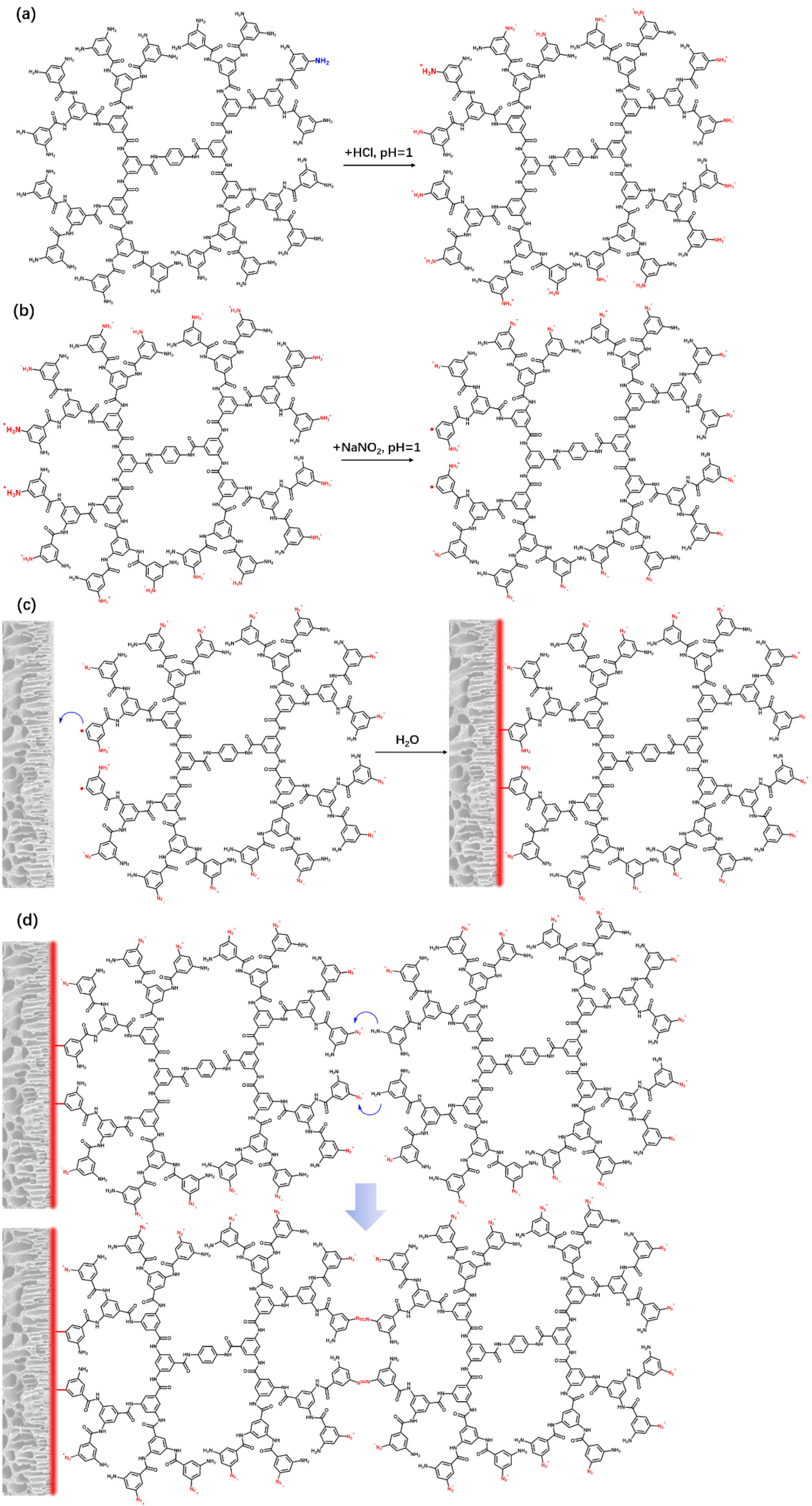
(a)
+HCl, pH=1
(b)
+NaNO2, pH=1
(c)
H2O
(d)

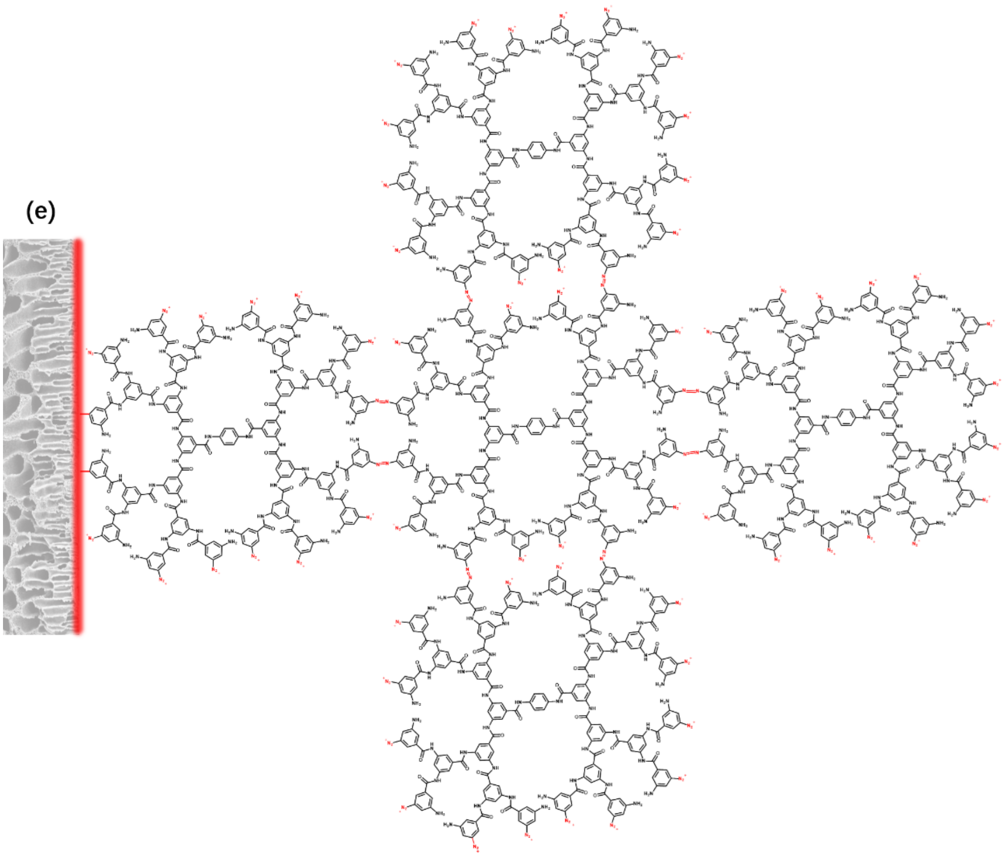

**Supplementary Figure 19.** Chemical route to form anchored covalently dendrimer porous layer on PSF support: (a) formation of soluble dendrimer salt solution at pH=1, (b) formation of aryldiazonium salt, (c) Oxidation of the aryldiazonium group through redox mechanism with excess $NaNO_2$, and grafting of the resulting aryl radicals onto the PSF surface, (d) Coupling diazonium salt with aromatic amine groups to form the diazonium bound, (e) growth of the dendrimer porous layer.

We have drawn the chemical route to present the mechanism that how the dendrimers were anchored covalently on PSF support. The specific mechanism is summarized in Supplementary Figure 19. Generally, the anchoring covalently dendrimer on PSF support process are 5 steps, and the following is the details on mechanism[2]:

(a)The addition of one equivalent of HCl leads to the conversion of one of the two amine groups to the corresponding amine salt, which results in the dissolution of the dendrimer; (b) the addition of $NaNO_2$ at pH=1, 0°C, leads to the conversion of amine salt to the corresponding aryldiazonium salt. It is important to point out that the acidic medium protects the remaining amine group from reacting with newly formed

diazonium salts, which is likely to conduct the self-coupling reaction and form the dendrimer porous layer. Chemical oxidation of aryl diazonium salts and protons at the excess $NaNO_2$ solution leading to the formation of the corresponding radical species; (c) formation of the anchored dendrimer-like layer; (d) the diazonium salt couples with the remaining aromatic amine groups in the dendrimer to form a diazonium bond, and leads to the self-assembly reaction of dendrimers; (e) growth of the anchored dendrimer porous layer. The modified PSF UF membrane with dendrimer porous layer was finally rinsed by deionized water to desorb the excess NaCl.

### 3.4. Structure and morphology of the PSF and modified PSF supports.

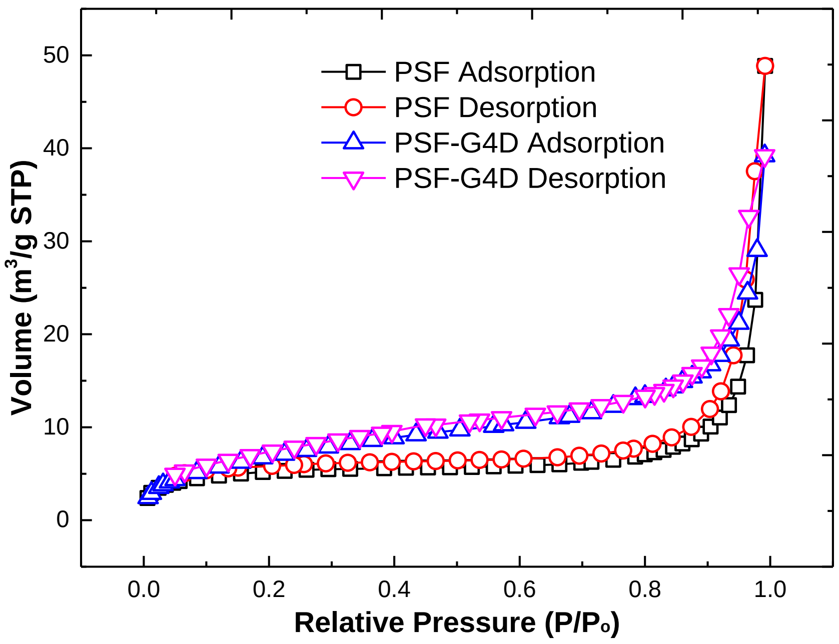


**Supplementary Figure 20.** $N_2$ sorption at 77 K for the pristine PSF support and the PSF-G4D-1 support.

**Supplementary Table 1.** BET Surface Area of the PSF support, PSF-G4D-1 support and G4D dendrimer.

| | PSF support | PSF-G4D-1 support | G4D dendrimer |
|---|---|---|---|
| BET Surface Area ($m^2$ $g^{-1}$) | 17 | 25.7 | 140.9 |

As shown in Supplementary Figure 20 and Table 1, due to the porosity of the polyamide dendrimer G4D, the BET surface area of the PSF-G4D-1 with a dendrimer porous layer is larger than the original PSF support. Specifically, the BET Surface Area of the pristine PSF support is 17 $m^2$ $g^{-1}$, whereas that of the PSF-G4D-1 support is 25.7 $m^2$ $g^{-1}$. This result gives a positive effect on the pure water flux of PSF-G4D-1 support.

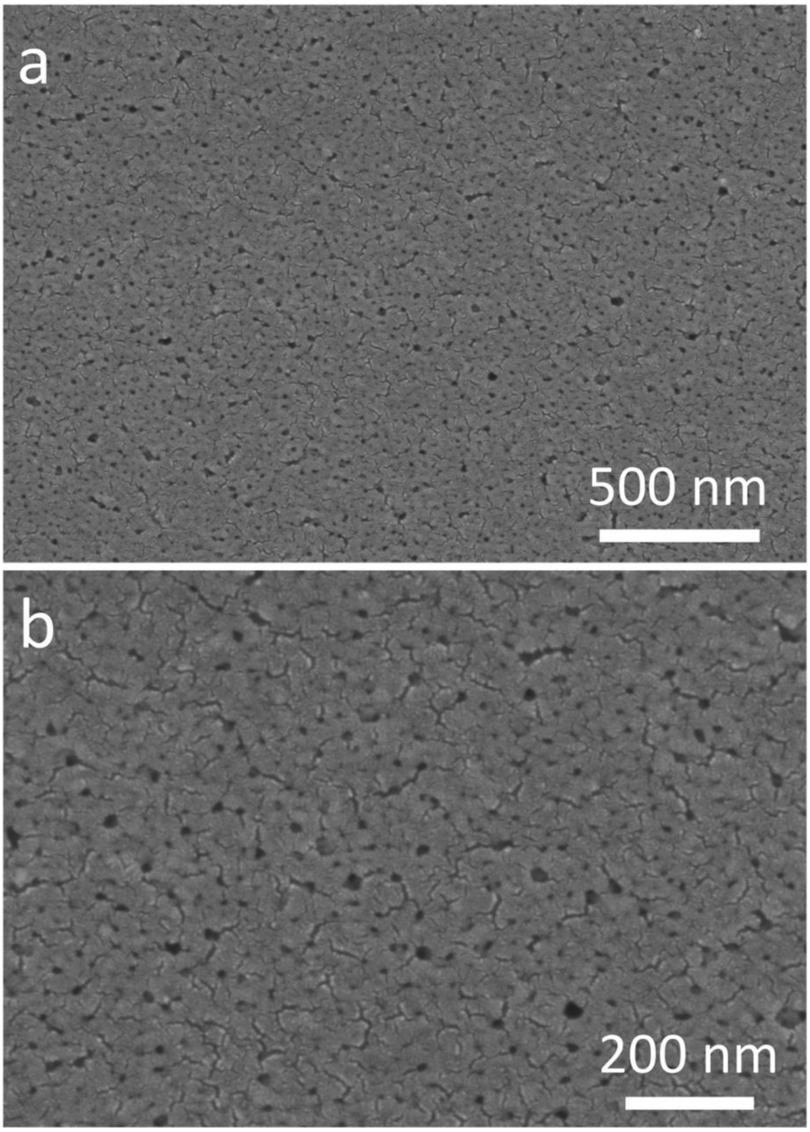


**Supplementary Figure 21.** Surface morphology SEM images of the pristine PSF support.

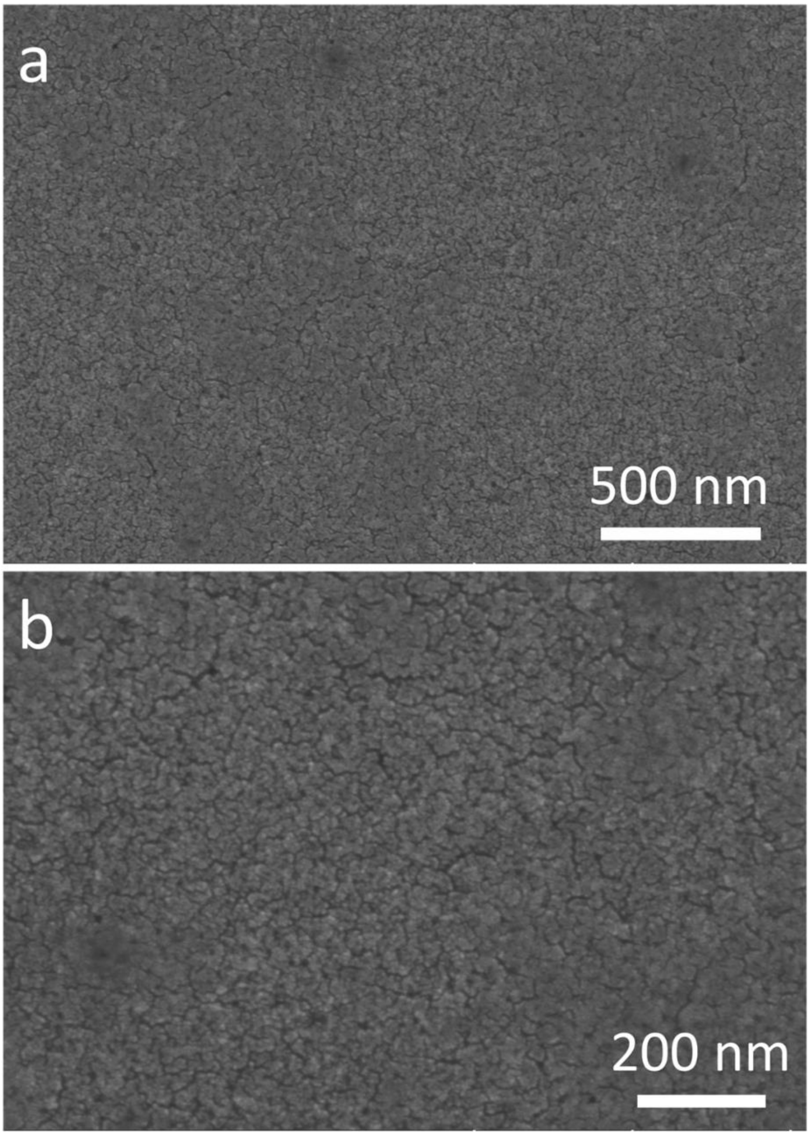


**Supplementary Figure 22.** Surface morphology SEM images of the PSF-G4D-1 support.

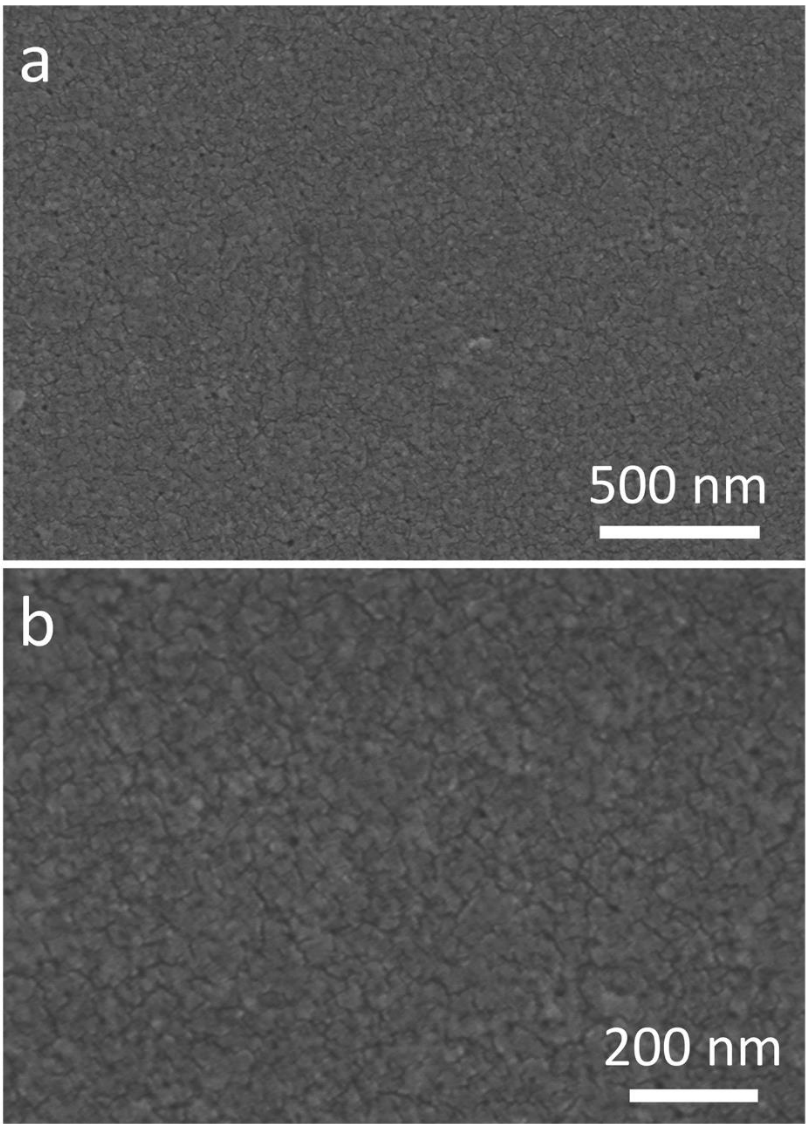


**Supplementary Figure 23.** Surface morphology SEM images of the PSF-G4D-2 support.

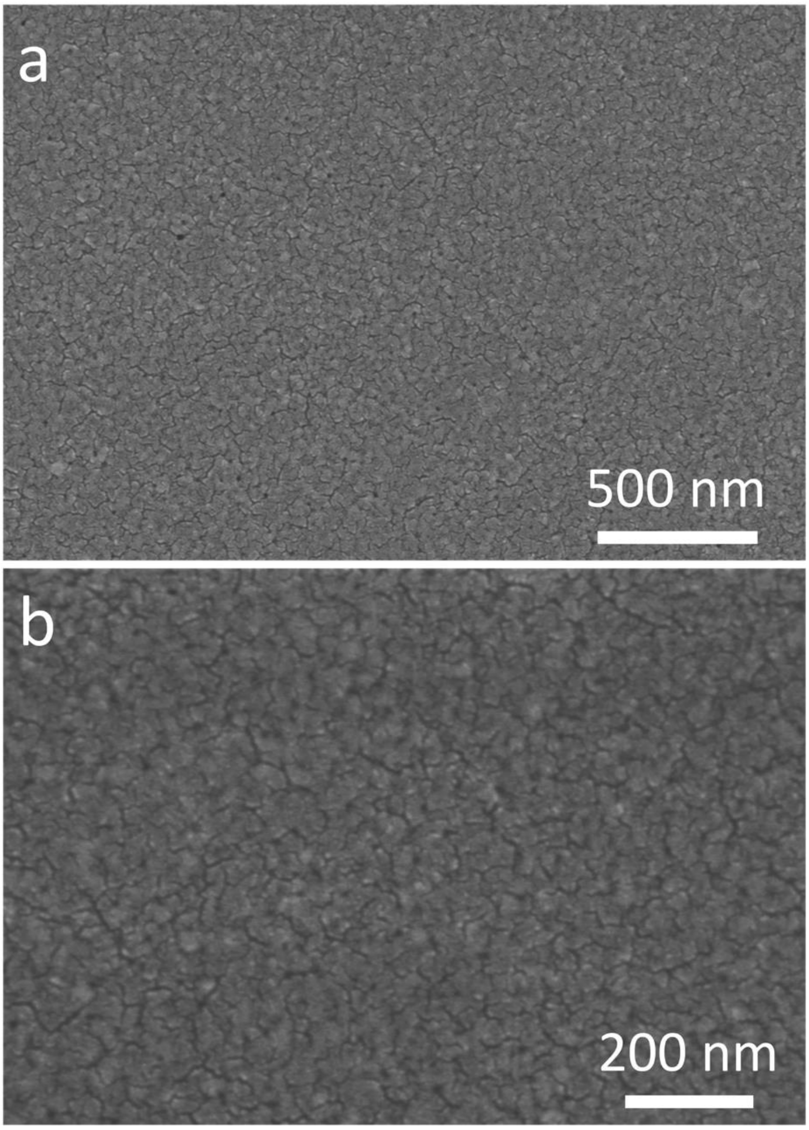


**Supplementary Figure 24.** Surface morphology SEM images of the PSF-G4D-3 support.

As shown in Supplementary Figure 21, Figure 22, Figure 23 and Figure 24, with the modification of polyamide dendrimer porous layer, the pore size of the pristine PSF support was decreased and gradually became even, which is conducive to the uniform distribution of the aqueous amine solution during the IP process and form a defect-free polyamide dense layer.

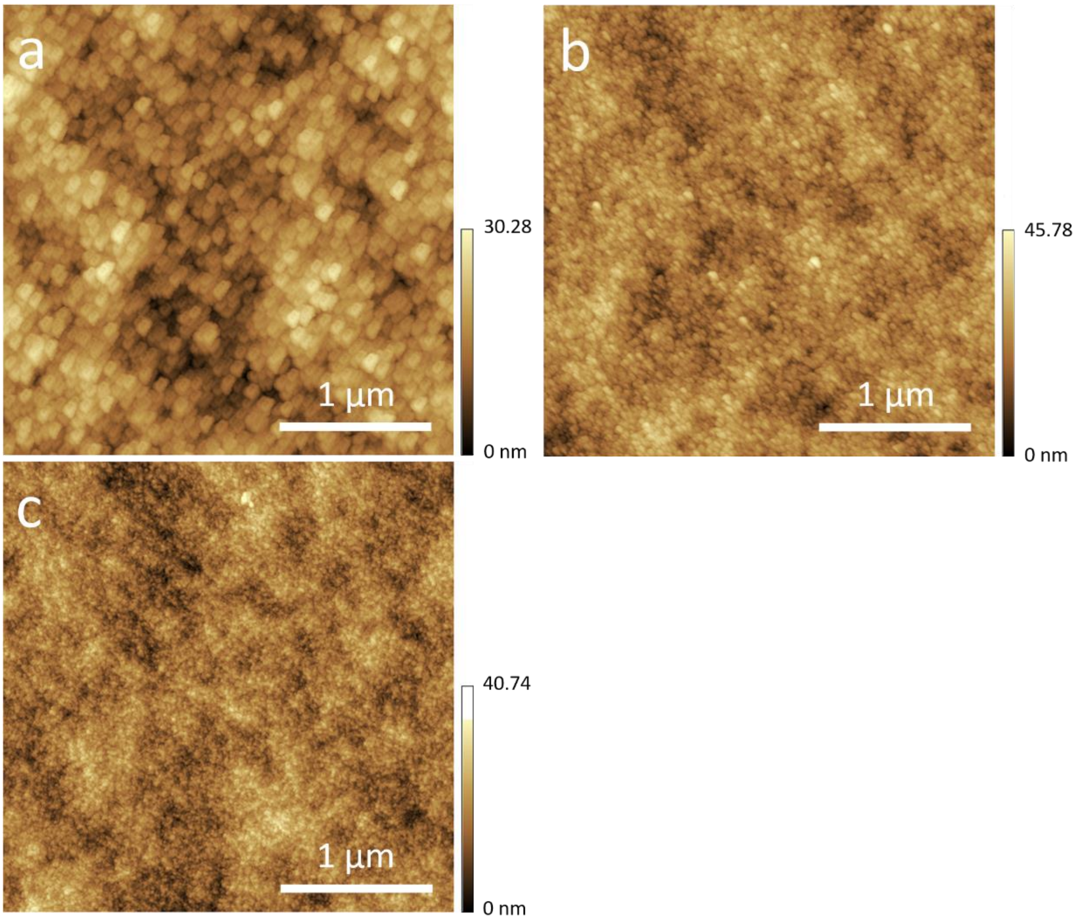


**Supplementary Figure 25.** AFM images of the PSF-G4D-1, PSF-G4D-2 and PSF-G4D-3 supports.

As shown in Supplementary Figure 25, with the increase of the layer number of the dendrimer porous layer, because the pore size of the pristine PSF support became even and small, the size of the dendrimer nanoparticles formed by diazotization coupling reaction gradually decreased.

**Supplementary Table 2.** Liquids used for the detection surface energy parameters (mJ $m^{-2}$).

| Detected liquid | $\gamma_L$ | $\gamma_L^d$ | $\gamma_L^p$ |
|---|---|---|---|
| Water | 72.8 | 21.8 | 51 |
| Diiodomethane | 50.8 | 50.8 | 0 |

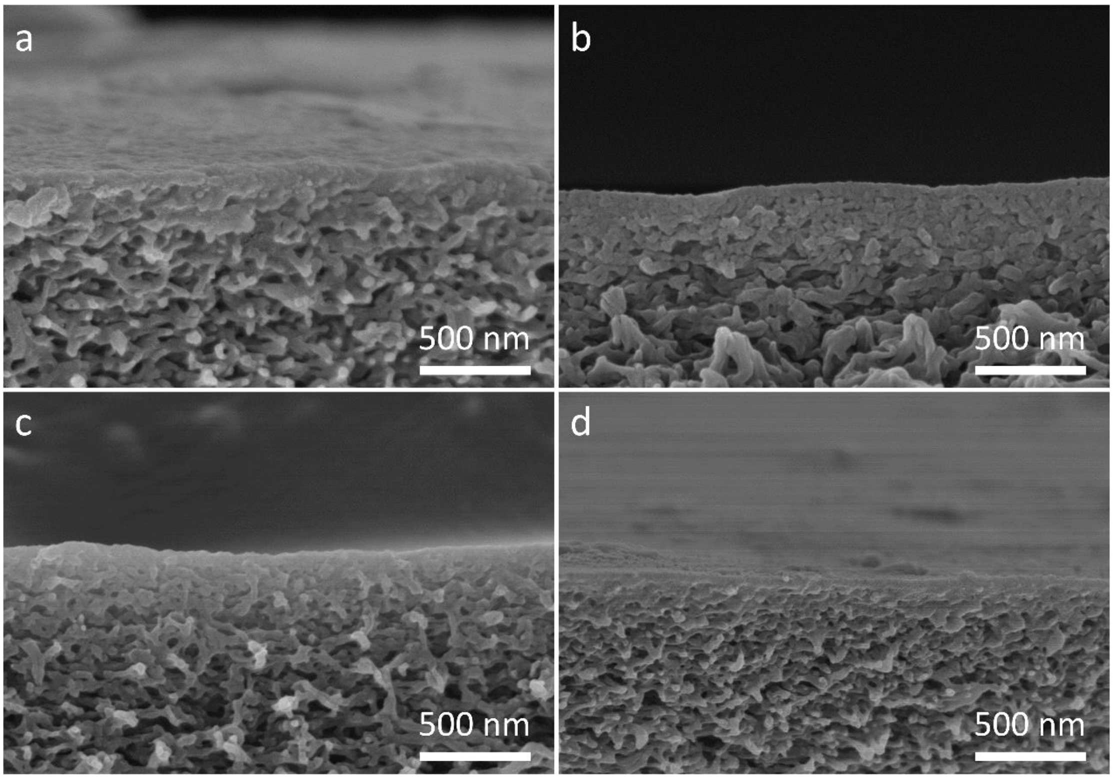


**Supplementary Figure 26.** Cross-sectional morphology SEM images of the PSF (a), PSF-G4D-1 (b), PSF-G4D-2 (c) and PSF-G4D-3 (d) supports.

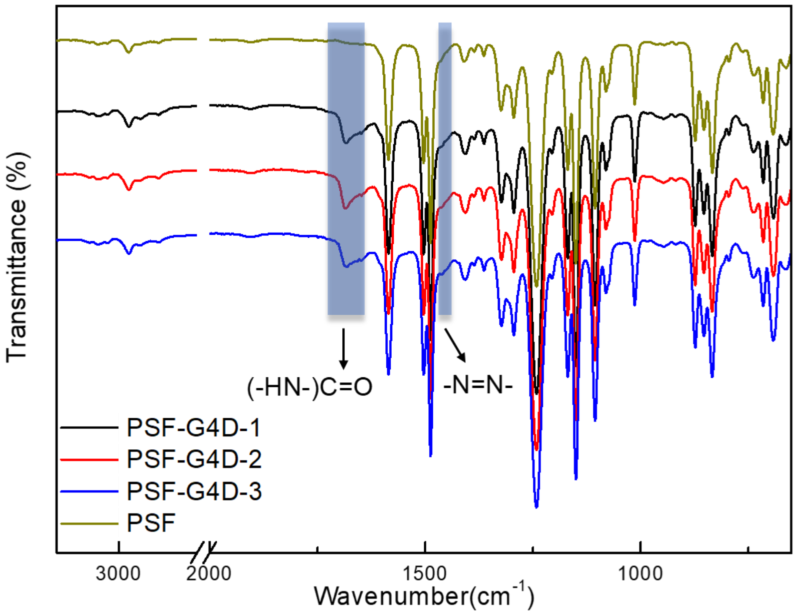


**Supplementary Figure 27.** ATR-FTIR spectrum of the PSF, PSF-G4D-1, PSF-G4D-2 and PSF-G4D-3 supports.

As shown in Supplementary Figure 27, the absorption peak of the carbonyl group in the amide bond is at 1684 $cm^{-1}$, and the infrared absorption peak of -N = N- between dendrimers is at 140 $cm^{-1}$ after the diazotization coupling reaction. This result indicates that the dendrimer porous layer can be deposited on the original PSF support via the diazotization coupling reaction.

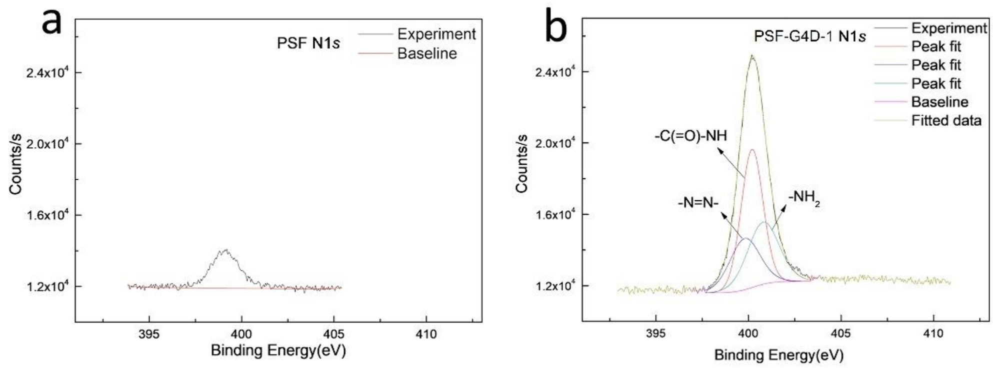


**Supplementary Figure 28.** N1*s* Scan results of the X-ray photoelectron spectra of the pristine PSF support and PSF-G4D-1 support.

As shown in Supplementary Figure 28, compared with the pristine PSF support, the N1*s* scan result of the XPS of the PSF-G4D-1 support shows that there are three types of functional groups, such as amide bonds, diazo bonds, and amine groups. This result indicates that the G4D dendrimer can form the dendrimer porous layer via the diazotization coupling reaction. The amine groups on the dendrimer porous layer is derived from the not participating in the diazotization reaction due to steric hindrance.

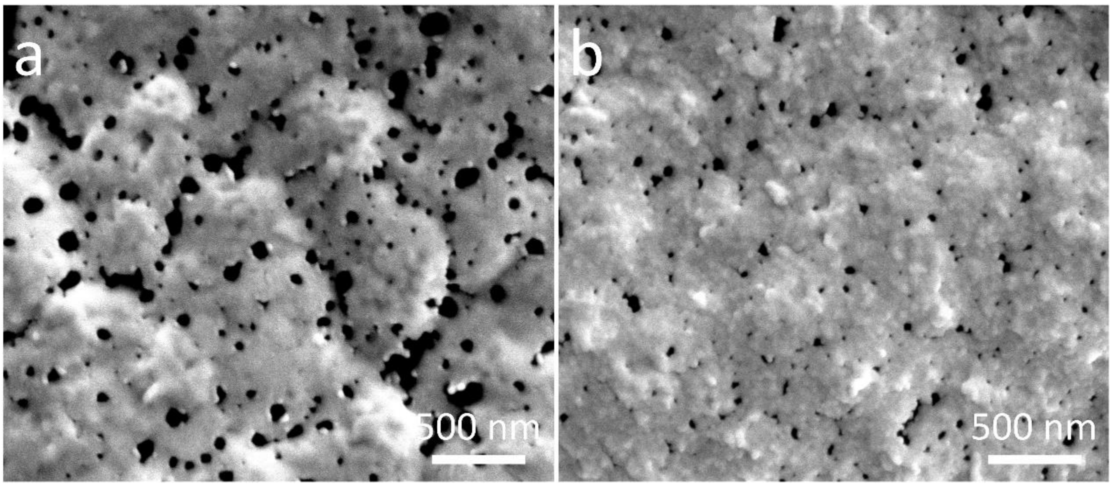


**Supplementary Figure 29.** Surface morphology SEM images of the pristine PVDF support (a) and the PVDF-G4D support (b).

The surface morphology of the pristine PVDF support and the PVDF-G4D support demonstrate that the dendrimer porous layer can repair membrane surface defects sites and uniform the pore size.

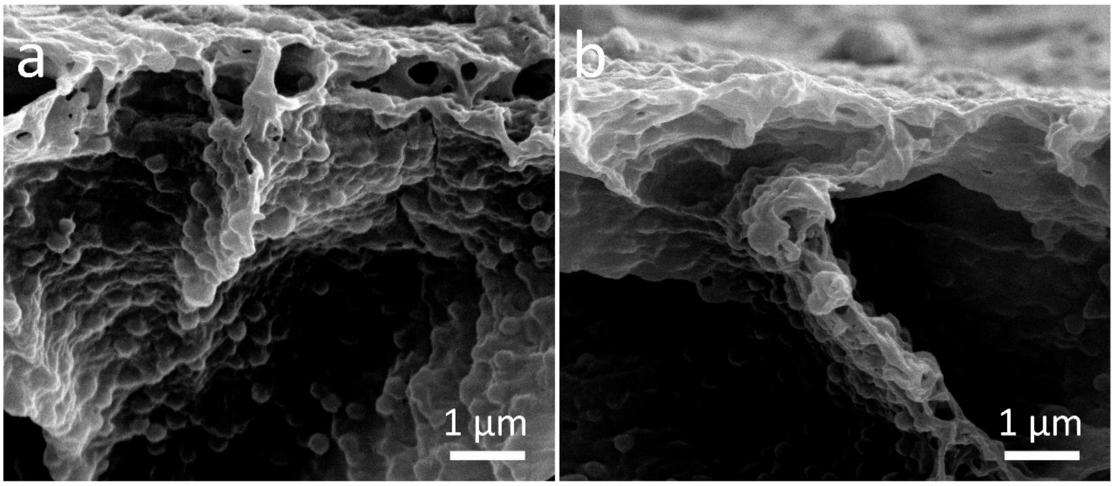


**Supplementary Figure 30.** Cross-sectional morphology SEM images of the pristine PVDF support (a) and the PVDF-G4D support (b).

The cross-sectional morphology of the pristine PVDF support and the PVDF-G4D support have no significant variation.

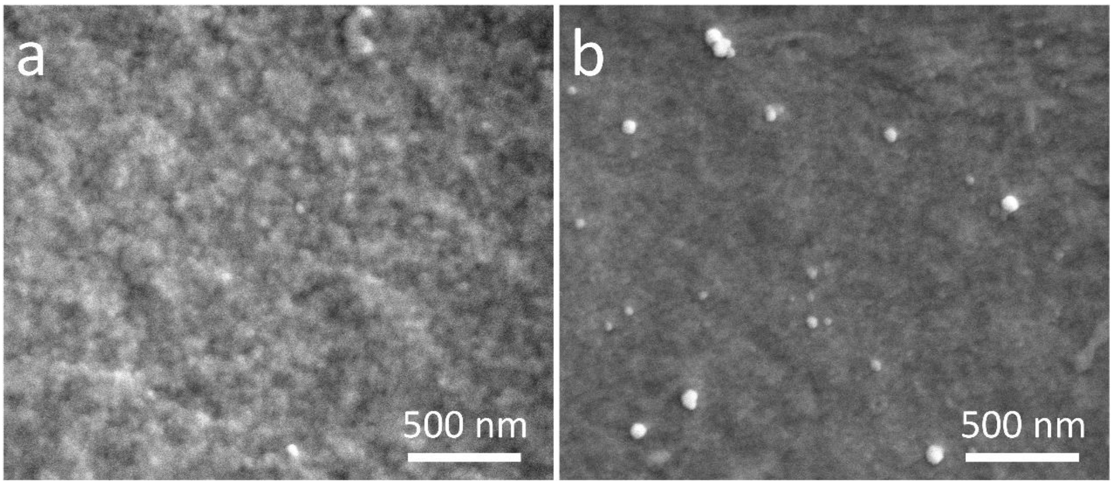


**Supplementary Figure 31.** Surface morphology SEM images of the pristine PAN support (a) and the PAN-G4D support (b).

As shown in Supplementary Figure 31, because the pore size of the pristine PAN support is small and narrow, the dendrimer porous layer has no significant effect on the surface morphology of the PAN support.

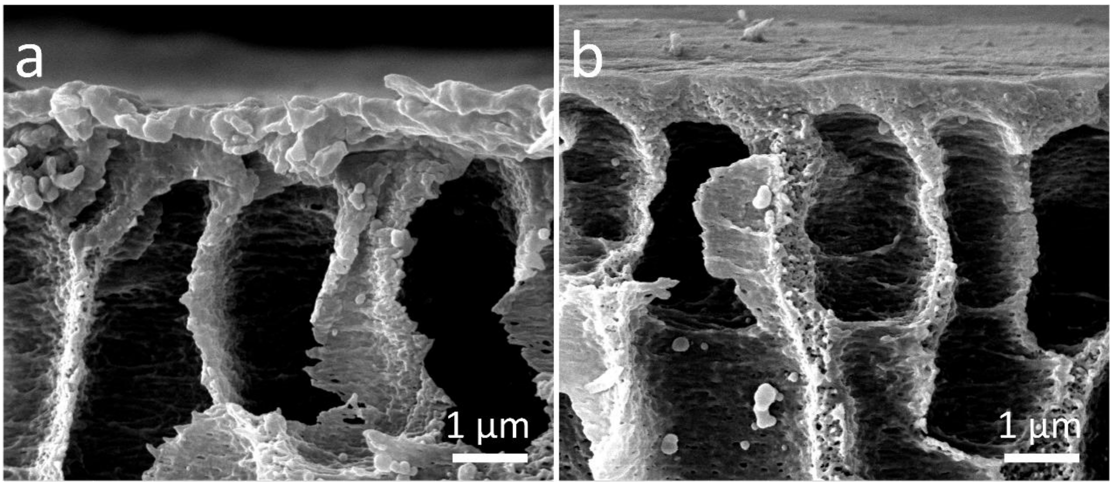


**Supplementary Figure 32.** Cross-sectional morphology SEM images of the pristine PAN support (a) and the PAN-G4D support (b).

The Cross-sectional morphology of the pristine PAN support and the PAN-G4D support have no significant variation.

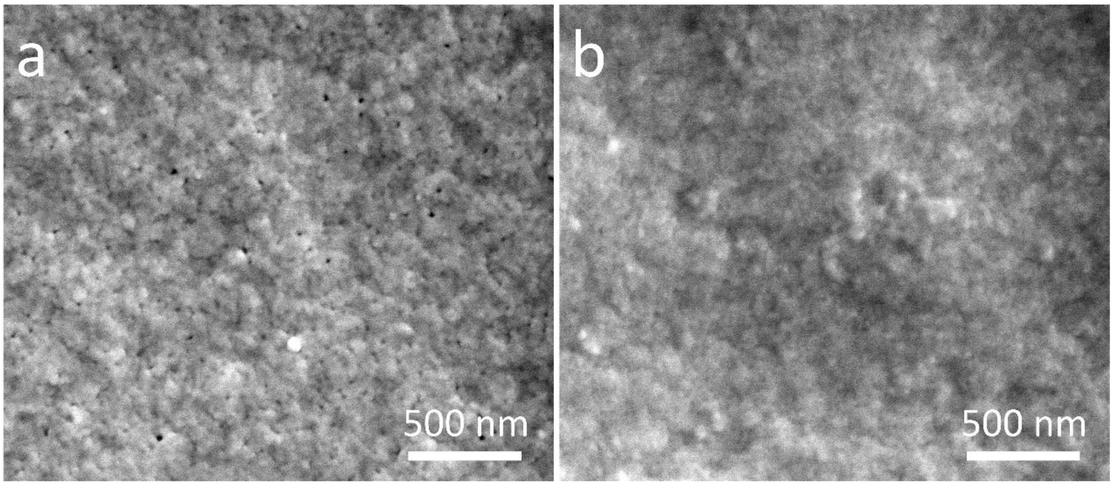


**Supplementary Figure 33.** Surface morphology SEM images of the pristine PI support (a) and the PI-G4D support (b).

As shown in Supplementary Figure 33, after the formation of the dendrimer porous layer, the pore size of the PI-G4D become small and even.

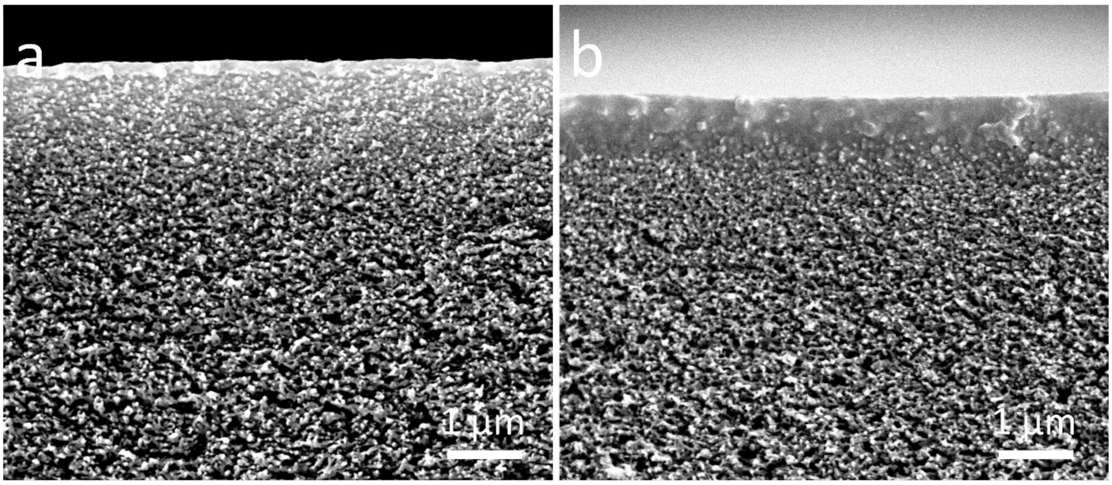


**Supplementary Figure 34.** Cross-sectional morphology SEM images of the pristine PI support (a) and the PI-G4D support (b).

The cross-sectional morphology of the pristine PI support and the PI-G4D support have no significant variation.

### 3.5. Structure and morphology of the traditional and asymmetric polyamide membranes (made by TMC–PIP).

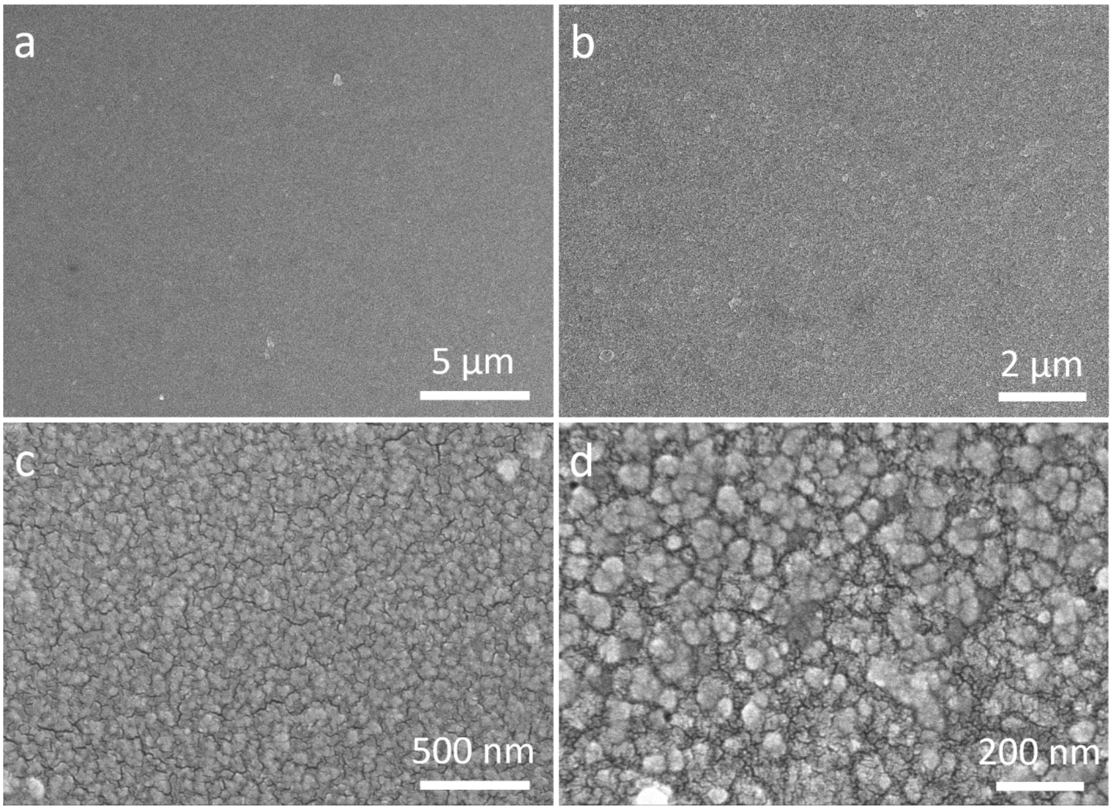


**Supplementary Figure 35.** Surface morphology SEM images of the traditional IP polyamide nanofilm formed on the pristine PSF support. Fabrication condition：reaction time 60s.

As shown in Supplementary Figure 35, the traditional IP polyamide nanofilm formed on the pristine PSF support exhibits the typical nodule structure.

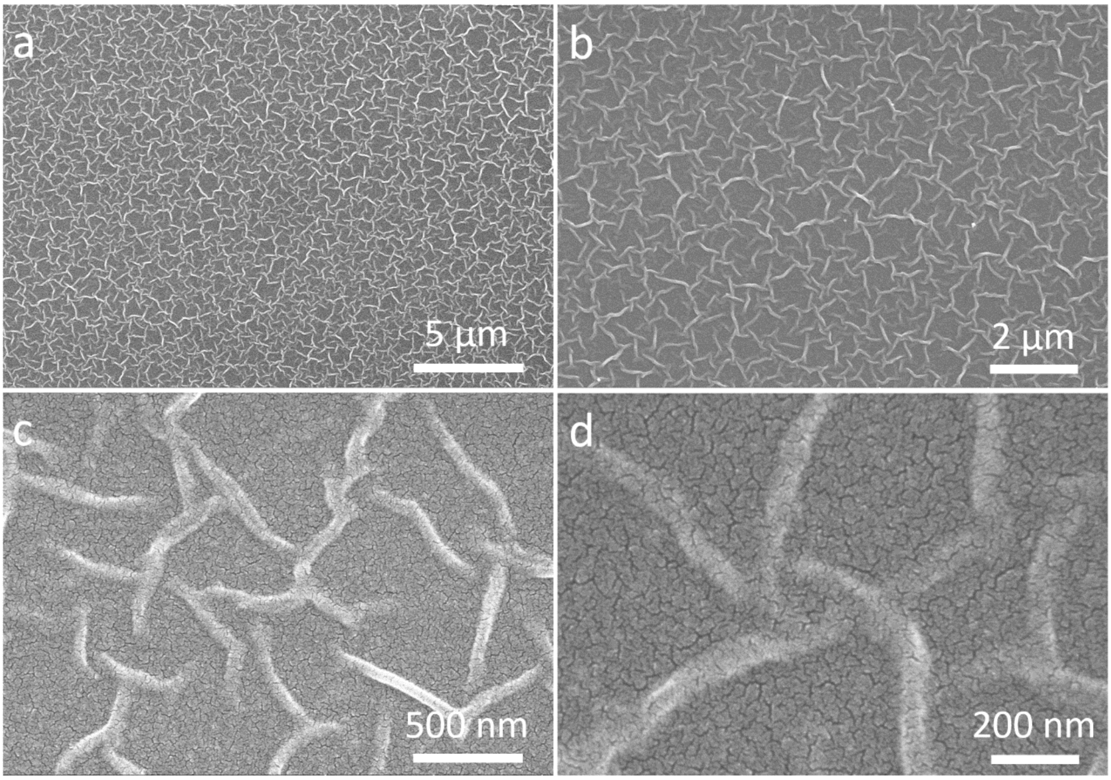


**Supplementary Figure 36.** Surface morphology SEM images of the asymmetric IP polyamide nanofilm formed on the PSF-G4D-1 support. Fabrication condition: reaction time 60s.

As shown in Supplementary Figure 36, the asymmetric IP polyamide nanofilm formed on the PSF-G4D-1 support exhibits the uniform and nano-stripe structure, which is significant different from the traditional IP polyamide nanofilm.

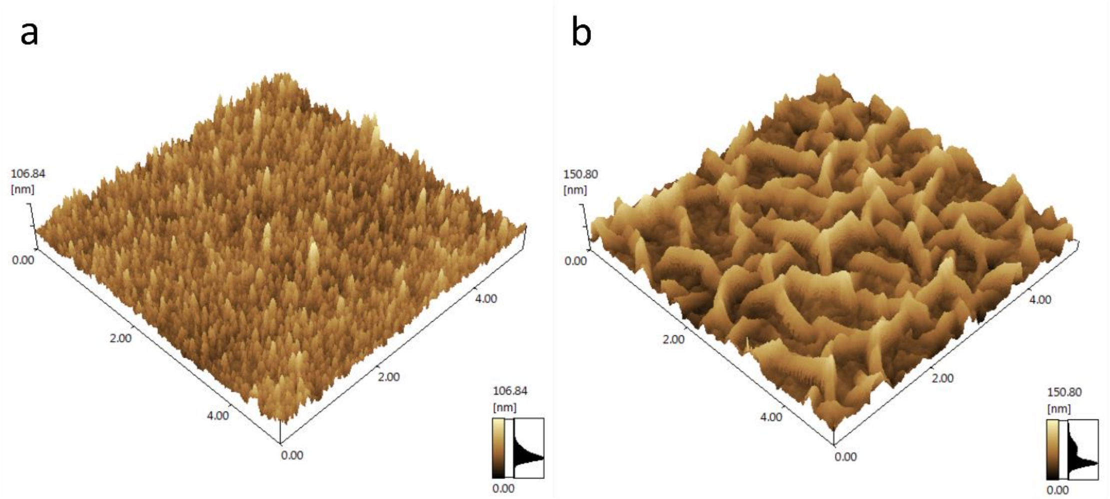


**Supplementary Figure 37.** Surface morphology AFM images (3D) of the traditional and asymmetric IP PA nanofilm. Fabrication condition： reaction time 60s.

As shown in Supplementary Figure 37, the AFM images (3D) shows that the surface morphology of the asymmetric IP polyamide nanofilm and the traditional IP polyamide nanofilm exhibit two significantly different nano-structure, of which the former is the typical nodule structure, and the latter is the novel ordered nano-stripe structure.

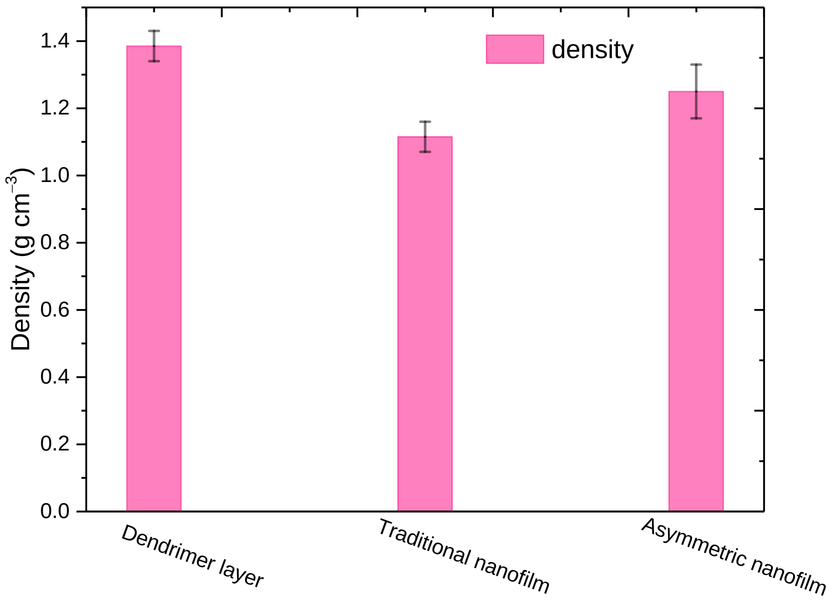


**Supplementary Figure 38.** Volumetric mass densities calculated for the polyamide active layers of the three membranes studied using the areal masses obtained from QCM analyses and thicknesses from ellipsometry analyses.

According to your comments, densities of polyamide dendrimer porous layer, traditional polyamide nanofilm and asymmetric polyamide nanofilm were measured and calculated by the ellipsometry (J. A. Woollam Co., Lincoln, NE) and QCM (Q-Sense, Explorer, Biolin Scientific). Ellipsometry spectra were obtained with a J. A. Woollam Variable angle spectroscopic ellipsometer. We first used the floating method to isolate and deposit the polyamide dendrimer porous layer, traditional polyamide nanofilm and asymmetric polyamide nanofilm onto the QCM sensors, respectively, and then analyzed the change in the frequency of vibration of QCM sensors to obtain the surface density of the samples. After that, we transferred the QCM sensors having deposited polyamide layer onto the ellipsometry platform to conduct the thickness measurement. Finally, by dividing the areal density by the thickness of the polyamide layer, we can calculate the layer density. In an effort to accurate, five locations on each sample were analyzed.

As shown in Supplementary Figure 38, layer densities ranged from 1.12±0.05 g cm$^{-3}$ (traditional polyamide nanofilm) to 1.39±0.05 g cm$^{-3}$ (dendrimer porous layer), and

the middle density value belongs to the asymmetric polyamide nanofilm (1.25±0.08 g $cm^{-3}$). It is well known that layer density is related with the chemical composition[3], dendrimer porous layer consists of fully-aromatic polyamide, and the traditional polyamide nanofilm is semi-aromatic polyamide made by TMC–PIP while for the asymmetric polyamide nanofilm, the bottom layer is full-aromatic polyamide and the upper layer is semi-aromatic polyamide made by TMC–PIP. Hence, the polyamide dendrimer porous layer exhibits the largest volumetric mass density value among three samples.

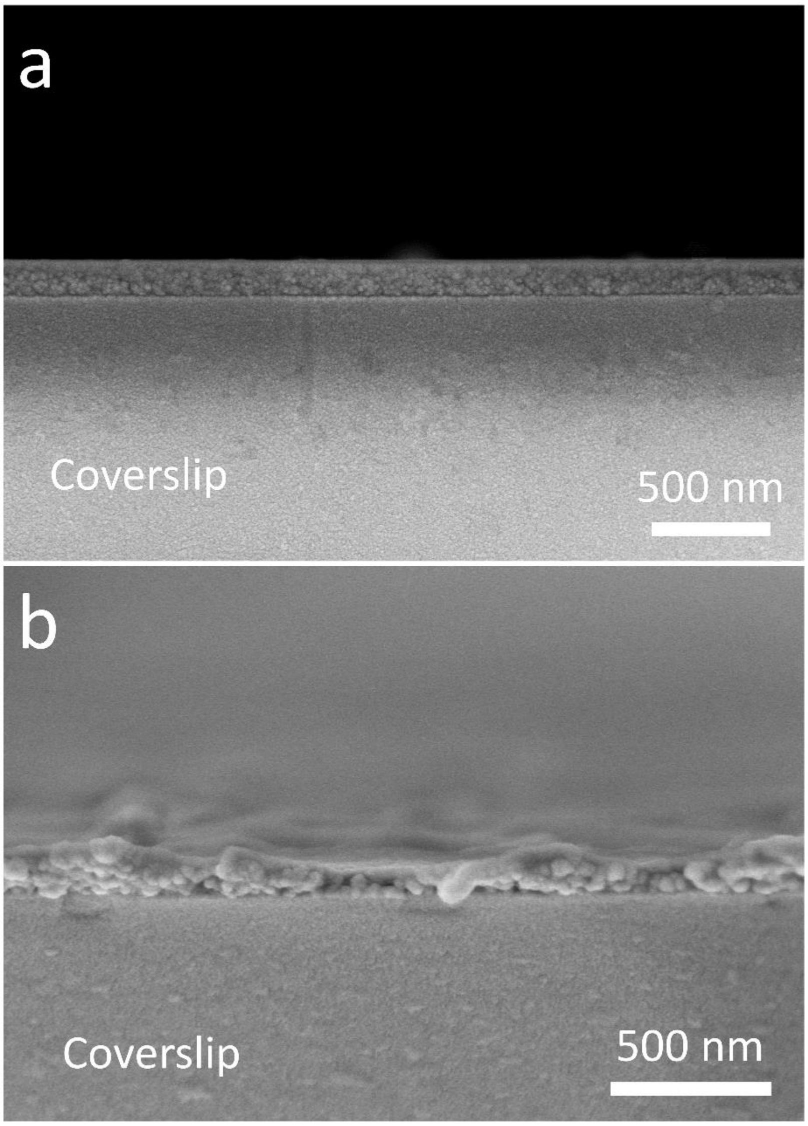


**Supplementary Figure 39.** Cross-sectional morphology of the traditional and asymmetric PA nanofilm. Fabrication condition：reaction time 60s.

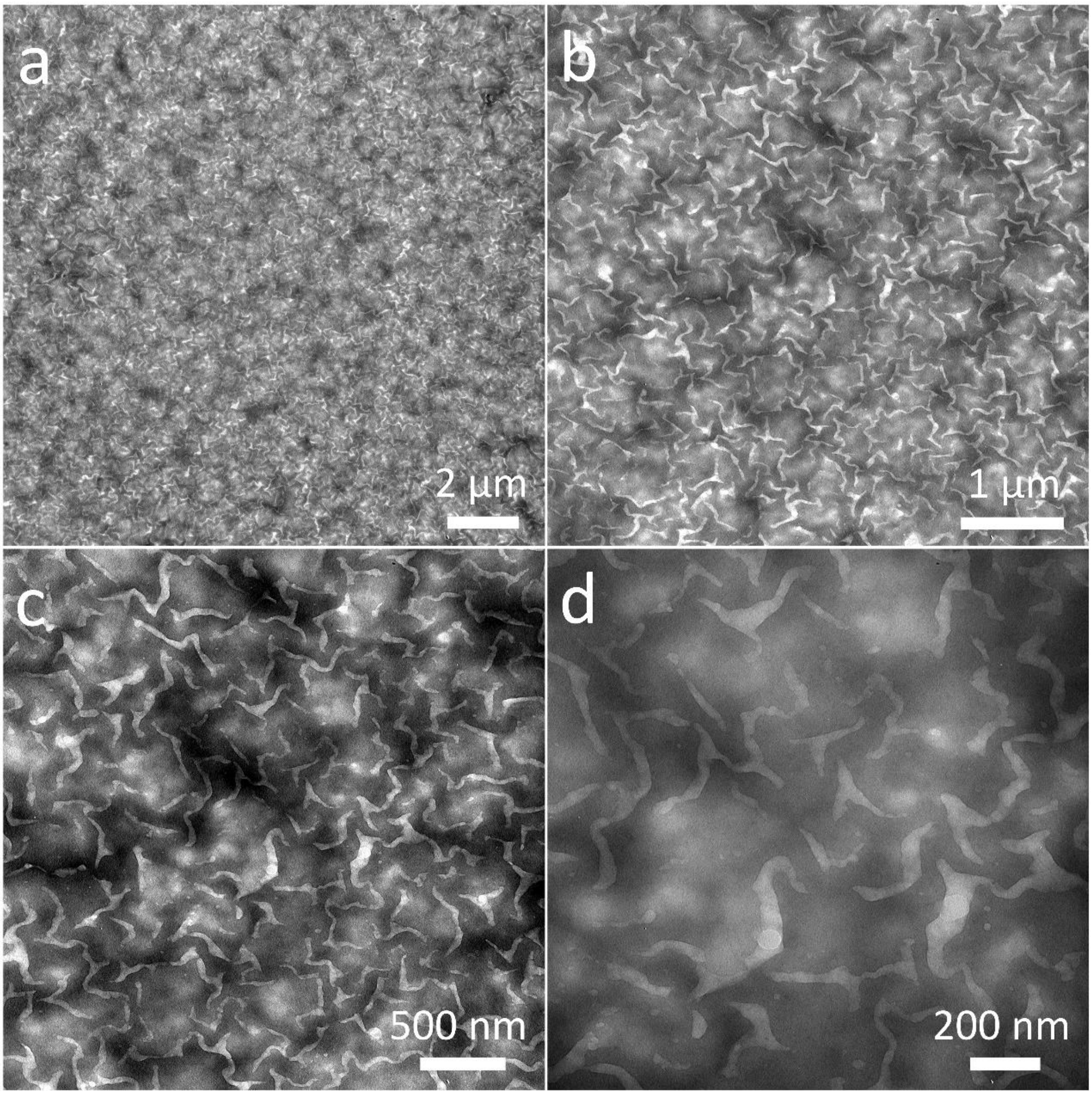


**Supplementary Figure 40.** TEM images of the asymmetric PA nanofilm. Fabrication condition：reaction time 60s.

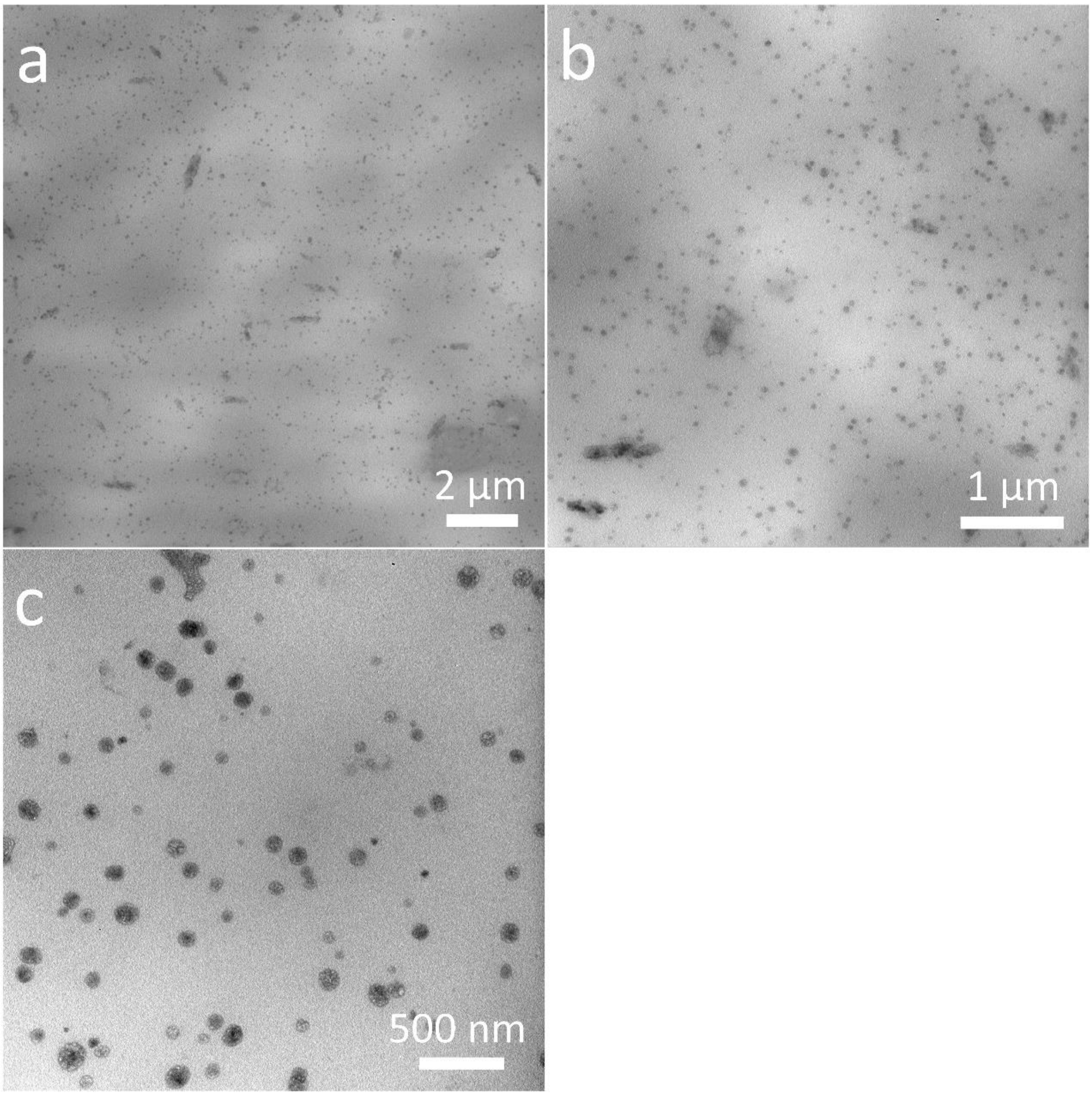


**Supplementary Figure 41.** TEM images of the traditional PA nanofilm. Fabrication condition： reaction time 60s.

**Supplementary Table 3**. Surface properties of the traditional and asymmetric PA membranes. Comparison of the surface properties of the nodule/nano-stripe structures. These results were acquired from AFM measurements over a scanning area of 5 μm by 5 μm. Reported are the averages and standard deviations.

| Sample | AFM Scans | Height (nm) | Rq | Nodule/Nano-stripe width (nm) |
|---|---|---|---|---|
| Traditional PA nanofilm | 256 | 36.11±3.82 | 10.92 | ~100 nm |
| Asymmetric PA nanofilm | 256 | 65.54±2.73 | 22.7 | ~200 nm |

**Supplementary Table 4.** The atomic composition was assessed by XPS measurements from PSF, PSF-G4D-1, traditional PA nanofilm supported on PSF, asymmetric PA nanofilm supported on PSF-G4D-1.

| Membrane | C (%) | N (%) | O (%) | O/N ratio |
|---|---|---|---|---|
| PSF | 83.72 | 1.14 | 15.14 | 13.28 |
| PSF-G4D-1 | 75.85 | 5.99 | 18.16 | 3.03 |
| Traditional PA nanofilm | 77.9 | 8.56 | 13.54 | 1.58 |
| Asymmetric PA nanofilm | 72.87 | 11.88 | 15.25 | 1.28 |

The atomic composition assessed by XPS measurements shows that due to the formation of dendrimer porous layer, the N content of the PSF-G4D-1 support is higher than that of the pristine PSF support. In addition, based on its lower O/N ratio, the crosslinking degree of the asymmetric PA nanofilm (63.2%) is higher than that of the traditional PA nanofilm (32.6%), which is conducive to enhance the rejection of salt.

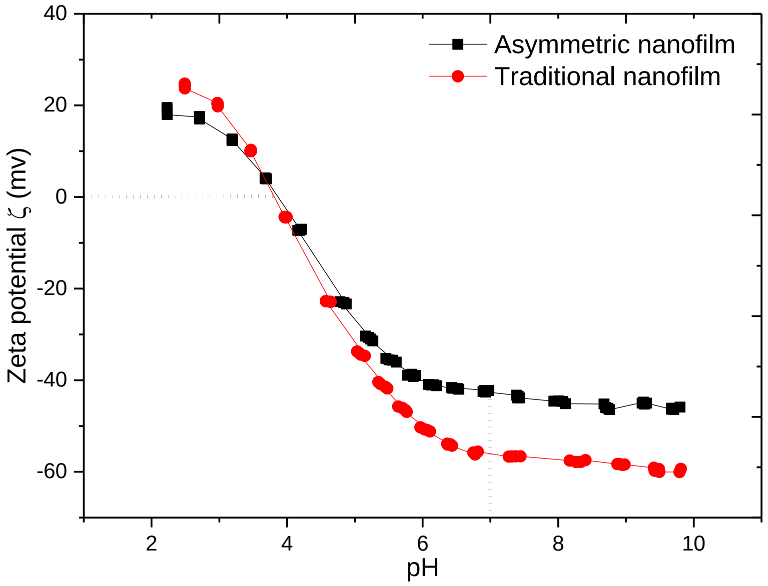


**Supplementary Figure 42.** Representative ζ potential of the fabricated traditional and asymmetric polyamide nanofilms. The membrane zeta potentials were estimated by measuring the ζ potentials in a background electrolyte solution of 1 mM KCl. All measurements were performed at 25°C and repeated 4 times.

**Supplementary Table 5.** Hydrated radius and diffusion coefficient of divalent and monovalent ions[4].

| Ion | Ionic radius (nm) | Hydrated radius (nm) | Diffusion coefficient ($10^{-9}$ $m^2$ $s^{-1}$) |
|---|---|---|---|
| $Na^+$ | 0.095 | 0.358 | 1.334 |
| $Ca^{2+}$ | 0.099 | 0.412 | 0.792 |
| $Mg^{2+}$ | 0.065 | 0.428 | 0.706 |
| $Cl^-$ | 0.181 | 0.332 | 2.032 |
| $SO_4^{2-}$ | 0.290 | 0.379 | 1.065 |

As shown in Supplementary Figure 42, the isoelectric point (IEP) of the asymmetric polyamide nanofilms is lower than that of the traditional polyamide nanofilm, of which the former is 3.81, and the latter is 3.88. The surface zeta potential value of the asymmetric nanofilm is thus less negative than that of the traditional nanofilm in the range of pH 3.8 to 10. For example, the related zeta potential value at pH=7 is -55.9 mV for traditional nanofilm and -42.7 mV for the asymmetric nanofilm, respectively.

From the XPS results, the lower −COOH content (enhanced crosslinking degree) in the semi-aromatic polyamide nanofilms is mainly responsible for the decreased surface negative charge. Generally, charge exclusion and size exclusion contribute significantly to the separation performance of the fabricated IP polyamide membranes[5,6]. For the original semi-aromatic polyamide membranes with surface negative charge, sulfate rejection was always greater than that of chlorinated salt. This high sulfate rejection can be explained by the high negative surface charge of the original polyamide membranes. In the case of anions, $SO_4^{2-}$ with a valence of -2 will experience greater electronegative repulsion from the negatively charged membrane surface as opposed to $Cl^-$. In addition, $SO_4^{2-}$ has larger hydrated radius and lower ionic diffusivity than $Cl^-$ (Supplementary Table 5). On the other hand, thanks to the crosslinking degree of polyamide nanofilm, the $Na_2SO_4$, $MgSO_4$ and NaCl rejection rates of the asymmetric polyamide membrane are increased compared to the traditional polyamide membranes. For example, the $Na_2SO_4$, $MgSO_4$ and NaCl rejection rates of the asymmetric polyamide membrane are increased from 96% to 99.2%, 93.4% to 99.1% and 33.7% to 41.1%, respectively.

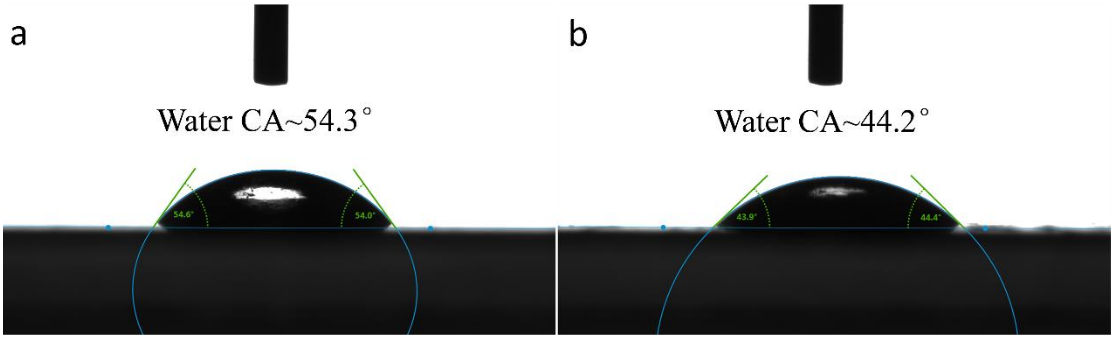


**Supplementary Figure 43.** Water contact angle (CA) on the surface of (a) traditional PA nanofilm, (b) asymmetric PA nanofilm.

As shown in supplementary Figure 43, water contact angle (CA) shows that the hydrophilicity of asymmetric polyamide nanofilm is better than traditional polyamide nanofilm due to the ordered nano-stripe structure and the enhancement of the crosslinking degree.

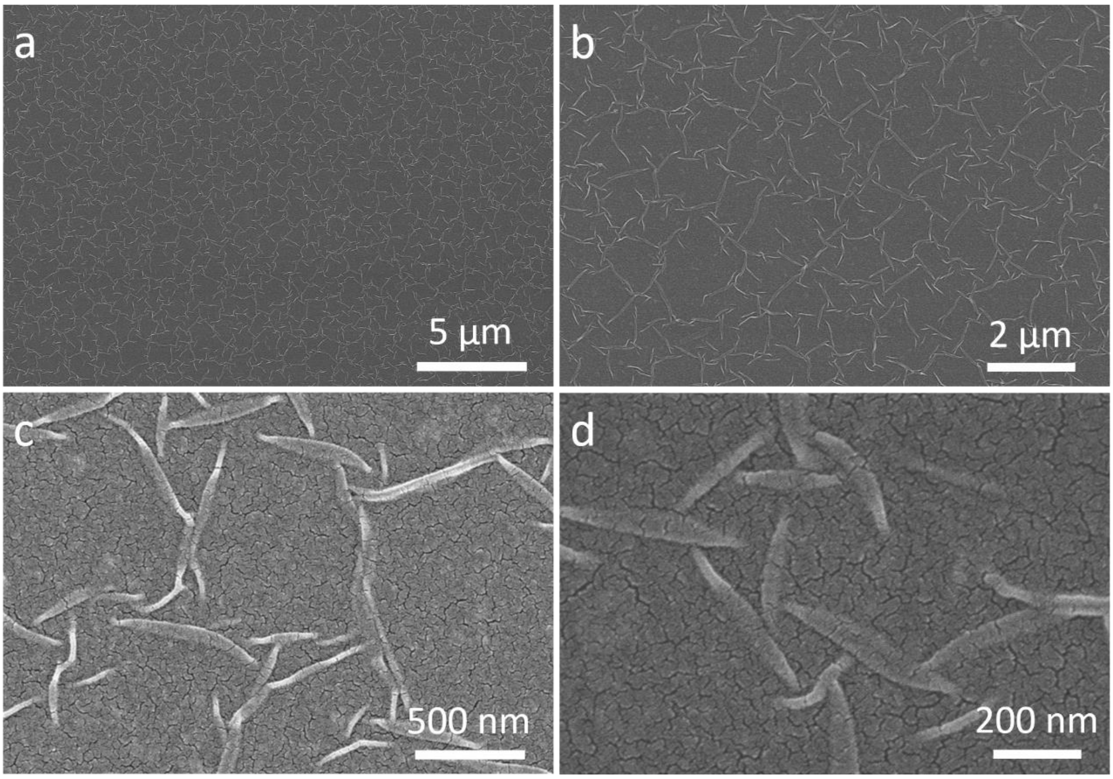


**Supplementary Figure 44.** Surface morphology SEM images of the asymmetric IP polyamide nanofilm formed on the PSF-G4D-1 support. Fabrication condition: reaction time 30s.

As shown in Supplementary Figure 44, the density and height of the nano-stripe structure on the asymmetric polyamide nanofilm is decreased with the decrease of the IP time. This result indicates that the nano-stripe structure can be tuned via controlling the IP time, and further controlling the membrane performance.

## 3.6. Structure, morphology and desalination performance of the traditional and asymmetric polyamide membranes (made by TMC–MPD).

**Supplementary Table 6.** The atomic composition was assessed by XPS measurements from traditional polyamide (PA) nanofilm（TMC–MPD）supported on PSF substrate, asymmetric PA nanofilm（TMC–MPD）supported on PSF-G4D-1 substrate.

| Membrane | C (%) | N (%) | O (%) | O/N ratio |
|---|---|---|---|---|
| Traditional PA nanofilm (T-TMC–MPD) | 71.48 | 12.14 | 16.38 | 1.35 |
| Asymmetric PA nanofilm (A-TMC–MPD) | 69.89 | 11.26 | 18.85 | 1.67 |

We named traditional polyamide nanofilm made by TMC–MPD as T-TMC–MPD, representing the single layer polyamide RO membrane, and named asymmetric polyamide nanofilm made by TMC–MPD as A-TMC–MPD, representing the two-layer polyamide RO membrane, in which the upper is the dense polyamide layer and the lower is the dendrimer porous layer. The atomic composition assessed by XPS measurements shows that the A-TMC–MPD polyamide nanofilm exhibits a higher O/N ratio than that of the T-TMC–MPD polyamide nanofilm, which indicates that A-TMC–MPD nanofilm surface exhibits a higher –COOH content. Based on O/N ratio, we calculated that the surface degree of network cross-linking (DNC) of T-TMC–MPD and A-TMC–MPD nanofilms were 55%, 24.7%, respectively.

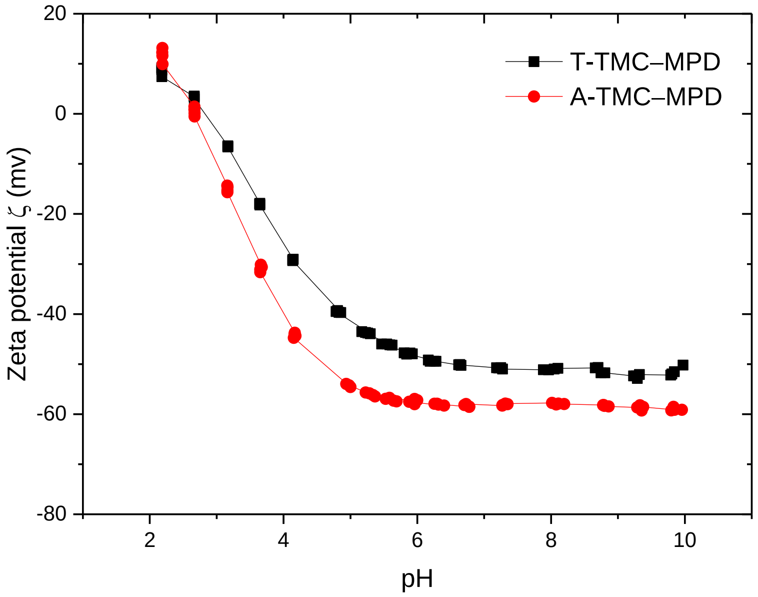


**Supplementary Figure 45**. Representative zeta (ζ) potential of the fabricated T-TMC–MPD and A-TMC–MPD polyamide membranes. The membrane ζ potentials were estimated by measuring the ζ potentials in a background electrolyte solution of 1 mM KCl. All measurements were performed at 25°C and repeated 4 times.

As shown in Supplementary Figure 45, the isoelectric point (IEP) of the A-TMC–MPD polyamide nanofilms is lower than that of the T-TMC–MPD nanofilm, which the former is 2.69, and the latter is 2.82. The surface zeta potential value of the T-TMC–MPD nanofilm is less negative than that of the A-TMC–MPD nanofilm in the range of pH 2.82 to 10. For example, the related zeta potential value at pH=7 is -50.5 mV for T-TMC−MPD nanofilm and -58.1 mV for A-TMC−MPD nanofilm, respectively. From the above XPS results, the higher −COOH content in the surface of the aromatic polyamide nanofilms is mainly responsible for enhanced surface negative charge.

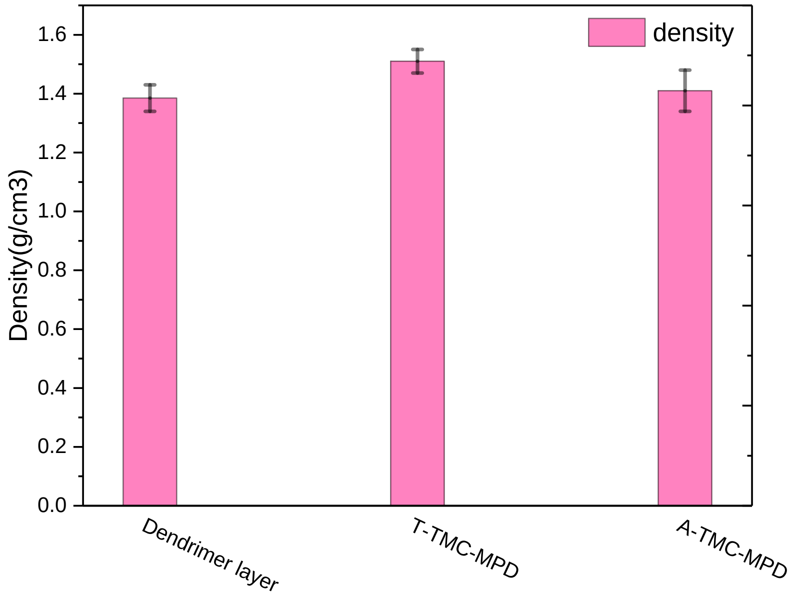


**Supplementary Figure 46.** Volumetric mass densities calculated for the polyamide active layers of the dendrimer porous layer, T-TMC–MPD and A-TMC–MPD membranes studied using the areal masses obtained from QCM analyses and thicknesses from ellipsometry analyses.

The calculated values of layer density for the dendrimer porous layer, T-TMC–MPD and A-TMC–MPD nanofilms fabricated are shown in Supplementary Figure 46. It is well known that layer density is related with the chemical composition. Layer densities ranged from 1.39±0.05 g $cm^{-3}$ (dendrimer porous layer) to 1.51±0.04 g $cm^{-3}$ (T-TMC–MPD), and the middle density value belongs to the A-TMC–MPD (1.41±0.07 g $cm^{-3}$). As described in fabricated process, three layers were all composed of full aromatic polyamide. The dendrimer porous layer is formed by spherical dendrimers through diazotization coupling reaction, which is loose and has no separation performance. The T-TMC–MPD polyamide nanofilm was formed on the PSF support directly, while the A-TMC–MPD polyamide nanofilm was fabricated on the dendrimer porous layer. Hence, the T-TMC–MPD layer density is highest among three types of layers.

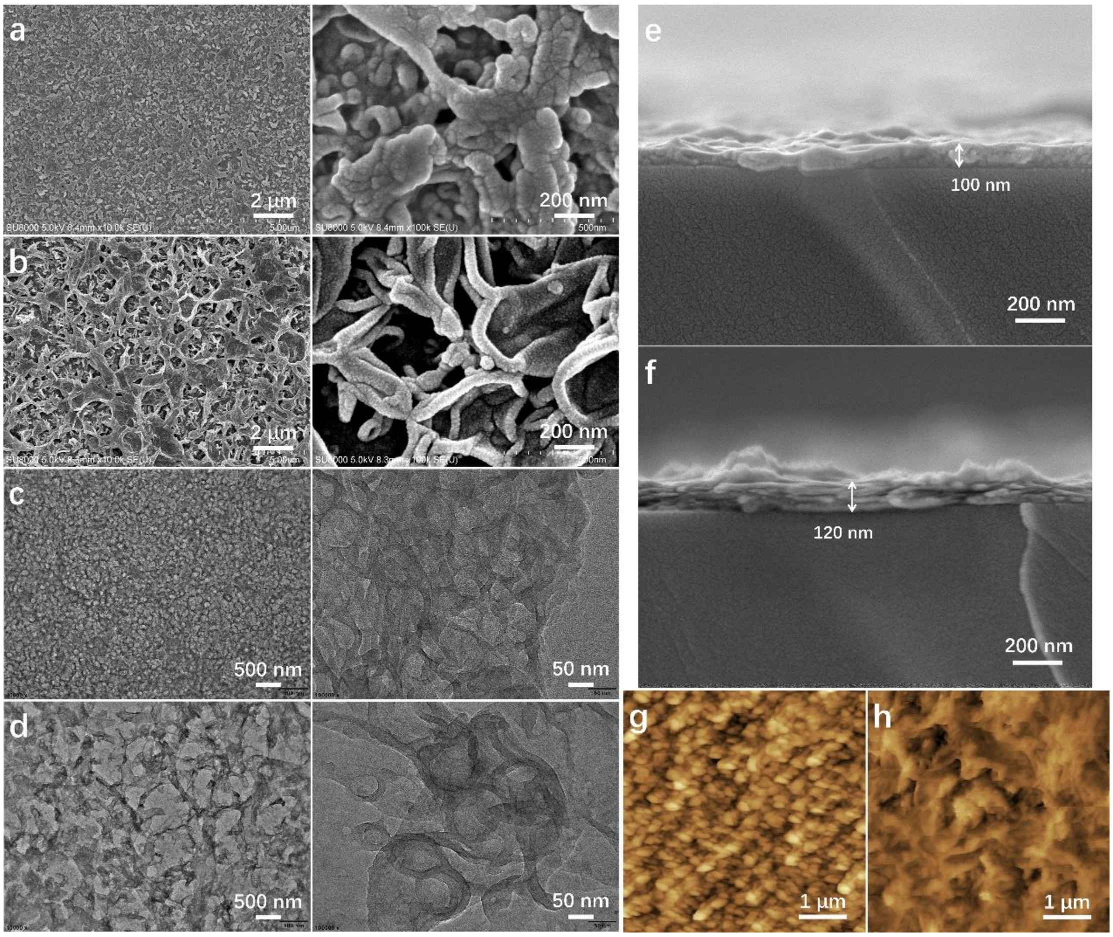


**Supplementary Figure 47. Morphology characterizations.** **a** SEM surface images of the traditional single polyamide nanofilm made by TMC–MPD (T-TMC–MPD). **b** SEM surface images of the asymmetric polyamide nanofilm made by TMC–MPD (A-TMC–MPD). **c** TEM images of the traditional polyamide nanofilm. **d** TEM images of the asymmetric polyamide nanofilm. **e** SEM cross-sectional images of the traditional polyamide nanofilm supported by coverslip. **f** SEM cross-sectional images of the asymmetric polyamide nanofilm supported by coverslip. **g h** AFM surface images of the traditional single polyamide nanofilm and asymmetric polyamide nanofilm made by TMC–MPD.

From SEM and AFM surface images in Supplementary Figure 47, it was found that the single polyamide nanofilm is denser than that of the asymmetric polyamide nanofilm. Further magnification images in Supplementary Figure 47a and 47b indicate that the asymmetric polyamide nanofilm exhibits a visible and large ridge-and-valley structure composed of dense layer, while the ridge-and-valley in the single polyamide

nanofilm is small and densely packed together. TEM images also further confirm that the ridge-and-valley structure in the asymmetric polyamide nanofilm (A-TMC–MPD) is looser and larger than that of the single polyamide nanofilm (T-TMC–MPD). The magnified TEM image in Supplementary Figure 47d demonstrates that the voids in the ridge is larger, and have no over-stacking, which produces a better water transport path. In addition, SEM images in Supplementary Figure 47e and 47f clearly show that the cross-sectional morphology and thickness of the resulted polyamide nanofilms. The single polyamide dense layer is ~100 nm, while that of the asymmetric polyamide nanofilm is ~120 nm. A further observation reveals that the asymmetric polyamide nanofilm has two layers, the upper is the dense layer with a thickness of ~60 nm, and the bottom layer is the dendrimer porous layer with an average thickness of ~60 nm. Overall, the resulted asymmetric polyamide nanofilm has no over-stacking, showing a larger ridge-and-valley structure and thinner dense active layer, which can decrease the mass transfer resistance for water and improve solute separation efficiency.

**Supplementary Table 7. Separation performance of the fabricated polyamide membranes, and comparation with the commercial polyamide RO membrane.** The operating pressure and temperature were controlled at 1.55 MPa, 25°C. The feed flow rate was 7.5 L $min^{-1}$, and the NaCl concentrations of the feed solutions were 2000 ppm.

| Membrane types | Water flux (kg $m^{-2}$ $h^{-1}$) | NaCl rejection (%) |
|---|---|---|
| T-TMC–MPD | 18.39±3.3 | 98.67 ± 0.3 |
| A-TMC–MPD | 52.63±4.2 | 98.65±0.25 |
| BW30[1] | 41.85±2.48 | 97.15±0.45 |
| SW30XLE[2] | 27.25±2.7 | 98.25±0.32 |

It is clearly shown that the A-TMC–MPD polyamide membrane was provides a 2.86 folds higher water flux for 2000 ppm NaCl solutions compared with the T-TMC–MPD polyamide membrane due to the thinner dense layer and larger ridge-and-valley structure. Moreover, compared with the commercial RO membrane, the asymmetric polyamide membrane achieved higher water flux and slightly better rejection than BW 30 and SW30XLE membranes. These results indicate that the asymmetric polyamide membrane provides a novel approach to fabricate superior performance polyamide membrane.

### 3.7. Separation performance of the traditional and asymmetric polyamide membranes.

**Supplementary Table 8.** Separation performance of the asymmetric polyamide membranes supported on the PSF-G4D-1 support, Reaction time: 30s. The operating pressure was controlled at 1 MPa, and the temperature was maintained at 25°C by a heat exchanger. The feed flow rate was 7.5 L min$^{-1}$, and the concentrations of salts in the feed solutions were 2000 ppm. Separation performance measurements were conducted 1 hour after starting the filtration to stabilize the membrane performance. Rejection rates were calculated on the basis of the electrical conductivities of feed and permeate solutions.

| Salt | Water flux (kg m$^{-2}$ h$^{-1}$) | Rejection (%) |
|---|---|---|
| $Na_2SO_4$ | 192.19±4.2 | 99.43 ± 0.21 |
| $MgSO_4$ | 196.51±6.8 | 99.12± 0.26 |
| $CaCl_2$ | 171.47±5.9 | 78.81±1.98 |
| $MgCl_2$ | 188.63±4.8 | 71.39±2.17 |
| NaCl | 223.17±8.5 | 42.48±2.76 |

The separation performance of the asymmetric polyamide membrane (IP reaction time: 30s) is shown in Supplementary Table 8. Separation data showed that the asymmetric polyamide membrane was improved in both flux and rejection compared to the traditional polyamide membrane. The asymmetric polyamide membrane provides a 2.69–3.13 folds higher water flux for various salt solutions with 2000 ppm compared with the traditional polyamide membrane. Moreover, salt rejection rate has also been improved. For example, $MgSO_4$ rejection rate of the asymmetric polyamide membrane was increased from 93.41% to 99.43%, and water flux was increased from 68.51 kg m$^{-2}$ h$^{-1}$ to 196.51 kg m$^{-2}$ h$^{-1}$.

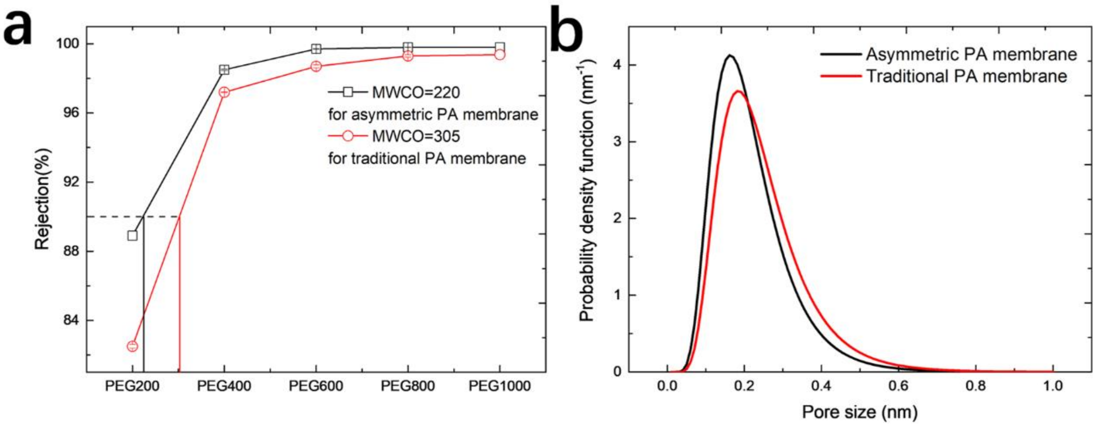


**Supplementary Figure 48.** Rejection curves to PEG with different molecular weight (a) and pore size distribution of the traditional PA and asymmetric PA membranes.

As for the effective pore size, the molecular weight cut-off (MWCO) of the traditional PA and asymmetric PA membranes were determined through permeation tests to the PEG with different molecular weight, such as 200 Da, 400 Da, 600 Da, 800Da and 1000 Da. As shown in Supplementary Figure 48, the MWCO for the asymmetric and traditional polyamide nanofilm is 220 Da, 305 Da, respectively. The pore size distribution curves show that the asymmetric polyamide nanofilm exhibits a decreased mean effective pore radius (0.19 nm) than that of the traditional nanofilm (0.22 nm). These results indicate that the asymmetric polyamide nanofilm exhibits a more efficient size exclusion effect on the ions to be separated, and high salt rejection rate thus can be achieved.

**Supplementary Table 9.** Comparison of commercial membranes and asymmetric polyamide membrane.

| Membrane type | $MgSO_4$ | | $Na_2SO_4$ | | NaCl | |
|---|---|---|---|---|---|---|
| | Rejection (%) | Water flux (kg $m^{-2}$ $h^{-1}$) | Rejection (%) | Water flux (kg $m^{-2}$ $h^{-1}$) | Rejection (%) | Water flux (kg $m^{-2}$ $h^{-1}$) |
| Asymmetric | 99.1 ± 0.3 | 269.9±8.3 | 99.2 ± 0.3 | 264±5.1 | 41.1±1.4 | 306.3±9.7 |
| Traditional | 93.4 ± 1.6 | 68.5±2.8 | 96 ± 1.8 | 71.4±2.4 | 33.7 ± 2.3 | 71.4±3.3 |
| DK | 98.9±0.4 | 68.8±1.6 | 98.9±0.2 | 68.4±2.2 | 41.4±0.5 | 74.5±3.4 |
| NF 270 | 97.2±0.6 | 125.4±2.5 | 97.8±0.4 | 115±2.9 | 58.26±1.1 | 125±4.2 |
| NF 245 | 99.1±0.3 | 55±2.7 | 99.2±0.2 | 52±2.4 | 45.3±1.2 | 57±2.8 |

We have purchased three types of commercial polyamide membrane, GE DK, DOW NF270, NF245, and conducted the performance evaluation. The results comparation is shown in Supplementary Table 9.

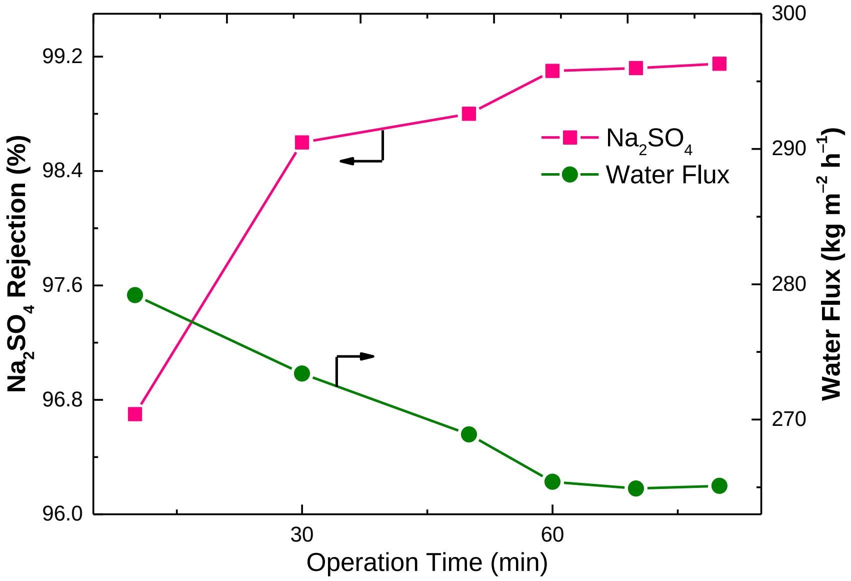


**Supplementary Figure 49.** The variation curve graph of $Na_2SO_4$ rejection and water flux resulting from the compaction effect in the initial separation stage during long-term filtration. The operating pressure and temperature were controlled at 1 MPa, 25°C. The feed flow rate was 7.5 L $min^{-1}$, and the $Na_2SO_4$ concentrations in the feed solutions were 2000 ppm.

It is known that, in the initial stage of the separation experiment, due to the existence of the compaction effect[7–9], the water flux of the polyamide membrane decreased slightly, while the salt rejection rate increased slightly. In this work, as shown in Supplementary Figure 49, during the initial stage (ranged from 0 to 80 min) of the long-term filtration process, the water flux gradually decreases from 279.2 to 265.1 kg $m^{-2}$ $h^{-1}$, while the $Na_2SO_4$ rejection increases from 96.7% to 99.15%.

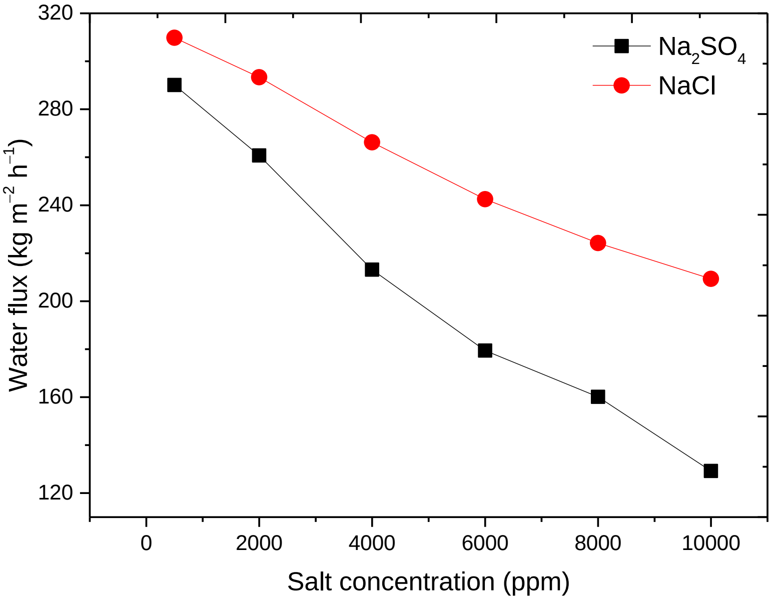


**Supplementary Figure 50.** Influence of salt concentration on the water flux of the asymmetric polyamide membrane. The salt concentration ranges from 500 ppm to 10,000 ppm. The operating pressure and temperature were controlled at 1 MPa, 25°C, and the feed flow rate was 7.5 L min$^{-1}$.

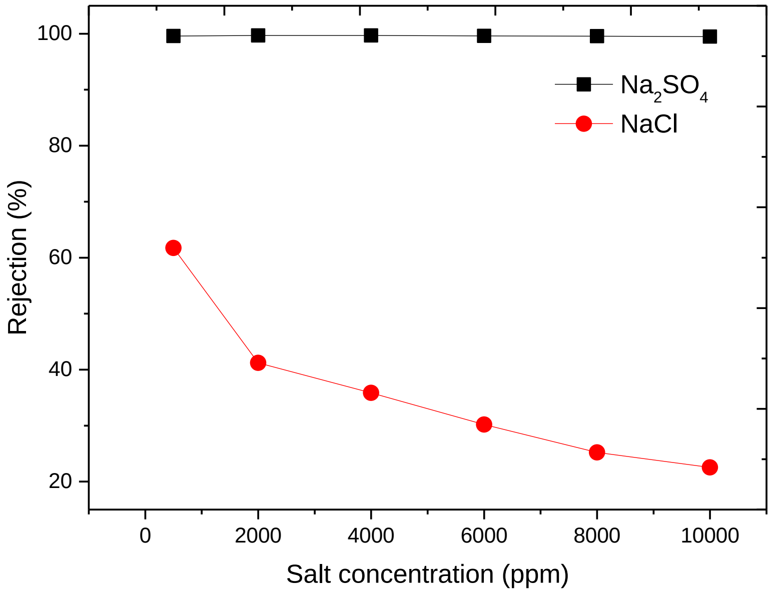


**Supplementary Figure 51.** Influence of salt concentration on the rejection of the asymmetric polyamide membrane. The salt concentration ranges from 500 ppm to 10,000 ppm. The operating pressure and temperature were controlled at 1 MPa, 25°C,

and the feed flow rate was 7.5 L $min^{-1}$.

As shown in Supplementary Figures 50 and 51, we investigated the influence of NaCl and $Na_2SO_4$ concentration on the performance of the asymmetric membrane. As expected, since osmotic pressure increases with the enhancement of the salt concentration, the corresponding water flux gradually decreased from 290.1 kg $m^{-2}$ $h^{-1}$ to 129.3 kg $m^{-2}$ $h^{-1}$ under $Na_2SO_4$ concentration increasing from 500 ppm to 10,000 ppm. For salt rejection, it is noted that the $Na_2SO_4$ rejection maintains between 99.5% and 99.7%, For salt rejection, it is noted that when the salt concentration increases from 500 ppm to 10,000 ppm, $Na_2SO_4$ rejection constantly maintains between 99.5% and 99.7%, while that of NaCl decreases from 61.72% to 22.52%. These results indicate that the asymmetric polyamide NF membrane can achieve the ion selectivity, specifically, the permeability of NaCl is directly proportional to the salt concentration, while the divalent salt exhibits a high rejection rate and has no correlation with the salt concentration.

**Supplementary Table 10.** Ion selectivity of the asymmetric polyamide membrane for different mixed salt solution under optimized operating parameters. Separation experiments were conducted in a cross-flow filtration manner at 25 °C under 1 MPa. The unit of water flux values is kg $m^{-2}$ $h^{-1}$.

| Salt mixture solution | Rejection (%) | | Selectivity |
|---|---|---|---|
| | $Cl^-$ | $SO_4^{2-}$ | $Cl^-/SO_4^{2-}$ |
| 1 g $L^{-1}$ NaCl, 1 g $L^{-1}$ $Na_2SO_4$ | 36.7 | 99.7 | 211 |
| 2 g $L^{-1}$ NaCl, 2 g $L^{-1}$ $Na_2SO_4$ | 23.1 | 99.5 | 153.8 |
| 14.04 mol $m^{-3}$ $Cl^-$, 14.04 mol $m^{-3}$ $SO_4^{2-}$ | 21.37 | 99.9 | 786.3 |
| 28.08 mol $m^{-3}$ $Cl^-$, 28.08 mol $m^{-3}$ $SO_4^{2-}$ | 9.67 | 99.87 | 694.9 |
| 42.14 mol $m^{-3}$ $Cl^-$, 42.14 mol $m^{-3}$ $SO_4^{2-}$ | -1.15 | 99.86 | 722.5 |
| 56.16 mol $m^{-3}$ $Cl^-$, 56.16 mol $m^{-3}$ $SO_4^{2-}$ | -7.38 | 99.84 | 671.1 |
| 70.2 mol $m^{-3}$ $Cl^-$, 70.2 mol $m^{-3}$ $SO_4^{2-}$ | -14.6 | 99.84 | 716.3 |
| 84.24 mol $m^{-3}$ $Cl^-$, 84.24 mol $m^{-3}$ $SO_4^{2-}$ | -16.86 | 99.84 | 730.4 |

We determined the $Cl^-/SO_4^{2-}$ selectivity of the asymmetric polyamide membrane in case of mixed salt solution. As shown in Supplementary Table 10, mixed $Cl^-$ and $SO_4^{2-}$ with equimolar concentration, and mixed NaCl and $Na_2SO_4$ with equal mass volume concentration, were used as feed solution. Ion concentration in the feed solution and permeation solution was measured by the Ion chromatograph (883 Basis IC Plus, Metrohm, Switzerland). It was found that the $SO_4^{2-}$ exhibits a high rejection and has no correlation with salt concentration or ion concentration, while the $Cl^-$ rejection gradually decreased, or even produced negative rejection, with the salt/ion concentration, thus achieving ion screening and showing a high $Cl^-$/ $SO_4^{2-}$ selectivity. $Cl^-/SO_4^{2-}$ Selectivity (S), $S=(100\text{-}R_{Cl^-})/\left(100\text{-}R_{SO_4^{2-}}\right)$ was calculated from the rejection rates of individual anions in the mixed salt solution. For example, for the 84.24 mol $m^{-3}$ (containing 42.14 mol $m^{-3}$ $Cl^-$, 42.14 mol $m^{-3}$ $SO_4^{2-}$) mixed anion solution, we calculated that the $Cl^-$ and $SO_4^{2-}$ rejection is -1.15, 99.86%, respectively, thus the $Cl^-$/ $SO_4^{2-}$ selectivity is up to 722.5. These results that the asymmetric polyamide membrane can reach the ion sieving and used in the industrial wastewater zero liquid discharge process.

**Supplementary Table 11.** Comparison of NaCl rejection in the asymmetric polyamide membrane and reported membrane in literatures. Noted that all membranes in this comparison are fabricated by TMC and PIP.

| Membrane | NaCl Rejection (%) | Water flux permeance (kg $m^{-2}$ $h^{-1}$ $bar^{-1}$) | Test condition | Reference |
|---|---|---|---|---|
| PSF-G4D-IP (Asymmetric) | 41.1 | 30.6 | cross-flow, 1 MPa, 2000 ppm | This work |
| DK | 41.4 | 7.45 | cross-flow, 1 MPa, 2000 ppm | Commercial membrane |
| NF 270 | 58.3 | 12.5 | cross-flow, 1 MPa, 2000 ppm | |
| NF 245 | 45.3 | 5.7 | cross-flow, 1 MPa, 2000 ppm | |
| PES-COF-IP | 27.3 | 56.43 | dead-end, 0.2 MPa, 1000 ppm | 10 |
| PES-CNT-IP | 18.8 | 19 | cross-flow, 0.6 MPa, 2000 ppm | 11 |
| PES-PD/SWCNTs-IP | 22.5 | 40 | cross-flow, 0.6 MPa, 1000 ppm | 12 |
| PSF-Noria/PEI--IP | 43 | 21 | cross-flow, 1 MPa, 2000 ppm | 13 |
| PES-PD/ZIF-8-IP | 11 | 22.34 | cross-flow, 0.4 MPa, 1000 ppm | 14 |
| PES-PD/SWCNT-PA-UIO-66-NH2 | 8.1 | 54 | cross-flow, 0.6 MPa, 1000 ppm | 15 |
| PSF-IP | 49.6 | 25.8 | cross-flow, 0.48 MPa, 2000 ppm | 16 |
| PSF-TA/Fe3+-IP | 18 | 20 | cross-flow, 0.345 MPa, 1000 ppm | 17 |
| MMMs (ZIF-8) | 47.4 | 14.5 | cross-flow, 0.4 MPa, 1000 ppm | 18 |
| PSF-IP | 34.6 | 7.92/0.6 | cross-flow, 0.6 MPa, 2000 ppm | 19 |
| PSF-IP- | 21 | 27/0.6 | cross-flow, 0.6 MPa, 1000 ppm | 20 |

| | | | | |
|---|---|---|---|---|
| PSF-PDA/Ag-IP | 47.1 | 5.9 | cross-flow, 1 MPa, 1000 ppm | 21 |
| HPAN-mZIF/IP | 11.5 | 14.31 | cross-flow, 0.8 MPa, 1000 ppm | 22 |
| PSF-ZNGs/IP | 32.6 | 60.9 | cross-flow, 0.6 MPa, 1000 ppm | 23 |
| PES-MWCNT-OH/IP | 35.3 | 6.9 | cross-flow, 0.6 MPa, 2000 ppm | 24 |
| PES-SWCNT-IP | 13.4 ± 0.8 | 44.17±1.67 | cross-flow, 0.6 MPa, 2000 ppm | 25 |

We have provided a table to compare the NaCl rejection in this work and reported literatures. Three commercial membrane DK, NF 270 and NF 245 were purchased for performance comparison. As shown in Supplementary Table 11, the asymmetric polyamide membrane exhibits a rejection of 41.1% for NaCl, and water flux permeance reaches to 30.6 kg $m^{-2}$ $h^{-1}$ $bar^{-1}$. The NaCl rejection of asymmetric polyamide membrane in this work is the same as some commercial membranes such as DK, but its water flux permeance is far superior than that commercial membranes such as DK, NF 270 and NF 245. More importantly, compared with the reported membranes in literatures, the asymmetric polyamide membrane for NaCl performance including rejection and water flux permeance is higher than most of reported membranes in literatures such as mixed matrix membranes (MMMs).

**Supplementary Table 12.** Separation performance ($MgSO_4$ and $Na_2SO_4$) of the asymmetric polyamide membranes and the commercial polyamide membranes, polyamide membranes prepared by interlayer, crumpled polyamide membranes, mixed matrix polyamide membranes (MMMs) and traditional IP membranes.

| Membrane | Thickness (nm) | Salt | Water permeance (kg $m^{-2}$ $h^{-1}$ $MPa^{-1}$) | Rejection (%) | Reference |
|---|---|---|---|---|---|
| PSF-G4D-IP (Asymmetric) | 70 | $MgSO_4$ | 269.9±8.3 | 99.1 ± 0.3 | This work |
| PSF-G4D-IP (Asymmetric) | 70 | $Na_2SO_4$ | 264±5.1 | 99.2 ± 0.3 | This work |
| PSF-IP | 162 | $Na_2SO_4$ | 71.4±2.4 | 96 ± 1.8 | This work |
| PSF-IP | 162 | $MgSO_4$ | 68.5±2.8 | 93.4 ± 1.6 | This work |
| PES-COF-IP | 7 | $Na_2SO_4$ | 535.5 | 94.3 | 10 |
| PES-CNTs-IP | 29±2 | $MgSO_4$ | 120 | 98.3 | 11 |
| PES-CNTs-IP | 29±2 | $Na_2SO_4$ | 140 | 98.5 | 11 |
| PES-PD/SWCNTs-IP | 12 | $Na_2SO_4$ | 320 | 95.9 | 12 |
| PES-PD/SWCNTs-IP | 12 | $MgSO_4$ | 330 | 94.1 | 12 |
| PSF-Noria/PEI-IP | 32.7 | $MgSO_4$ | 275 | 96 | 13 |
| PSF-Noria/PEI-IP | 32.7 | $Na_2SO_4$ | 276 | 96.8 | 13 |
| PES-PD/ZIF-8-IP | 8-14 | $Na_2SO_4$ | 532±2 | 95.2±0.2 | 14 |
| PSF-IP | 20 | $Na_2SO_4$ | 247.9 | 99.6 | 16 |
| PSF-IP | 20 | $MgSO_4$ | 260 | 99.2 | 16 |
| PSF-IP | 30−50 | $Na_2SO_4$ | 127 | 99.1 | 16 |
| PSF-IP | 30−50 | $MgSO_4$ | 131.25 | 98.5 | 16 |
| PSF-TA/$Fe^{3+}$-IP | 54.9 ± 1.8 | $Na_2SO_4$ | 196±5 | 98 | 17 |
| MMMs | 98.5 | $Na_2SO_4$ | 194 | 95.2 | 18 |
| PSF-IP | 140.03 | $Na_2SO_4$ | 114 | 98.3 | 19 |
| PSF-IP | 140.03 | $MgSO_4$ | 106 | 95 | 19 |
| MMMs | - | $Na_2SO_4$ | 143.25 | 96 | 22 |
| MMMs | 77 | $MgSO_4$ | 59 | 97.1 | 24 |
| MMMs | 77 | $Na_2SO_4$ | 68 | 97.6 | 24 |
| PES-SWCNT-IP | 15 | $Na_2SO_4$ | 403.3± 13.3 | 96.5 ± 0.7 | 25 |
| PES-SWCNT-IP | 15 | $MgSO_4$ | 395±8.3 | 95.3 ± 0.2 | 25 |
| UiO-66 TFs | 400 | $Na_2SO_4$ | 14.1 | 96 | 26 |
| uGNMs (GO) | ≈ 22–53 | $Na_2SO_4$ | 218 | 60 | 27 |
| MMMs (ZCNT) | 245 | $Na_2SO_4$ | 14.5 | 99.9 | 28 |
| PES-IP | 32 | $MgSO_4$ | 177±6 | 97±1.0 | 29 |
| PES-IP | 32 | $Na_2SO_4$ | 201±5 | 99±0.2 | 29 |

| | | | | | |
|---|---|---|---|---|---|
| Polyelectrolyte | - | $Na_2SO_4$ | 45 | 98 | 30 |
| PES-IP | 15 | $Na_2SO_4$ | 86.8±0.61 | 99.1±0.13 | 31 |
| PES-IP | 15 | $MgSO_4$ | 88.3± 0.24 | 99.4± 0.04 | 31 |
| PSF-IP (NF 270) | 50 | $MgSO_4$ | 119.4±2.5 | 97.2±0.6 | 32 |
| PSF-IP (NF 270) | 50 | $Na_2SO_4$ | 115±2.9 | 97.8±0.4 | 32 |
| MMMs | 86 | $Na_2SO_4$ | 155.36 | 99.1 | 33 |
| IP | 220 | $MgSO_4$ | 55 | 98.5 | 34 |
| IP | 220 | $Na_2SO_4$ | 55 | 98.3 | 34 |
| MMMs | 70.1 | $MgSO_4$ | 140.25 | 97.6 | 35 |
| MMMs | 114 | $Na_2SO_4$ | 164 | 97.3 | 36 |
| PSF-Polyphenol-IP | 56.96 | $Na_2SO_4$ | 100 | 98 | 37 |
| PAN-PDA/COF-IP | 11 | $Na_2SO_4$ | 207.07 | 93.4 | 38 |
| PAN-PDA/COF-IP | 11 | MgSO4 | 210 | 91 | 38 |
| MMMs | 167 | $Na_2SO_4$ | 86 | 93.9 | 39 |
| PSF-IP | 174 | $MgSO_4$ | 76 | 98.5 | 40 |
| PSF-IP | 174 | $Na_2SO_4$ | 80 | 99.1 | 40 |
| PSF-TA/DETA-IP | 56.96± 4.4 | $Na_2SO_4$ | 75 | 98 | 41 |

**Reference**


1. Fujiwara, M., Imura, T. Photo Induced Membrane Separation for Water Purification and Desalination Using Azobenzene Modified Anodized Alumina Membranes. *ACS nano* **9**, 5705–5712 (2015).
2. Le, X. T. *et al.* Diazonium-induced anchoring process: an application to improve the monovalent selectivity of cation exchange membranes. *J. Mater. Chem.* **20**, 3750–3757 (2010).
3. Lin, L., Feng, C., Lopez, R., Coronell, O. Identifying facile and accurate methods to measure the thickness of the active layers of thin-film composite membranes – A comparison of seven characterization techniques. *J. Membr. Sci.* **498**, 167–179 (2016).
4. Michaels, A. S. Analysis and Prediction of Sieving Curves for Ultrafiltration Membranes: A Universal Correlation? *Sep. Purif. Technol.* **15**, 1305–1322 (1980).
5. Shardul, S. Wadekar, R. D. V. Influence of Active Layer on Separation Potentials of Nanofiltration Membranes for Inorganic Ions. *Environ. Sci. Tech.* **51**, 5658–5665 (2017).
6. Childress, A. E., Elimelech, M. Effect of solution chemistry on the surface charge of polymeric reverse osmosis and nanofiltration membranes. *J. Membr. Sci.* **119**, 253–268 (1996).
7. Pendergast, M. T. M., Nygaard, J. M., Ghosh, A. K., Hoek, E. M. V. Using nanocomposite materials technology to understand and control reverse osmosis membrane compaction. *Desalination* **261**, 255–263 (2010).
8. Hussain, Y. A., Al-Saleh, M. H., Ar-Ratrout, S. S. The effect of active layer non-uniformity on the flux and compaction of TFC membranes. *Desalination* **328**, 17–23 (2013).
9. Davenport, D. M. et al. Thin film composite membrane compaction in high-pressure reverse osmosis. *J. Membr. Sci.* **610**, 118268 (2020).
10. Yuan, J. et al. Covalent organic framework-modulated interfacial polymerization for ultrathin desalination membranes. *J. Mater. Chem. A* **7**, 25641–25649 (2019).
11. Gong, G., Wang, P., Zhou, Z., Hu, Y. New Insights into the Role of an Interlayer for

the Fabrication of Highly Selective and Permeable Thin-Film Composite Nanofiltration Membrane. *ACS. Appl. Mater. Inter.* **11**, 7349–7356 (2019).

12. Zhu, Y. et al. Single-Walled Carbon Nanotube Film Supported Nanofiltration Membrane with a Nearly 10 nm Thick Polyamide Selective Layer for High-Flux and High-Rejection Desalination. *Small* **12**, 5034–5041 (2016).
13. Zhai, Z. et al. Fabrication of advanced nanofiltration membranes with nanostrand hybrid morphology mediated by ultrafast Noria–polyethyleneimine codeposition. *J. Mater. Chem. A* **6**, 21207–21215 (2018).
14. Wang, Z. et al. Nanoparticle-templated nanofiltration membranes for ultrahigh performance desalination. *Nat. Commun.* **9**, 2004 (2018).
15. Gong, Y. *et al.* Thin-film nanocomposite nanofiltration membrane with an ultrathin polyamide/UIO-66-NH2 active layer for high-performance desalination. *J. Membr. Sci.* **600**, 117874 (2020).
16. Tan, Z., Chen, S., Peng, X., Zhang, L., Gao, C. Polyamide membranes with nanoscale Turing structures for water purification. *Science* **360**, 518–521 (2018).
17. Yang, Z. et al. Tannic Acid/Fe3+ Nanoscaffold for Interfacial Polymerization: Toward Enhanced Nanofiltration Performance. *Environ. Sci. Technol.* **52**, 9341–9349 (2018).
18. Liao, Z. *et al.* Hydrophilic Hollow Nanocube-Functionalized Thin Film Nanocomposite Membrane with Enhanced Nanofiltration Performance. *ACS. Appl. Mater. Inter.* **11**, 5344–5352 (2019).
19. Zhang, H-L., Gao, Y-B., Gai, J-G. Guanidinium-functionalized nanofiltration membranes integrating anti-fouling and antimicrobial effects. *J. Mater. Chem. A* **6**, 6442–6454 (2018).
20. Peng, H. *et al.* Phosphonium Modification Leads to Ultrapermeable Antibacterial Polyamide Composite Membranes with Unreduced Thickness. *Adv. Mater.* **32**, 2001383 (2020).
21. Yang, Z. *et al.* A novel thin-film nano-templated composite membrane with in situ silver nanoparticles loading: Separation performance enhancement and

implications. *J. Membr. Sci.* **544**, 351–358 (2017).

22. Zhu, J. *et al.* Elevated Performance of Thin Film Nanocomposite Membranes Enabled by Modified Hydrophilic MOFs for Nanofiltration. *ACS. Appl. Mater. Inter.* **9**, 1975–1986 (2017).
23. Ji, Y-L., An, Q-F., Weng, X-D., Hung, W-S., Lee, K-R., Gao, C-J. Microstructure and performance of zwitterionic polymeric nanoparticle/polyamide thin-film nanocomposite membranes for salts/organics separation. *J. Membr. Sci.* **548**, 559–571 (2018).
24. Xue, S. M., Xu, Z. L., Tang, Y. J., Ji, C. H. Polypiperazine-amide Nanofiltration Membrane Modified by Different Functionalized Multiwalled Carbon Nanotubes (MWCNTs). *ACS. Appl. Mater. Inter.* **8**, 19135–19144 (2016).
25. Gao, S., Zhu, Y., Gong, Y., Wang, Z., Fang, W., Jin, J. Ultrathin Polyamide Nanofiltration Membrane Fabricated on Brush-Painted Single-Walled Carbon Nanotube Network Support for Ion Sieving. *ACS nano* **13**, 5278–5290 (2019).
26. Liu, T-Y., Yuan, H-G., Liu. Y-Y., Ren, D., Su, Y-C., Wang, X. Metal–Organic Framework nanocomposite thin films with interfacial bindings and self-standing robustness for high water flux and enhanced ion selectivity. *ACS nano* **12**, 9253–9265 (2018).
27. Han, Y., Xu, Z., Gao, C. Ultrathin graphene nanofiltration membrane for water purification. *Adv. Funct. Mater.* **23**, 3693–3700 (2013).
28. Liu, T. Y. et al. Ion-responsive channels of zwitterion-carbon nanotube membrane for rapid water permeation and ultrahigh mono-/multivalent ion selectivity. *ACS nano* **9**, 7488–7496 (2015).
29. Yuan, B. et al. Semi-aromatic polyamide nanofiltration membranes with tuned surface charge and pore size distribution designed for the efficient removal of Ca2+ and Mg2+. *Sep. Purif. Technol.* **220**, 162–175 (2019).
30. de Grooth, J., Reurink, D. M., Ploegmakers, J., de Vos W. M., Nijmeijer, K. Charged micropollutant removal with hollow fiber nanofiltration membranes based on polycation/polyzwitterion/polyanion multilayers. *ACS. Appl. Mater. Inter.* **6**,

17009–17017 (2014).

31. Yuan, B. et al. Ultrathin polyamide membrane with decreased porosity designed for outstanding water-softening performance and superior antifouling properties. *ACS. Appl. Mater. Inter.* **10**, 43057–43067 (2018).
32. Mohammad, A. W., Teow, Y. H., Ang, W. L., Chung, Y. T., Oatley-Radcliffe, D. L., Hilal, N. Nanofiltration membranes review: Recent advances and future prospects. *Desalination* **356**, 226–254 (2015).
33. Kong, X. et al. High permselectivity hyperbranched polyester/polyamide ultrathin films with nanoscale heterogeneity. *J. Mater. Chem. A* **5**, 7876–7884 (2017).
34. Chen, Y., Liu, F., Wang, Y., Lin, H., Han, L. A tight nanofiltration membrane with multi-charged nanofilms for high rejection to concentrated salts. *J. Membr. Sci.* **537**, 407–415 (2017).
35. Zhang, H-Z., Xu, Z-L., Ding, H., Tang, Y-J. Positively charged capillary nanofiltration membrane with high rejection for Mg2+ and Ca2+ and good separation for Mg2+ and Li+. *Desalination* **420**, 158–166 (2017).
36. Pan, Y., Xu, R., Lü, Z., Yu, S., Liu, M., Gao, C. Enhanced both perm-selectivity and fouling resistance of poly(piperazine-amide) nanofiltration membrane by incorporating sericin as a co-reactant of aqueous phase. *J. Membr. Sci.* **523**, 282–290 (2017).
37. Zhang, X., Lv, Y., Yang, H-C., Du, Y., Xu, Z-K. Polyphenol coating as an interlayer for thin-film composite membranes with enhanced nanofiltration performance. *ACS. Appl. Mater. Inter.* **8**, 32512–32519 (2016).
38. Wu, M. et al. Ultrathin nanofiltration membrane with polydopamine-covalent organic framework interlayer for enhanced permeability and structural stability. *J. Membr. Sci.* **576**, 131–141 (2019).
39. Wu, M. et al. Fabrication of composite nanofiltration membrane by incorporating attapulgite nanorods during interfacial polymerization for high water flux and antifouling property. *J. Membr. Sci.* **544**, 79–87 (2017).
40. Shi, Q., Ni, L., Zhang, Y., Feng, X., Chang, Q., Meng, J. Poly(p-phenylene

terephthamide) embedded in a polysulfone as the substrate for improving compaction resistance and adhesion of a thin film composite polyamide membrane. *J. Mater. Chem. A* **5**, 13610–13624 (2017).

41. Zhang, X., Lv, Y., Yang, H-C., Du, Y., Xu, Z-K. Polyphenol Coating as an Interlayer for Thin-Film Composite Membranes with Enhanced Nanofiltration Performance. *ACS. Appl. Mater. Inter.* **8**, 32512–32519 (2016).
